# Targeting telomerase with radiolabeled inhibitors

**DOI:** 10.1016/j.ejmech.2016.09.028

**Published:** 2017-01-05

**Authors:** Philip A. Waghorn, Mark R. Jackson, Veronique Gouverneur, Katherine A. Vallis

**Affiliations:** a CR-UK/MRC Oxford Institute for Radiation Oncology, University of Oxford, Old Road Campus Research Building, Off Roosevelt Drive, Oxford, OX3 7DQ, UK; b Chemistry Research Laboratory, University of Oxford, 12 Mansfield Road, Oxford, OX1 3TA, UK

**Keywords:** Telomerase, Telomerase inhibitors, Targeted radionuclide therapy, Iodine-123

## Abstract

The expression of telomerase in approximately 85% of cancers and its absence in the majority of normal cells makes it an attractive target for cancer therapy. However the lag period between initiation of telomerase inhibition and growth arrest makes direct inhibition alone an insufficient method of treatment. However, telomerase inhibition has been shown to enhance cancer cell radiosensitivity. To investigate the strategy of simultaneously inhibiting telomerase while delivering targeted radionuclide therapy to cancer cells, ^123^I-radiolabeled inhibitors of telomerase were synthesized and their effects on cancer cell survival studied. An ^123^I-labeled analogue of the telomerase inhibitor MST-312 inhibited telomerase with an IC_50_ of 1.58 μM (MST-312 IC_50_: 0.23 μM). Clonogenic assays showed a dose dependant effect of ^123^I-MST-312 on cell survival in a telomerase positive cell line, MDA-MB-435.

## Highlights

• ^123^I-radiolabeled small molecule inhibitors of telomerase were synthesized.
• Synchronous telomerase inhibition and radionuclide therapy was achieved.
• ^123^I-MST-312 caused cytotoxicity in telomerase positive cancer cells.

## Graphical abstract

**Figure:**
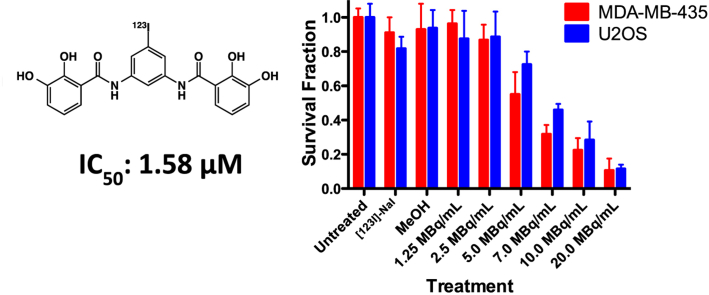

## 1 Introduction

Preclinical and clinical evidence supports the inhibition of telomerase as a promising form of cancer therapy [1], [2]. Telomerase is present in the majority (85–90%) of cancers cell lines, and is responsible for rescuing them from crisis, and promoting uncontrolled and continued tumor growth [1], [3], [4]. Telomerase inhibition disrupts the replicative capacity of cancer cells by preventing maintenance of the telomeric sequences at the chromosome terminus, while leaving most normal somatic cells largely unaffected. Telomerase activity is minimally reconstituted *in vitro* by combining the human telomerase RNA template component (hTR) [5] and the catalytic protein unit, human telomerase reverse transcriptase (hTERT) [6].

The hTR template region provides an accessible substrate-binding site allowing for direct enzyme inhibition using antisense oligonucleotides, peptide nucleic acids (PNAs) and chemically modified PNAs as competitive inhibitors, preventing active complex formation with the hTERT component or binding to the telomere substrate [7]. GRN163L (Imetelstat) is a lipid-conjugated N3′-P5′ thio-phosphoramidate 13-mer oligonucleotide that has been shown to inhibit telomerase and cause telomere shortening in cells from brain, bladder, liver, lung, prostate and stomach cancers [8], [9], [10], [11], [12], [13], [14].

Although hTR is expressed ubiquitously, telomerase activity is restricted by the expression of the hTERT component [15], [16]. Numerous small molecule inhibitors of telomerase have been identified [17], [18], [19], [20]. Most notably BIBR-1532 [21], [22], where dose-dependent inhibition of telomerase with increasing concentrations of BIBR-1532 has been shown, without significant effects on normal cells [23]. Other inhibitors include azidothymidine [24], [25], the epicatechin derivatives, EGCG and MST-312 that strongly and directly inhibit telomerase [26], [27], [28], isothiazolone and bis-indole derivatives [29], [30], and several G-quadruplex stabilizing molecules [31], [32], [33].

Several clinical trials are currently underway, targeting both the telomeres and telomerase function. Clinical trials with Imetelstat for haematological malignancies (essential thrombocythemia (ET), myelodysplastic syndrome, acute myelogenous leukaemia) and myelofibrosis (MF) are planned, underway or completed [34]. So far phase II trials for ET and MF have found no correlation between clinical response and telomere length [35]. Currently phase I/II clinical trials with the oncolytic virus, OBP-301, are underway in patients with hepatocellular carcinoma. In phase I testing OBP-301 was well tolerated with no serious adverse effects [36]. The cancer vaccine, GV1001, a TERT derived peptide for telomerase driven immunotherapy is involved in several clinical trials in non-small cell lung cancer (NSCLC), pancreatic cancer, hepatocellular carcinoma and malignant melanoma, where few side effects have been reported [37]. In phase I/II NSCLC studies a GV1001-specific immune response was observed [38]. In a phase III trial in pancreatic cancer, however, no improvement in overall survival was observed [39]. Although BIBR-1532, MST-312 and several G-quadruplex inhibitors have had success in preclinical testing they have not yet entered into clinical trials. The G-quadruplex stabilizer Quarfloxin/CX-3543 has entered phase I and II trials but is thought to induce apoptosis through inhibition of ribosomal RNA (rRNA) [40]. Several tankyrase inhibitors such as XAV939, which disrupt telomere length regulation are being tested as treatment strategies but have not yet entered clinical trials [41]. Despite significant insights into the role of telomerase in disease there is still no agent yet approved for clinical use [42].

The relationship between cellular radiosensitivity and telomere length is one that has been investigated extensively [43], [44], [45], [46], [47]. Goytisolo et al. reported the connection between shortenened telomeres in late generation mTR−/− mice and radiation response, evident as organism hypersensitivity to IR and increased DNA damage after irradiation [48]. Similarly Wong et al. have shown that telomerase inhibition and telomere dysfunction in fibroblasts from late generation Terc−/− mice imparts an enhanced radiosensitivity associated with increased mortality [49], [50], [51]. Similar studies have shown enhanced radiosensitivity in mice where telomeres have been shortened by mutant hTERT expression [44], [45], [52], [53]. Increased telomerase expression has been associated with enhanced genome stability and DNA repair mechanisms, providing a protective mechanism against DNA damage [54], [55].

Radiolabeled agents that specifically inhibit telomerase activity would be expected, therefore, to selectively increase radiosensitivity and so increase tumor cell kill [56]. We report here the synthesis of a series of small molecule telomerase inhibitors, the protocols for radiolabeling them with the Auger electron-emitting isotope, ^123^I, and their effect on telomerase inhibition and cancer cell survival.

## 2 Results and discussion

**Fig. 1:**
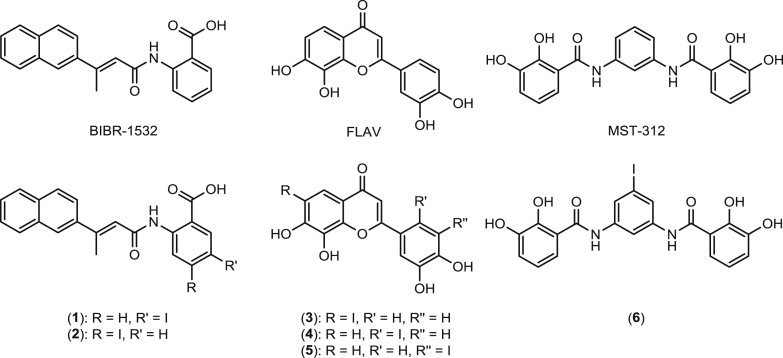
Structures of telomerase inhibitors; BIBR-1532, Flavonoid (FLAV) and MST-312, and iodinated derivatives (**1–6**).

The telomerase inhibitory capabilities of BIBR-1532, MST-312 and the flavonoid species 2-(3,4-dihydroxyphenyl)-7,8-dihydroxy-4H-chromen-4-one (Fig. 1) have been directly compared under the same experimental conditions obtaining IC_50_ values of 3.6, 12.1 and 0.23 μM, respectively [57]. Structure activity relationship studies with the BIBR-1532 and flavonoid species have shown that certain site specific structural modifications to these parent structures have only minimal effects on telomerase inhibition, suggesting inclusion of an Iodine-123 radiolabel modification to allow for combined targeted therapy would have limited effect on telomerase inhibition in these species. As well as decaying by release of high energy, short pathlength Auger electrons, ^123^I was selected for site-specific inclusion in these molecules to minimize the structural alterations that would be encountered with metal radioisotopes.

### 2.1 Chemistry

**Scheme 1:**
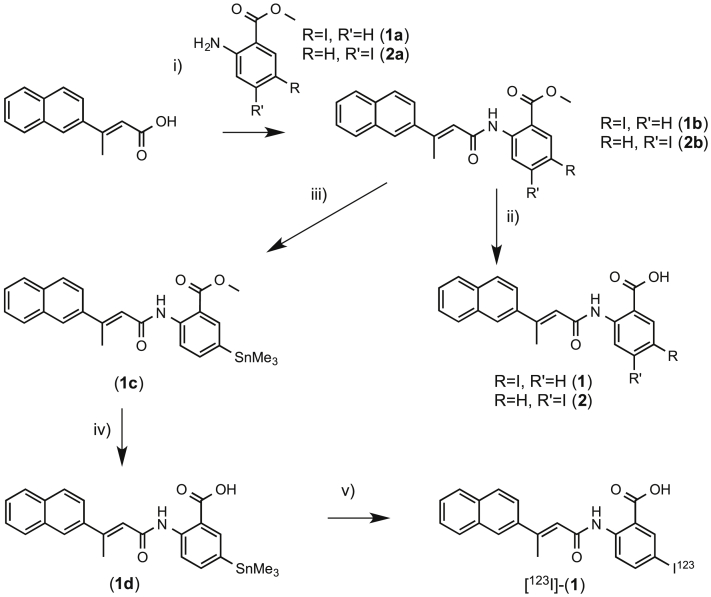
i) a) oxalyl chloride, DMF, DCM, 5 h 0 °C, b) **1a/2a**, pyridine, DMAP, THF, 2 h, r.t.; ii) LiOH, THF, H_2_O, 12 h, r.t.; iii) Sn_2_Me_6_, PdCl_2_(PPh_3_)_2_, 1,4-dioxane, 2 h, 60 °C; iv) LiOH, THF, H_2_O, 12 h, r.t.; v) [^123^I]NaI, Chloramine T, MeCN, 15 min, r.t.

**Scheme 2:**
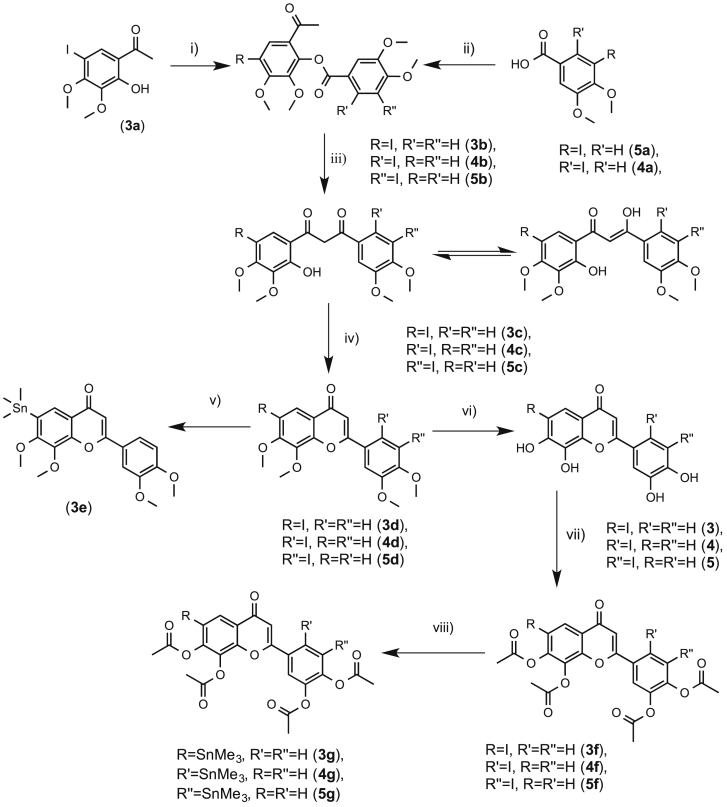
i) 3,4-dimethoxybenzoyl chloride, pyridine, 2 h, r.t.; ii) a) oxalyl chloride, DMF, DCM, 2 h 0 °C, b) 3,4-dimethoxy-2-hydroxyacetophenone, pyridine, DCM, 2 h, r.t.; iii) KOH, pyridine, 1 h, 50 °C; iv) NaOAc, AcOH, 2 h, 120 °C; v) Sn_2_Me_6_, PdCl_2_(PPh_3_)_2_, 1,4-dioxane, 1.5 h, 60 °C; vi) BBr_3_, DCM, 2 h, 0 °C – r.t.; vii) Ac_2_O, pyridine, 20 h, 45 °C; viii) Sn_2_Me_6_, PdCl_2_(PPh_3_)_2_, 1,4-dioxane, 1.5 h, 60 °C.

**Scheme 3:**
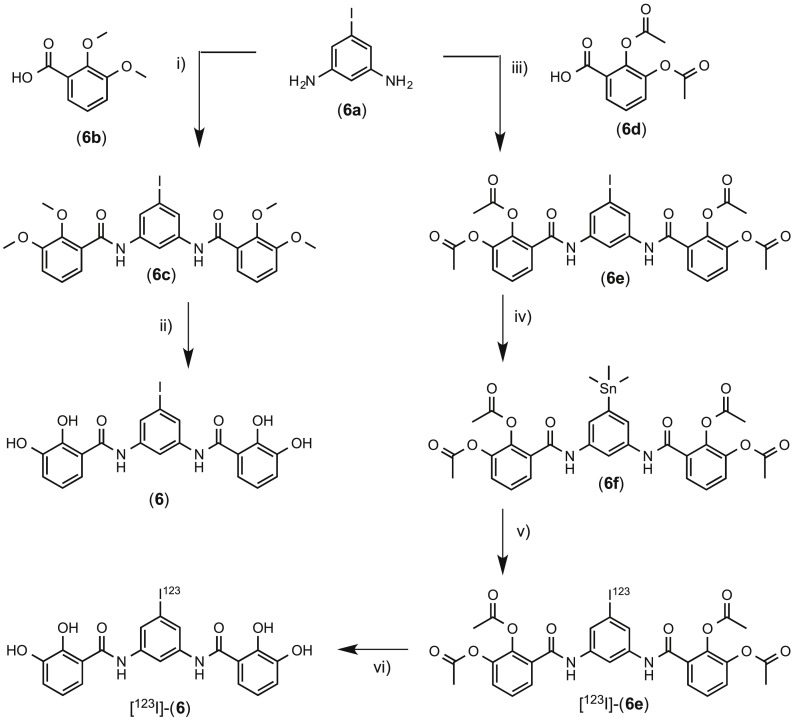
i) a) **6b**, oxalyl chloride, DMF, DCM, 2 h 0 °C, b) **6a**, pyridine, DCM, 2 h, r.t.; ii) BBr_3_, DCM, 2 h, 0 °C – r.t.; iii) a) **6d**, oxalyl chloride, DMF, DCM, 2 h 0 °C; b) **6a**, pyridine, DCM, 2 h, r.t.; iv) Sn_2_Me_6_, PdCl_2_(PPh_3_)_2_, 1,4-dioxane, 12 h, 75 °C; v) [^123^I]NaI, H_2_O_2_:HOAc, MeCN, 15 min, r.t.; vi) 3 M HCl, MeOH, 0.5 h, 80 °C.

In Fig. 1 the chemical structures of the iodinated analogues and their parent precursors, tested for *in vitro* telomerase inhibition, are reported. Scheme 1, Scheme 2, Scheme 3 show the synthetic pathways employed in the preparation of the iodinated derivatives **1–6**. The iodinated BIBR-1532 analogues (Scheme 1) were prepared using an adapted literature protocol [58]. Briefly, the condensation reaction of the acid chloride of (*E*)-3-(naphthalen-2-yl)but-2-enoic acid with either the commercially available 5-iodo-2-aminobenzoate or with 4-iodo-2- aminobenzoate prepared from *N*-(4-iodo-2-methyl-phenyl)-acetamide in 2 steps gave **1b** and **2b** in 72% and 67% yield respectively.

Base hydrolysis of **1b** and **2b** gave **1** and **2** in 93% and 95% yield respectively. The trimethyltin analogue was prepared by reaction of **1b** with hexamethyldistannane and *bis*-(triphenylphosphine)-palladium(II)-dichloride in 1,4-dioxane to afford **1c** after column chromatography. Base hydrolysis of **1c** gave **1d** in 87% yield.

The iodinated flavonoids (Scheme 2) were prepared using the Baker-Venkataraman transformation following an adapted literature protocol. An appropriate hydroxyacetophenone was reacted with an appropriate analogue of 3,4-dimethoxybenzoyl chloride to yield a benzoyl ester that, after treatment with base, yields a 1,3-diketone. Treatment of the diketone in acid affords the protected flavone and demethylation achieved using 1 M boron tribromide in dichloromethane at 0 °C yields the flavone product. In the case of **3**, iodinated hydroxyacetophenone **3a** was prepared by reaction of 2,3-dimethoxy-2-hydroxyacetophenone with *N*-iodosuccinimide and *p*-toluenesulphonic acid in acetonitrile [59].

**3a** was subsequently reacted with the commercially available 3,4-dimethoxybenzoyl chloride to give **3b** which was converted to the 1,3-diketone, **3c**. Cyclodehydration was achieved in acetic acid to give **3d** and demethylation gave **3**. For **4** and **5**, 3,4-dimethoxy-2-hydroxyacetophenone was reacted with the acid chloride of either the commercially available 2-iodo-4,5-dimethoxybenzoic acid to give **4b** or with 3-iodo-4,5- dimethoxybenzoic acid prepared from 3-iodo-4-hydroxy-5-methoxybenzaldehyde in 2 steps to give the benzoyl ester **5b**. Subsequent steps as for **3** afforded **4** and **5**.

The trimethyltin analogue of **3** was prepared by first synthesizing the acetyl protected analogue by reaction of **3** with acetic anhydride in pyridine. Then **3g** was prepared by reaction of **3f** with hexamethylditin and *bis*-(triphenylphosphine)-palladium(II)-dichloride to give **3g** in 58% yield. **4g** and **5g** were prepared in identical fashion.

The iodinated MST-312 analogue **6** (Scheme 3) was prepared by the condensation of **6a** with the acid chloride of **6b**, followed by demethylation with boron tribromide. The stannane analogue was prepared by the condensation of **6a** with the acid chloride of **6d**, followed by reaction with hexamethylditin and *bis*-(triphenylphosphine)-palladium(II)-dichloride to give **6f** in 26% yield.

### 2.2 Telomerase inhibition

Telomerase Repeat Amplification Protocol (TRAP) measurements have demonstrated that the iodinated compounds were able to directly inhibit telomerase activity in a cell free system with minimally reduced inhibitory potencies compared to their respective parent compounds as a result of inclusion of the iodide substituent. Compounds **1** and **2** were shown to have IC_50_ values of 30.09 μM and 28.08 μM respectively, corresponding to a ∼2.5 fold reduction in telomerase inhibitory activity with respect to the parent BIBR-1532 compound (IC_50_: 11.57 μM). The iodinated flavonoid isomers **3** and **5** displayed telomerase inhibitory capabilities with IC_50_ values of 1.65 and 1.73 μM respectively, similarly corresponding to a 2.0–2.2 fold reduction in telomerase inhibitory activity with respect to the parent flavonoid compound (IC_50_: 0.74 μM). The IC_50_ values measured for BIBR-1532 and the parent flavonoid, 7,8,3′,4′-tetrahydroxyflavone, differ from those previously reported in the literature, which we ascribe to variations in method and the cell line that was used. Compound **4** was found not to inhibit telomerase activity to the same extent as **3** and **5** (See Supporting Information) and as such further radiolabeling studies with **4** were not conducted. Compound **6** displayed no telomerase inhibitory activity under the described protocol, however under these experimental conditions the parent MST-312 compound similarly failed to inhibit telomerase in the TRAP setup that was used in this study. TRAP experiments were repeated with compound **6** and parent MST-312 compound using a range of alternative telomerase positive cell lysates but no positive inhibition with MST-312 was observed, suggesting that direct inhibition of telomerase is not the method of inhibition in these cell lines. Indirect effects such as signaling or changes in expression levels, or the need of metabolic activation of MST-312 in cells may be required for telomerase inhibition. To address this, cells were plated in six well plates and treated with different concentrations of either parent MST-312 or **6** over a 24 h incubation period. Cell lysates for each treated sample were prepared as described in the Supporting Information and the TRAP assay performed on the individually prepared lysate samples. Results indicate a concentration dependent inhibition of telomerase activity after *in vitro* treatment of whole cells with the MST compounds, with the iodinated species **6** having an IC_50_ of 1.58 μM compared with 0.23 μM for the parent MST-312 compound. In all cases the inclusion of an iodide substituent led to only a slight reduction in IC_50_ values, maintaining the possibility for potential telomerase inhibition and targeting (Table 1).

The lag period required after telomerase inhibition to observe critical telomere shortening and cell crisis suggests that telomerase inhibition alone may be insufficient as a stand-alone treatment. The presence of components of the DNA damage response pathways at the telomeres together with a reported telomere-independent role for telomerase in DNA repair suggests that a combination of telomere inhibition and sensitivity to ionizing radiation (IR) may be potentially interacting [60]. We therefore prepared the radiolabelled analogues of the iodinated inhibitors to investigate further the effect of a combined radiotherapeutic with telomerase inhibition.

### 2.3 Radioiodination

**Fig. 2:**
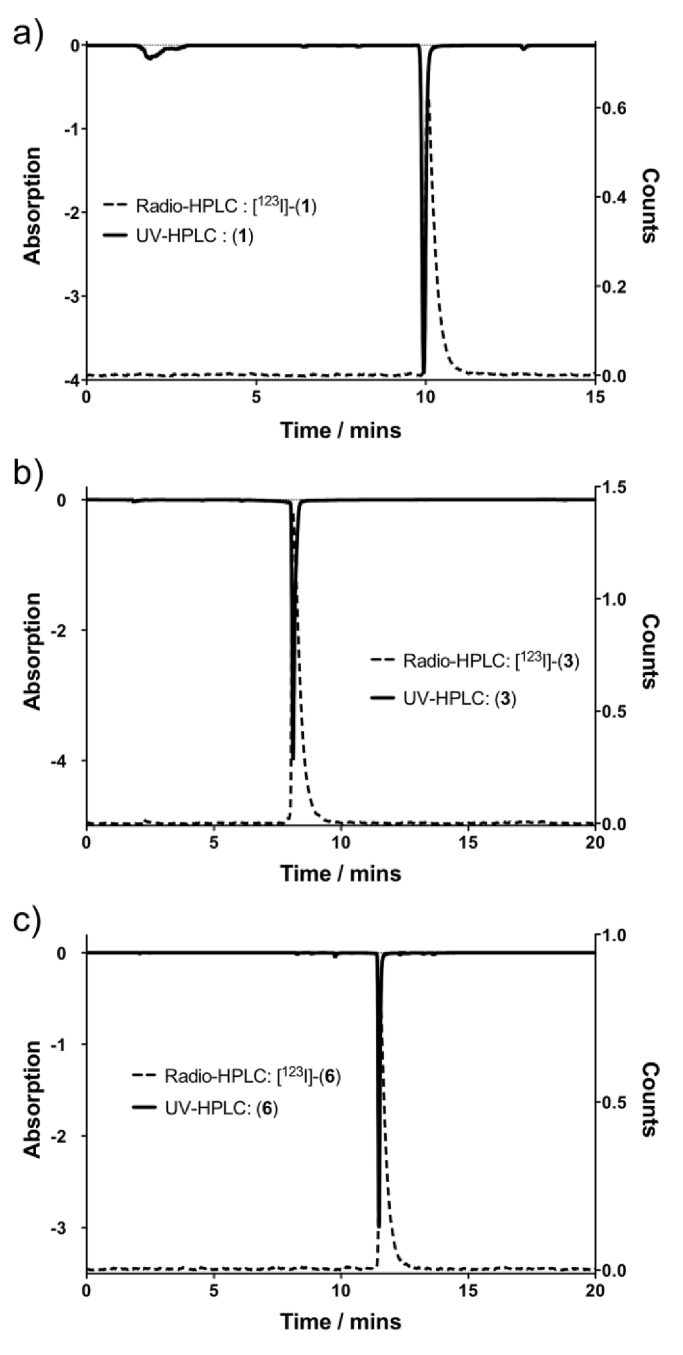
Radiotrace and UVtrace for a) [^123^I]-(**1**), b) [^123^I]-(**3**) and c) [^123^I]-(**6**).

To regioselectively prepare radioiodinated agents trimethyltin precursors were first prepared. In the case of the BIBR-1532 derivative the trimethyltin complex **1d** could not be isolated as a pure species by direct stannylation of the iodinated analogue, (**1**). Instead the trimethyltin complex of **1b** was prepared which was stable to column chromatography on neutral silica and **1d** prepared subsequently by basic hydrolysis of **1c**, following workup with careful control of pH to avoid proto-destannylation. [^123^I]-(**1**) was subsequently radiolabeled using Chloramine T (*N*-chloro-*p*-toluenesulfonamide sodium salt) as an oxidant with a decay corrected isolated end of synthesis yield of 76% ± 6% (n = 10) and a radiochemical purity as assessed by analytical radio-HPLC of >97%, with a specific activity of 6.08 ± 0.28 Ci/μmol (Fig. 2a). Attempts to prepare a trimethyltin derivative directly from the parent compound, **3**, met with poor yields and difficulties in purification. Accordingly a trimethyltin analogue **3e** was prepared from the methoxy protected precursors necessitating the inclusion of a subsequent deprotection step following successful radiolabeling. Although radiolabeling was possible in good yields (72%), subsequent deprotection attempts of the four methoxy groups using HI or BBr_3_ as per the cold synthesis, met with limited success resulting in low yields and with multiple side products seen by radio-HPLC analysis. To improve labeling yields alternative protecting group strategies were sought and the acetylated analogue, **3g**, was prepared accordingly. Again the initial radiolabeling step was performed in good to high yields, and following treatment with 3 M HCl at elevated temperatures deprotection of the four acetyl groups was achieved in good yield. Attempts to deprotect the acetyl groups under basic conditions led to complete deiodination of the flavonoid species. [^123^I]-(**3**) was radiolabeled in a yield of 86 ± 8% (n = 10) and following deprotection was prepared with a decay corrected end of synthesis yield of 64 ± 4% (n = 10) and with a radiochemical purity as assessed by analytical radio-HPLC of >98%, with a specific activity of 2.60 ± 0.17 Ci/μmol (Fig. 2b).

[^123^I]-(**5**) was labeled in a similar fashion with an end of synthesis yield of 67% ± 6% (n = 10) and with a radiochemical purity of >98%. The trimethyltin derivative, **6f** was prepared in similar fashion to the flavonoid species. A range of oxidants was explored and labeling optimized with hydrogen peroxide in acetic acid giving a radiolabeling yield of 55 ± 3% (n = 6). Following deprotection and subsequent HPLC and Seppak cartridge purification steps a decay corrected end of synthesis yield of 22 ± 5% was obtained, with a radiochemical purity >97%, and a specific activity of 2.89 ± 0.24 Ci/μmol.

The lipophilicity of compounds can affect their tissue permeability properties, which can impact their localization in target tissues. Lipophilicity may also affect binding to low affinity nonspecific sites that can compromise target tissue to background tissue ratios. The octanol/water and octanol/PBS partition coefficients were measured using a standard protocol. [^123^I]-(**1**) displayed a Log P: 1.84 ± 0.06 and Log D: 1.57 ± 0.14, indicative of a lipophillic species possessing aromatic functionality as for the naphthyl group present. [^123^I]-(**3**) and [^123^I]-(**5**) were more polar with Log P: 1.14 ± 0.07 and Log D: 0.99 ± 0.06, and Log P: 1.18 ± 0.08 and Log D: 0.98 ± 0.07 respectively. [^123^I]-(**6**) displays a Log P: 1.57 ± 0.11 and Log D: 1.23 ± 0.21.

*In vitro* stability experiments were conducted in PBS, and in serum complete medium (10% FBS). At regular intervals over a 24 h period HPLC analysis was performed to evaluate complex stability. Incubation of [^123^I]-(**1**) and [^123^I]-(**6**) gave a single peak by radio-HPLC at all time points with an integrated area of >98% and a retention time that correlated with **1** and **6** respectively. No signs of deiodination or decomposition were observed in PBS buffer. In contrast, HPLC analysis of [^123^I]-(**3**) showed signs of complex decomposition with a loss of radio-iodide label within 4 h incubation in PBS. After 24 h in PBS, only 48% of intact compound remained. The only peaks in the radiotrace corresponded to intact complex and a peak with the same retention time as free iodide, suggesting direct loss of iodide, possibly as a result of a radical anion pathway mediated by the oxidation of the flavonoid species in solution to give reactive radical intermediates [61], [62]. The instability of [^123^I]-(**3**) and [^123^I]-(**5**) over extended incubation periods rendered these compounds unsuitable for further *in vitro* development. Similar complex instability was seen for [^123^I]-(**5**) in PBS, with only 53% intact compound remaining after 24 h respectively. In keeping with their lipophillic nature both **1** and **6** after incubation in serum containing medium, remained localized to the protein pellet after protein precipitation. Sephadex size exclusion separation of the solutions reinforced that all activity was associated with large protein biomolecules, with the radioactive fraction eluting in early solvent volumes. Free iodide controls eluted later suggesting that there is no loss of free iodide after 24 h incubation of **1** or **6** in serum containing medium.

### 2.4 In vitro evaluation

Cell uptake studies were performed in the MDA-MB-435 melanoma cell line as a telomerase expressing cell line. Internalization studies with [^123^I]-(**1**) showed limited cell uptake, with <0.1% of the radiolabeled drug internalized over a 24 h incubation period, despite favorable partition coefficient data. Repeat internalization studies with alternative cancer cell lines including MDA-231/H2N breast and U2OS osteosarcoma cancer cell lines all showed low cell uptake. No loss in survival was subsequently seen on incubation with the MDA-MB-435 cell line as determined by colony formation, and as such attention was focused thereafter on [^123^I]-(**3**) and the iodinated MST-312 compound.

After 24 h incubation, the iodinated MST-312 compound, [^123^I]-(**6**), displayed uptake in MDA-MB-435 cells, with 15% of the total incubated activity being cell-associated after 24 h, of which 60% was shown to be localized within the nucleus.

**Fig. 3:**
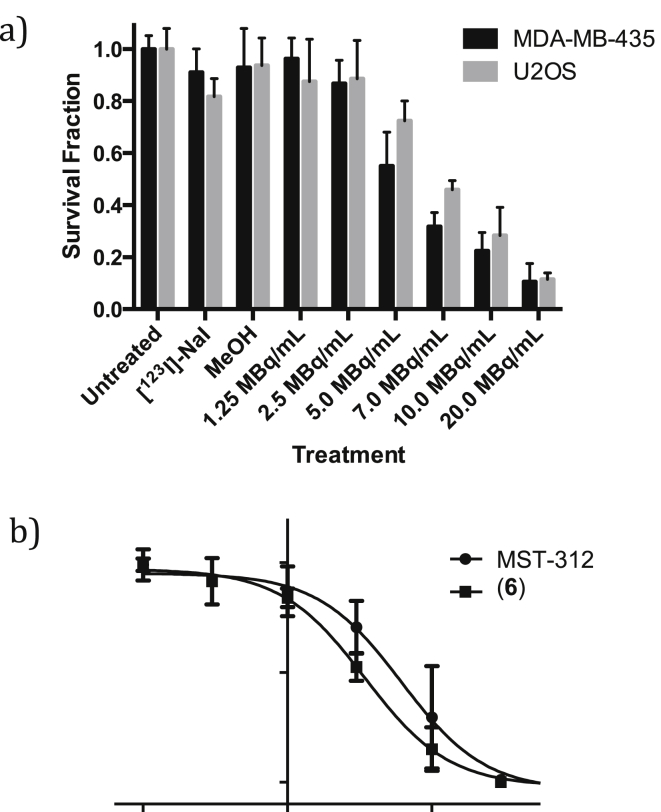
a) Clonogenic survival data for MDA-MB-435 and U2OS cell lines after 24 h treatment with increasing activity concentrations of [^123^I]-(**6**); b) Clonogenic survival data for MDA-MB-435 cell line after 24 h treatment with increasing activity concentrations of (**6**) or MST-312.

The effects of combined irradiation with telomerase inhibition have so far only been investigated using external beam sources. To date no approach has pursued the use of intrinsically targeted small molecule radiotherapeutics to study if radiosensitization effects can be achieved, by retention of a radioactive probe in cancer cells that over-express telomerase. Localization of a radionuclide in proximity to the enzyme active-site may further enhance catalytic inhibition, following radiolytic disruption. Binding to the active-site of this intranuclear enzyme is also likely to promote the retention of small molecule inhibitors in the nuclei (of cancer cells), potentiating effects mediated via radioactive decay. Given the nuclear uptake and telomerase inhibition of **6**, clonogenic studies were performed with increasing radioactivity concentrations of the radiolabeled [^123^I]-(**6**) species. Concentrations between 0 and 20 MBq/mL were incubated with the MDA-MB-435 cell line for either 4 or 24 h before being plated out at a concentration of 1000 cells/mL in DME medium and left to form colonies over a 10 day period. Reduced survival with increasing concentration of radiolabeled drug was observed, with lower surviving fraction (SF) after incubation for 24 h (Fig. 3a). Control clonogenic assays with MST-312 and non-radioactive **6** run in parallel show that for the radiolabeled species even at the highest radioactivity concentrations used (20 MBq/mL), the concentration of compound present (0.18 μM, based on specific activity measurements) was not sufficient to illicit a significant clonogenic effect (survival fraction: 0.97). As such the low SF observed is as a direct result of a response to radiation-induced cell damage (Fig. 3b).

For comparison with a telomerase negative cell line, the U2OS osteosarcoma cell line was studied in parallel to the MDA-MB-435 cell line (Fig. 3). Comparable uptake and retention to the MDA-MB-435 cell line was seen in the U2OS cell line. Clonogenic studies (n = 8) in U2OS and MDA-MB-435 cells lines run in parallel show that although at the highest activity-concentrations comparable loss in survival is observed for both cell lines (SF at 20 MBq/mL; 0.11 vs 0.11 for MDA-MB-435 vs U2OS, P = 0.76), at lower activity concentrations the SF for the MDA-MB-435 cell line is lower than that for U2OS (SF at 5 MBq/mL: 0.55 vs 0.72 for MDA-MB-435 vs U2OS, *P* = 0.004; SF at 7 MBq/mL: 0.32 vs 0.46 for MDA-MB-435 vs U2OS, *P* = 0.001), which it is hypothesised may result from the presence of telomerase facilitating nuclear retention of the iodinated MST-312 species.

**Fig. 4:**
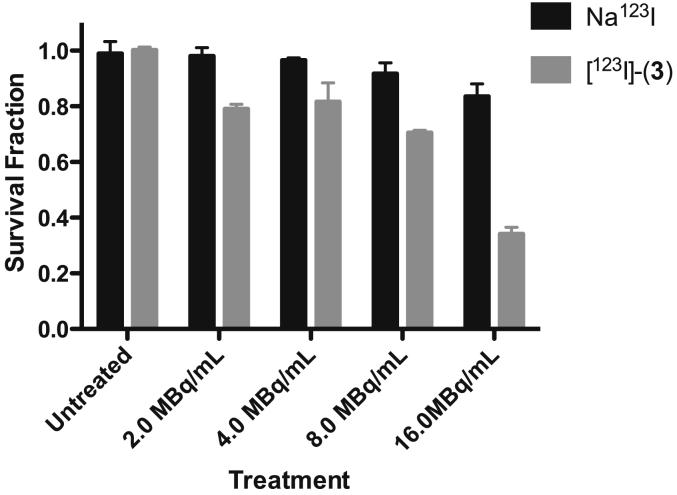
Clonogenic survival data for MDA-MB-435 cell line after 24 h treatment with increasing activity concentrations of [^123^I]-(**3**) or Na^123^I.

Cell studies with [^123^I]-(**3**) in the MDA-MB-435 cell line showed uptake of 2.9% of the total activity to be cell associated after 24 h. Clonogenic studies between 0 and 16 MBq/mL of [^123^I]-(**3**) with MDA-MB-435 cell line for 24 h showed statistically significantly reduced survival with increasing concentration of radiolabelled drug compared to free iodide alone (Fig. 4). Cell survival was reduced to 36 ± 2% following incubation with 16 MBq/mL of [^123^I]-(**3**), compared to 84 ± 4% with the Na^123^I control, suggesting that the combination of telomerase inhibition and irradiation is cytotoxic. However, given the instability of [^123^I]-(**3**) (and [^123^I]-(**5**)) over extended incubation periods such class of compounds were deemed not suitable for further development.

Excluding the germ line, telomerase expression in stem-cell compartments is rarely sufficient to maintain telomere length, with shortening observed with increasing age [63]. Though anti-telomerase therapy could possibly affect stem cells of renewal tissues (such as crypt cells of the intestine, basal cells of the skin, and certain hematopoietic cells of the blood) [64], the telomeres of such cells are generally much longer than cancer cell telomeres [5], [65], [66], [67]. This differential telomerase expression in cancer versus stem cells provides a means to deliver the telomerase-targeting radiotherapeutic [^123^I]-(**6**) to neoplastic cells, with minimal damage to normal stem-cell populations.

## 3 Conclusion

To investigate the possible use of telomerase as a target for molecularly targeted radiotherapy, we synthesized and characterized a panel of radioiodinated analogues of three known telomerase inhibitors: BIBR-1532, a flavonoid (2-(3,4-dihydroxyphenyl)-7,8-dihydroxy-4H-chromen-4-one) and MST-312. The radioiodinated derivatives all retained telomerase inhibitory capacity either in cell-free (lysate) or in whole cell experiments. ^123^I-BIBR-1532 internalized into cancer cells to only a modest extent. ^123^I-labeled flavonoid was markedly cytotoxic in clonogenic assays but was moderately unstable in serum. ^123^I-MST-312, however, showed uptake in two cancer cell lines, was stable and elicited radioactivity concentration-dependent cancer cell death. The reduction in cell survival was greater in a telomerase positive versus telomerase negative cell line. Further studies will look at a greater range of telomerase positive and negative cell lines to establish the broad applicability of (**6**) as a targeted radiotherapy agent against telomerase-positive cancers.

## 4 Experimental section

### 4.1 General methods

All reactions were carried out in oven-dried glassware under a nitrogen atmosphere. Nitrogen gas was dried by passage through silica gel. All solvents were dried according to standard procedures. All reagents were purchased from Aldrich and used without further purification. Column chromatography was performed on silica gel 60 M (mesh 230–400) Macherey-Nagel. ^1^H and ^13^C NMR spectra were recorded on a Bruker DPX400 (400 MHz) spectrometer or Bruker AVC500 (500 MHz) spectrometer at 298 K and referenced to residual non-deuterated solvent peaks. Mass spectrometry was performed using a Bruker Micromass LCT time-of-flight mass spectrometer under conditions of electrospray ionization (ESI-MS). Accurate masses are reported to four decimal places using tetraoctylammonium bromide (466.5352 Da) as an internal reference. HPLC characterization (analytical HPLC) of compounds was performed using a Waters C-18 column (4.6 × 250 mm) with UV/Vis detection at *λ*_obs_ = 254 nm with a 1.0 mL/min gradient elution method; Method A (Solvent A: acetonitrile with 0.1% TFA *v/v*, Solvent B: water with 0.1% TFA *v/v*): start 50% A, gradient over 7 min reaching 80% A, then gradient until 13 min reaching 95% A, reverse gradient till 14 min reaching 50% A, then hold to 15 min at 50% A, Method B (Solvent A: acetonitrile with 0.1% TFA *v/v*, Solvent B: water with 0.1% TFA *v/v*): start 5% A, gradient over 15 min reaching 95% A, hold to 16 min at 95% A, reverse gradient till 18 min reaching 5% A, then hold to 20 min at 5% A.

Elemental analyses were performed by Mr. S. Boyer, at London Metropolitan University.

No-carrier added radiochemical sodium iodide I-123 was supplied by GE Healthcare. Radio-HPLC was carried out under identical conditions to analytical HPLC, on an Agilent 1200 series module equipped with a LabLogic Gamma-Ram-4 radiodetector.

Compounds (*E*)-3-(naphthalen-2-yl)but-2-enoic acid [68], 4-iodo-2-aminobenzoate [69], 3-Iodo-4,5-dimethoxybenzoic acid [70] and 1-iodo-3,5-diaminobenzene [71] were prepared as previously described. All data was in accord with literature values. Full experimental data and spectra for all compounds prepared in this work may be found in the Supporting Information.

#### 4.1.1 I-BIBR-1532 compound data

##### 4.1.1.1 (E)-Methyl 5-iodo-2-(3-(naphthalen-2-yl)but-2-enamido)benzoate (1b)

(*E*)-3-(Naphthalen-2-yl)but-2-enoic acid (1.00 g, 4.71 mmol) was suspended in dry dichloromethane (15 mL) in a flame dried flask under an argon atmosphere and cooled to 0 °C. Oxalyl chloride (0.848 g, 0.447 mL, 9.42 mmol) and a catalytic amount of DMF (2 drops) was added to the suspension and allowed to warm to room temperature. The solution was stirred at room temperature for 5 h after which all volatiles were removed under reduced pressure. The crude product was redissolved in dry dichloromethane (15 mL) and solvent removed under reduced pressure three times before drying under high vac. The crude product was redissolved in dry THF (20 mL) in a flame-dried flask under an argon atmosphere. To this was added 5-iodo-2-aminobenzoate (1.86 g, 7.07 mmol), pyridine (1.12 g, 1.14 mL, 14.1 mmol) and a catalytic amount of DMAP (0.01 g, 0.082 mmol). After stirring at 60 °C for 16 h, the reaction was allowed to cool to room temperature, poured onto 1 N HCl and extracted into diethyl ether. The combined organic layers were dried over anhydrous magnesium sulphate and concentrated under vacuum to give the crude product. Purification by flash chromatography (8% methanol in dichloromethane) afforded the analytically pure product. (Yield: 1.60 g, 3.39 mmol, 72%). ^**1**^**H NMR** (CDCl_3_, 500 MHz, 20 °C): δ = 11.18 (s, 1H, N*H*), 8.70 (d, *J* = 9.1 Hz, 1H, Ar*H*), 8.36 (d, *J* = 2.2 Hz, 1H, Ar*H*), 7.98 (d, *J* = 1.3 Hz, 1H, Np*H*), 7.89 (m, 3H, Ar*H* + Np*H*), 7.83 (d, *J* = 2.2 Hz, 1H, Np*H*), 7.64 (dd, *J* = 8.5, 1.9 Hz, 1H, Np*H*), 7.53 (m, 2H, Np*H*), 6.37 (q, *J* = 0.9 Hz, 1H, COC*H*), 3.95 (s, 3H, COOC*H*_3_), 2.77 (d, *J* = 1.0 Hz, 3H, CC*H*_3_). ^**13**^**C NMR** (CDCl_3_, 125 MHz, 20 °C): δ = 167.6, 165.3, 154.0, 143.1, 141.7, 139.6, 139.3, 133.4, 133.1, 128.5, 128.2, 127.6, 126.6, 126.5, 125.8, 124.0, 122.2, 120.8, 116.5, 84.7, 52.6, 18.0. **ESMS** calcd for C_22_H_18_INNaO_3_ [M+Na]^+^: 494.0224, found 494.0221. **HPLC** (method A): R_t_ = 14.14 min.

##### 4.1.1.2 (E)-5-Iodo-2-(3-(naphthalen-2-yl)but-2-enamido)benzoic acid (1)

LiOH (27.8 mg, 0.663 mmol, 1 M aq. solution) was added to a solution of (*E*)-methyl 5-iodo-2-(3-(naphthalen-2-yl)but-2-enamido)benzoate (0.250 g, 0.530 mmol) in THF/H_2_O (5:1, 10 mL) and stirred at room temperature. After 12 h, the reaction was acidified to pH 4 using 1 M aq. oxalic acid solution and extracted with ethyl acetate. The combined organic layers were dried over anhydrous magnesium sulphate and concentrated under vacuum to give the final product. (Yield: 0.226 g, 0.493 mmol, 93%). ^**1**^**H NMR** (*d*_*6*_-DMSO, 500 MHz, 20 °C): δ = 13.87 (br s, 1H, COO*H*), 11.16 (s, 1H, N*H*), 8.38 (d, *J* = 8.8 Hz, 1H, Ar*H*), 8.23 (d, *J* = 2.2 Hz, 1H, Ar*H*), 8.14 (d, *J* = 1.3 Hz, 1H, Np*H*), 7.99 (m, 1H, Np*H*), 7.92 (m, 3H, Ar*H* + Np*H*), 7.75 (dd, *J* = 8.8, 1.9 Hz, 1H, Np*H*), 7.54 (m, 2H, Np*H*), 6.51 (d, *J* = 0.9 Hz, 1H, COC*H*), 2.66 (d, *J* = 1.1 Hz, 3H, CC*H*_3_). ^**13**^**C NMR** (*d*_*6*_-DMSO, 125 MHz, 20 °C): δ = 168.0, 164.5, 151.7, 142.0, 140.4, 139.0, 138.8, 133.0, 132.8, 128.5, 128.1, 127.4, 126.8, 126.6, 125.7, 123.9, 122.5, 120.8, 119.2, 85.9, 17.2. **ESMS** calcd for C_21_H_16_INNaO_3_ [M+Na]^+^: 480.0067, found 480.0071. **HPLC** (method A): R_t_ = 9.89 min. **Elemental analysis** for C_21_H_16_INO_3_, calc: C 55.2%, H 3.5%, N 3.1%, found: C 55.3%, H 3.6%, N 2.9%.

##### 4.1.1.3 Methyl (E)-2-(3-(naphthalen-2-yl)but-2-enamido)-5-(trimethylstannyl)benzoate (1c)

To a solution of (*E*)-methyl 5-iodo-2-(3-(naphthalen-2-yl)but-2-enamido)benzoate (0.100 g, 0.212 mmol) in degassed anhydrous 1,4-dioxane (5.00 mL) was added hexamethyldistannane (0.167 g, 106 μL, 0.509 mmol) and *bis*-(triphenylphosphine)-palladium(II)-dichloride (1.50 mg, 2.17 μmol), and the solution stirred at 60 °C for 1.5 h under argon. The solution was cooled to room temperature and filtered through a plug of celite with dichloromethane washing. The solvent was removed in vacuo and purification by flash chromatography on neutral silica (2% methanol in dichloromethane) afforded the analytically pure product as a white solid. (Yield: 69.0 mg, 0.136 mmol, 64%). ^**1**^**H NMR** (CD_2_Cl_2_, 500 MHz, 20 °C): δ = 11.20 (s, 1H, *H*9), 8.81 (d, *J* = 8.2 Hz, 1H, *H*7), 8.18 (d, *J* = 1.2 Hz (84%), with Sn satellites (*J*^2^_Sn-H_ = 47 and 44 Hz (16%)), 1H, *H*4), 8.01 (d, *J* = 1.6 Hz, 1H, *H*15), 7.84–7.95 (m, 3H, *H*17 + 20+22), 7.71 (dd, *J* = 8.1, 1.3 Hz (84%), with Sn satellites (*J*^2^_Sn-H_ = 48 Hz (16%)), 1H, *H*6), 7.68 (dd, *J* = 8.8, 1.8 Hz, 1H, *H*23), 7.53 (m, 2H, *H*18 + 19), 6.41 (d, *J* = 1.3 Hz, 1H, *H*11), 3.93 (s, 3H, *H*1), 2.75 (d, *J* = 1.3 Hz, 3H, *H*13), 0.34 (s (84%), with Sn satellites (*J*^2^_Sn-H_ = 54 and 56 Hz (16%)), 9H, Sn(C*H*_3_)_3_). ^**13**^**C NMR** (CD_2_Cl_2_, 125 MHz, 20 °C): δ = 169.6, 165.7, 153.2, 142.5, 142.4, 140.3, 138.6, 136.2, 134.0, 133.8, 129.0, 128.6, 128.1, 127.1, 127.0, 126.3, 124.6, 121.8, 120.0, 115.2, 52.8, 18.1, −9.2 (with Sn satellites; −7.8, −7.9, −10.6, −10.7), Sn(*C*H_3_)_3._ **ESMS** calcd for C_25_H_27_NNaO_3_Sn [M+Na]^+^: 532.0910, found 532.0899. **HPLC** (method B): R_t_ = 14.24 min.

##### 4.1.1.4 (E)-2-(3-(Naphthalen-2-yl)but-2-enamido)-5-(trimethylstannyl)benzoic acid (1d)

LiOH (4.71 mg, 0.197 mmol, 1 M aq. solution) was added to a solution of (*E*)-2-(3-(naphthalen-2-yl)but-2-enamido)-5-(trimethylstannyl)benzoate (0.050 g, 0.098 mmol) in THF/H_2_O (5:1, 10 mL) and stirred at room temperature. After 12 h, the reaction was acidified to pH 4 using 1 M aq. oxalic acid solution and extracted with ethyl acetate. The combined organic layers were dried over anhydrous magnesium sulphate and concentrated under vacuum to give the final product. (Yield: 42.3 mg, 0.086 mmol, 87%). ^**1**^**H NMR** (*d*_*6*_-DMSO, 500 MHz, 20 °C): δ = 13.52 (br s, 1H, *H*1), 11.21 (s, 1H, *H*9), 8.51 (d, *J* = 8.2 Hz, 1H, *H*7), 8.11 (d, *J* = 1.4 Hz, 1H, *H*15), 8.08 (d, *J* = 1.2 Hz (84%), with Sn satellites (*J*^2^_Sn-H_ = 46 and 43 Hz (16%)), 1H, *H*4), 7.92–8.02 (m, 3H, *H*17 + 20+22), 7.79 (dd, *J* = 8.7, 1.6 Hz, 1H, *H*23), 7.72 (dd, *J* = 8.2, 1.3 Hz (84%), with Sn satellites (*J*^2^_Sn-H_ = 47 and 44 Hz (16%)), 1H, *H*6), 7.56 (m, 2H, *H*18 + 19), 6.53 (d, *J* = 1.2 Hz, 1H, *H*11), 2.67 (d, *J* = 0.7 Hz, 3H, *H*13), 0.31 (s (84%), with Sn satellites (*J*^2^_Sn-H_ = 53 and 56 Hz (16%)), 9H, Sn(C*H*_3_)_3_). ^**13**^**C NMR** (*d*_*6*_-DMSO, 125 MHz, 20 °C): δ = 170.1, 164.4, 150.6, 140.8, 140.7, 138.9, 138.8, 133.0, 132.8, 131.5, 128.4, 128.1, 127.5, 126.7, 126.6, 125.6, 124.0, 122.4, 121.4, 119.4, 17.3, -9.3 (with Sn satellites; −7.6, −7.7, −10.4, −10.5), Sn(*C*H_3_)_3._ **ESMS** calcd for C_24_H_25_NNaO_3_Sn [M+Na]^+^: 518.0753, found 518.0744. **HPLC** (method B): R_t_ = 11.97 min. **Elemental analysis** for C_24_H_25_NO_3_Sn, calc: C 58.3%, H 5.1%, N 2.8%, found: C 57.9%, H 5.2%, N 2.9%.

#### 4.1.2 I-FLAV compound data

##### 4.1.2.1 1-(5-Iodo-2-hydroxy-3,4-dimethoxyphenyl) ethanone (3a)

To a solution of 3,4-dimethoxy-2-hydroxyacetophenone (0.855 g, 4.36 mmol) in acetonitrile (10 mL) was added *p*-toluenesulphonic acid (0.750 g, 4.36 mmol) and the mixture stirred for 15 min. *N*-Iodosuccinimide (0.980 g, 4.36 mmol) was added and the mixture stirred for 40 h at room temperature. The reaction was diluted with dichloromethane and washed with aq. Na_2_S_2_O_4_, aq. NaHCO_3_ and water. The combined organic layers were dried over anhydrous magnesium sulphate and concentrated under vacuum to give the crude product. Purification by flash chromatography (dichloromethane) afforded the analytically pure product as a white solid. (Yield: 0.955 g, 2.97 mmol, 68%). ^**1**^**H NMR** (CDCl_3_, 400 MHz, 20 °C): δ = 12.58 (s, 1H, O*H*), 7.90 (s, 1H, Ar*H*), 4.03 (s, 3H, OC*H*_3_), 3.90 (s, 3H, OC*H*_3_), 2.59 (s, 3H, COC*H*_3_).^**13**^**C NMR** (CDCl_3_, 100 MHz, 20 °C): δ = 202.7, 158.3, 157.8, 140.7, 134.9, 118.6, 78.2, 61.1, 60.9, 26.7. **ESMS** calcd for C_10_H_11_INaO_4_ [M+Na]^+^: 344.9594, found 344.9597. **IR:** ν = 2938, 1637, 1445, 1409, 1358, 1313, 1254, 1069, 1019, 753 cm^−1^.

##### 4.1.2.2 6-Acetyl-4-iodo-2,3-dimethoxyphenyl-3,4-dimethoxybenzoate (3b)

To a solution of 1-(5-Iodo-2-hydroxy-3,4-dimethoxyphenyl) ethanone (0.975 g, 3.027 mmol) in anhydrous pyridine (5 mL) under argon, was added 3,4-dimethoxybenzoyl chloride (1.82 g, 9.08 mmol) over a period of 15 min. The mixture was stirred for 2 h at room temperature and then acidified with 2 N HCl, extracted with ethyl acetate and washed with water. The combined organic layers were dried over anhydrous magnesium sulphate and concentrated under vacuum to give the crude product. Purification by flash chromatography (30% ethyl acetate in hexane) afforded the analytically pure product as a white solid. (Yield: 1.40 g, 2.88 mmol, 95%). ^**1**^**H NMR** (CD_2_Cl_2_, 500 MHz, 20 °C): δ = 8.04 (s, 1H, Ar*H*), 7.86 (dd, 1H, *J* = 8.5, 2.0 Hz, Ar*H*), 7.65 (d, 1H, *J* = 2.0 Hz, Ar*H*), 7.00 (d, 1H, *J* = 8.6 Hz, Ar*H*), 3.95 (s, 3H, OC*H*_3_), 3.94 (s, 3H, OC*H*_3_), 3.91 (s, 3H, OC*H*_3_), 3.83 (s, 3H, OC*H*_3_), 2.47 (s, 3H, COC*H*_3_). ^**13**^**C NMR** (CD_2_Cl_2_, 125 MHz, 20 °C): δ = 195.4, 164.5, 157.4, 154.7, 149.7, 146.2, 145.8, 134.5, 130.1, 125.2, 121.4, 113.1, 111.3, 88.9, 61.6, 61.3, 56.6, 56.5, 30.4. **ESMS** calcd for C_19_H_19_INaO_7_ [M+Na]^+^: 509.0068, found 509.0067. **IR:** ν = 2941, 1734, 1687, 1598, 1516, 1452, 1401, 1355, 1272, 1209, 1172, 1135, 1076, 1021, 752 cm^−1^.

##### 4.1.2.3 1-(5-Iodo-2-hydroxy-3,4-dimethoxyphenyl)-3-(3,4-dimethoxyphenyl)-1,3-propanedione (3c)

To a solution of 6-acetyl-4-iodo-2,3-dimethoxyphenyl-3,4-dimethoxybenzoate (1.18 g, 2.44 mmol) in anhydrous pyridine (5 mL), stirred at 50 °C, powdered potassium hydroxide (0.209 g, 3.73 mmol) was added. After 1 h, the reaction mixture was cooled, acidified with 2 N HCl, extracted with ethyl acetate and washed with water. The combined organic layers were dried over anhydrous magnesium sulphate and concentrated under vacuum to give the crude product. Purification by flash chromatography (30% ethyl acetate in hexane) afforded the analytically pure product as a yellow solid. NMR analysis in CD_2_Cl_2_ identified isomers in a 4:1 equilibrium of keto enol:1,3-diketone. (Yield: 1.04 g, 2.14 mmol, 88%). ^**1**^**H NMR** (CD_2_Cl_2_, 500 MHz, 20 °C): δ = 15.67 (s, 0.8H, COCHCO*H*′), 12.41 (s, 0.8H, O*H*′), 12.25 (s, 0.2H, O*H*), 7.95 (s, 1H, Ar*H*), 7.60 (m, 1H, Ar*H*), 7.51 (d, 0.2H, *J* = 1.9 Hz, Ar*H*), 7.45 (d, 0.8H, *J* = 2.2 Hz, Ar*H*′) 6.95 (d, 0.8H, *J* = 8.5 Hz, Ar*H*′), 6.93 (d, 0.2H, *J* = 8.5 Hz, Ar*H*), 6.66 (s, 0.8H, COC*H*′COH), 4.54 (s, 0.4H, COC*H*_2_CO), 4.02 (s, 0.6H, OC*H*_3_), 4.00 (s, 2.4H, OC*H′*_3_), 3.92 (s, 2.4H, OC*H′*_3_), 3.917 (s, 2.4H, *H* OC*H′*_3_), 3.914 (s, 0.6H, OC*H*_3_), 3.90 (s, 2.4H, OC*H′*_3_), 3.89 (s, 0.6H, OC*H*_3_), 3.87 (s, 0.6H, OC*H*_3_). ^**13**^**C NMR** (CD_2_Cl_2_, 125 MHz, 20 °C): δ = 199.7, 193.2, 192.2, 178.8, 159.4, 158.8, 157.8, 154.9, 154.0, 149.9, 149.8, 141.6, 141.1, 135.9, 133.0, 126.7, 126.2, 124.3, 121.7, 119.0, 118.4, 111.5, 111.0, 110.8, 110.2, 91.8, 79.11, 79.07, 61.7, 61.6, 61.4, 61.3, 56.60, 56.56, 56.5, 56.4, 50.1. **ESMS** calcd for C_19_H_19_INaO_7_ [M+Na]^+^: 509.0068, found 509.0073. **IR:** ν = 2936, 1597, 1574, 1513, 1446, 1404, 1323, 1241, 1143, 1020, 754 cm^−1^.

##### 4.1.2.4 3-Iodo-2-(3,4-dimethoxyphenyl)-7,8-dimethoxy-4H-chromen-4-one (3d)

A suspension of 1-(5-Iodo-2-hydroxy-3,4-dimethoxyphenyl)-3-(3,4-dimethoxyphenyl)-1,3-propanedione (0.850 g, 1.75 mmol) and sodium acetate (1.70 g, 2.07 mmol) in glacial acetic acid (10 mL) was refluxed for 2 h. After cooling, the suspension was concentrated to minimum volume under reduced pressure, extracted with ethyl acetate and washed with aq. NaHCO_3_ and water. The combined organic layers were dried over anhydrous magnesium sulphate and concentrated under vacuum to give the product as a white solid (Yield: 0.583 g, 1.25 mmol, 71%). ^**1**^**H NMR** (CDCl_3_, 500 MHz, 20 °C): δ = 8.11 (s, 1H, Ar*H-*5), 7.67 (dd, 1H, *J* = 8.5, 2.2 Hz, Ar*H-*2′), 7.56 (d, *J* = 2.2 Hz, 1H, Ar*H-*6′), 7.16 (d, 1H, *J* = 8.7 Hz, Ar*H-*3′), 7.04 (s, 1H, Ar*H-*3), 4.06 (s, 3H, OC*H*_3_), 3.98 (s, 3H,OC*H*_3_), 3.88 (s, 3H, OC*H*_3_), 3.86 (s, 3H, OC*H*_3_). ^**13**^**C NMR** (CDCl_3_, 125 MHz, 20 °C): δ = 175.3, 162.5, 155.5, 152.0, 150.5, 149.0, 140.9, 128.4, 123.1, 121.8, 119.7, 111.8, 109.1, 105.6, 89.0, 61.9, 61.2, 55.72, 55.66. **ESMS** calcd for C_19_H_17_INaO_6_ [M+Na]^+^: 490.9962, found 490.9966. **IR:** ν = 1650, 1515, 1429, 1363, 1328, 1272, 1259, 1224, 1144, 1077, 1026, 730 cm^−1^. **HPLC** (method B): R_t_ = 13.48 min.

##### 4.1.2.5 3-Iodo-2-(3,4-dihydroxyphenyl)-7,8-dihydroxy-4H-chromen-4-one (3)

To a portion of 3-iodo-2-(3,4-dimethoxyphenyl)-7,8-dimethoxy-4*H*-chromen-4-one (0.500 g, 1.07 mmol) dissolved in anhydrous dichloromethane (20 mL) and cooled to 0 °C, was added slowly a 1 M solution of BBr_3_ in dichloromethane (10.7 mL, 10.7 mmol). The solution was stirred at room temperature for 12 h and then diluted with iced water and the pH adjusted to 6 with aq. NaHCO_3_. The mixture was extracted with ethyl acetate, and the organic layer separated and washed with water and brine. The combined organic layers were dried over anhydrous magnesium sulphate and concentrated under vacuum to afford the analytically pure product as a yellow solid. (Yield: 0.183 g, 0.444 mmol, 42%). ^**1**^**H NMR** (*d*_*6*_-DMSO, 500 MHz, 20 °C): δ = 10.43 (br. s, 4H, CO*H*), 7.84 (s, 1H, Ar*H-*5), 7.55 (m, 2H, Ar*H-*2′ + 6′), 6.90 (d, 1H, *J* = 8.8 Hz, Ar*H-*3′), 6.61 (s, 1H, Ar*H-*3). ^**13**^**C NMR** (*d*_*6*_-DMSO, 125 MHz, 20 °C): δ = 175.4, 163.0, 150.4, 149.3, 146.9, 145.6, 132.6, 124.3, 122.1, 119.2, 118.3, 115.7, 113.9, 104.1, 83.1. **ESMS** calcd for C_15_H_16_IO_6_ [M − H]^-^: 410.9371, found 410.9373. **IR:** ν = 1615, 1593, 1534, 1463, 1403, 1286, 1196, 1171, 1126, 1032 cm^−1^. **HPLC** (method B): R_t_ = 8.01 min. **Elemental analysis** for C_15_H_9_IO_6_, calc: C 43.7%, H 2.2%, found: C 43.8%, H 2.4%.

##### 4.1.2.6 3-Iodo-2-(3,4-diacetoxyphenyl)-7,8-diacetoxy-4H-chromen-4-one (3f)

To a solution of 3-iodo-2-(3,4-dihydroxyphenyl)-7,8-dihydroxy-4*H*-chromen-4-one (0.150 g, 0.364 mmol) in degassed anhydrous pyridine (5.00 mL) was added acetic anhydride (5.00 mL) and the reaction stirred at 45 °C for 20 h. The solution was then diluted with iced water and stirred for a further 1 h at 0 °C. The white precipitate formed was collected under vacuum filtration, washed with ice cold water and further dried under high vacuum, before purification by flash chromatography (10% ethyl acetate in dichloromethane) afforded the analytically pure product as a white solid. (Yield: 0.142 g, 0.245 mmol, 67%). ^**1**^**H NMR** (*d*_*6*_-DMSO, 500 MHz, 20 °C): δ = 8.37 (s, 1H, Ar*H-*5), 7.91 (ap. dq, *J* = 4.6, 2.3 Hz, 2H, Ar*H-*2′ + 6′), 7.52 (d, 1H, Ar*H-*3′), 7.16 (s, 1H, Ar*H-3*), 2.51 (s, 3H, COC*H*_3_), 2.46 (s, 3H, COC*H*_3_), 2.332 (s, 3H, COC*H*_3_), 2.330 (s, 3H, COC*H*_3_). ^**13**^**C NMR** (*d*_*6*_-DMSO, 125 MHz, 20 °C): δ = 174.8, 168.2, 168.0, 167.3, 167.1, 161.0, 148.5, 147.3, 144.9, 142.5, 132.1, 131.3, 129.0, 124.78, 124.68, 123.4, 121.8, 107.9, 89.2, 20.5, 20.4, 19.8. **ESMS** calcd for C_23_H_17_INaO_10_ [M+Na]^+^: 602.9759, found 602.9768. **IR:** ν = 1779, 1654, 1428, 1361, 1196, 1119, 1074, 1018 cm^−1^. **HPLC** (method B): R_t_ = 12.58 min.

##### 4.1.2.7 2-(3,4-Diacetoxyphenyl)-7,8-diacetoxy-6-(trimethylstannyl)-4H-chromen-4-one (3g)

To a solution of 3-iodo-2-(3,4-diacetoxyphenyl)-7,8-diacetoxy-4*H*-chromen-4-one (0.100 g, 0.172 mmol) in degassed anhydrous 1,4-dioxane (3.00 mL) was added hexamethyldistannane (0.141 g, 0.090 mL, 0.431 mmol) and *bis*-(triphenylphosphine)-palladium(II)-dichloride (1.21 mg, 1.72 μmol), and the solution stirred at 60 °C for 90 min under argon. The solution was cooled to room temperature and filtered through a plug of celite with dichloromethane washing. The solvent was removed in vacuo and purification by flash chromatography on neutral silica (10% ethyl acetate in dichloromethane) afforded the analytically pure product as a white solid. (Yield: 61.7 mg, 0.100 mmol, 58%).^**1**^**H NMR** (CD_2_Cl_2_, 500 MHz, 20 °C): δ = 8.14 (s (84%), with Sn satellites (*J*^2^_Sn-H_ = 42 and 44 Hz (16%)), 1H, Ar*H-*5), 7.70 (dd, *J* = 8.5, 2.2 Hz, 1H, Ar*H-*2′), 7.67 (d, *J* = 2.2 Hz, 1H, Ar*H-*6′), 7.36 (d, *J* = 8.5 Hz, 1H, Ar*H-*3′), 6.72 (s, 1H, Ar*H-*3), 2.41 (s, 3H, COC*H*_3_), 2.35 (s, 3H, COC*H*_3_), 2.32 (s, 3H, COC*H*_3_), 2.32 (s, 3H, COC*H*_3_), 0.38 (s (84%), with Sn satellites (*J*^2^_Sn-H_ = 55 and 57 Hz (16%)), 9H, Sn(C*H*_3_)_3_). ^**13**^**C NMR** (CD_2_Cl_2_, 125 MHz, 20 °C): δ = 177.3, 168.5, 168.4, 168.3, 167.6, 162.0, 151.6, 150.2, 145.3, 143.3, 134.3, 131.8, 130.8, 130.7, 124.94, 124.86, 123.1, 122.2, 109.1, 21.04, 21.02, 20.97, 20.7, -8.6 (with Sn satellites; −7.1, −7.2, −10.0, −10.1). **ESMS** calcd for C_26_H_26_NaO_10_Sn [M+Na]^+^: 641.0445, found 641.0434. **IR:** ν = 1776, 1650, 1434, 1357, 1195, 1158, 1119, 1072, 1014 cm^−1^. **HPLC** (method B): R_t_ = 14.01 min.

#### 4.1.3 I-MST-312 compound data

##### 4.1.3.1 *N,N*′-(5-Iodo-1,3-phenylene)bis(2,3-dimethoxybenzamide)

To a solution of 2,3-dimethoxybenzoic acid (0.804 g, 4.40 mmol) in anhydrous dichloromethane (20 mL) under argon, was added 25 μL anhydrous dimethylformamide. The solution was cooled to 0 °C and oxalyl chloride (740 μL, 1.11 g, 8.80 mmol) added dropwise with stirring. The solution was allowed to warm to room temperature and stirred for a further 24 h. The solvent was removed under reduced pressure and the residue washed three times with fresh anhydrous dichloromethane, followed by drying under vacuum for 1 h to yield the crude 2,3-5-dimethoxybenzoyl chloride. To a solution of 1-iodo-3,5-diaminobenzene (0.468 g, 2.00 mmol) in anhydrous pyridine (10 mL) under argon, was added dropwise the crude 2,3-dimethoxybenzoyl chloride in dichloromethane (5 mL) over a period of 15 min. The mixture was then stirred for 2 h at room temperature, and then acidified with 2 N HCl, extracted with ethyl acetate and washed with water. The combined organic layers were dried over anhydrous magnesium sulphate and concentrated under vacuum to give the crude product. Purification by flash chromatography (30% ethyl acetate in hexane) afforded the analytically pure product. (Yield: 0.922 g, 1.64 mmol, 82%). ^**1**^**H NMR** (CD_2_Cl_2_, 400 MHz, 20 °C): δ = 10.07 (s, 2H, N*H*), 8.12 (t, *J* = 1.8 Hz, 1H, Ar*H*), 7.88 (d, *J* = 2.0 Hz, 2H, Ar*H*), 7.72 (dd, *J* = 8.1, 1.5 Hz, 2H, Ar*H*), 7.21 (t, *J* = 7.9 Hz, 2H, Ar*H*), 7.14 (dd, *J* = 8.2, 1.6 Hz, 2H, Ar*H*), 4.01 (s, 6H, 2 × OC*H*_3_), 3.92 (s, 6H, 2 × OC*H*_3_).^**13**^**C NMR** (CD_2_Cl_2_, 100 MHz, 20 °C): δ = 163.5, 153.3, 148.0, 140.6, 126.8, 125.1, 124.8, 123.1, 116.7, 111.5, 94.3, 62.2, 56.7. **ESMS** calcd for C_24_H_23_IN_2_NaO_6_ [M+Na]^+^: 585.0493, found 585.0496. **HPLC:** *R*_*t*_ = 14.82 min (Method B). **IR:** ν = 3319, 1674, 1593, 1578, 1530, 1471, 1435, 1315, 1264, 1057, 984, 741 cm^−1^.

##### 4.1.3.2 *N,N*′-(5-Iodo-1,3-phenylene)bis(2,3-dihydroxybenzamide)

To a solution of *N,N*′-(5-iodo-1,3-phenylene)bis(2,3-dimethoxybenzamide) (0.100 g, 0.178 mmol) in anhydrous dichloromethane (15 mL), cooled to 0 °C, was added, dropwise, a 1 M solution of BBr_3_ in dichloromethane (1.78 mL, 1.78 mmol). The reaction was stirred for 12 h at room temperature and then diluted with iced water (100 mL). The pH of the suspension was adjusted to 6 with 5% NaHCO_3_, extracted with ethyl acetate and washed with water and brine. The combined organic layers were dried over anhydrous magnesium sulphate and concentrated under vacuum to afford the analytically pure product as a yellow solid. (Yield: 41.7 mg, 82.3 μmol, 46%). ^**1**^**H NMR** (*d*_*6*_-DMSO, 400 MHz, 20 °C): δ = 11.37 (s, 2H, O*H*), 10.43 (s, 2H, O*H*), 9.50 (br. s., 2H, N*H*), 8.13 (t, *J* = 1.8 Hz, 1H, Ar*H*), 7.94 (d, *J* = 1.8 Hz, 2H, Ar*H*), 7.41 (dd, *J* = 8.0, 1.1 Hz, 2H, Ar*H*), 6.99 (dd, *J* = 7.8, 1.1 Hz, 2H, Ar*H*), 6.78 (t, *J* = 8.0 Hz, 2H, Ar*H*). ^**13**^**C NMR** (*d*_*6*_-DMSO, 100 MHz, 20 °C): δ = 168.4, 148.8, 147.1, 140.6, 125.7, 119.9, 119.5, 118.3, 113.5, 94.9. **ESMS** calcd for C_20_H_15_IN_2_NaO_6_ [M+Na]^+^: 528.9867, found 528.9856. **HPLC:** *R*_*t*_ = 11.49 min (Method B). **IR:** ν = 3360, 1648, 1589, 1533, 1462, 1436, 1333, 1256, 1184, 849, 730 cm^−1^.

##### 4.1.3.3 *N,N*′-(5-(Trimethylstannyl)-1,3-phenylene)bis(2,3-diacetoxybenzamide)

To a solution of 2,3-bis(acetyloxy)benzoic acid (0.263 g, 1.1 mmol) in anhydrous dichloromethane (20 mL) under argon, was added 25 μL anhydrous dimethylformamide. The solution was cooled to 0 °C and oxalyl chloride (185 μL, 0.278 g, 2.2 mmol) added dropwise with stirring. The solution was allowed to warm to room temperature and stirred for a further 24 h. The solvent was removed under reduced pressure and the residue washed three times with fresh anhydrous dichloromethane, followed by drying under vacuum for 1 h to yield the crude 2,3- bis(acetyloxy)benzoyl chloride. To a solution of 1-iodo-3,5-diaminobenzene (0.117 g, 0.50 mmol) in anhydrous pyridine (10 mL) under argon, was added dropwise the crude 2,3- bis(acetyloxy)benzoyl chloride in dichloromethane (5 mL) over a period of 15 min. The mixture was then stirred for 2 h at room temperature, and then acidified with 2 N HCl, extracted with ethyl acetate and washed with water. The combined organic layers were dried over anhydrous magnesium sulphate and concentrated under vacuum to give the crude product. The crude product was semi-purified by flash chromatography (30% ethyl acetate in hexane) to afford a mixture of products. To a solution of this crude mixture in degassed anhydrous 1,4-dioxane (5 mL) is added hexamethyldistannane (184 μL, 292 mg, 0.891 mmol) and *bis*-(triphenylphosphine)-palladium(II)-dichloride (2.60 mg, 3.71 μmol), and the solution stirred at 75 °C for 12 h. The solution was cooled to room temperature, filtered through a plug of celite and washed with dichloromethane. The solvent was removed in vacuo and purification by flash chromatography (10% ethyl acetate in dichloromethane) afforded the analytically pure product. (Yield: 0.092 g, 0.13 mmol, 26%). ^**1**^**H NMR** (CD_2_Cl_2_, 500 MHz, 20 °C): δ = 8.03 (s, 3H, 3 × Ar*H*), 7.68 (dd, *J* = 7.6, 1.6 Hz, 2H, Ar*H*), 7.43 (d (84%), *J* = 1.6 Hz with Sn satellites (*J*^2^_Sn-H_ = 54 and 56 Hz (16%), 2H, Ar*H*), 7.39 (t, *J* = 7.8 Hz, 2H, Ar*H*), 7.36 (dd, *J* = 1.8 Hz, 2H, Ar*H*), 2.32 (s, 6H, COC*H*_3_), 2.31 (s, 6H, COC*H*_3_), 0.33 (s (84%) with Sn satellites (*J*^2^_Sn-H_ = 54 and 56 Hz (16%)), 9H, Sn(C*H*_3_)_3_).^**13**^**C NMR** (CD_2_Cl_2_, 125 MHz, 20 °C): δ = 168.8, 168.6, 163.4, 145.4, 143.7, 140.6, 138.5, 131.3, 127.4, 127.2, 126.8, 123.6, 112.3, 21.0, 20.9, -9.2 (Sn(*C*H_3_)_3_). **ESMS** calcd for C_31_H_32_N_2_NaO_10_Sn [M+Na]^+^: 735.0971, found 735.0982. **IR:** ν = 3341, 1773, 1676, 1590, 1535, 1460, 1425, 1371, 1203, 1018 cm^−1^. **HPLC:** *R*_*t*_ = 12.46 min (Method B).

### 4.2 Radiolabeling

#### 4.2.1 I-BIBR-1532 (1)

To a solution of the trimethyltin analogue of BIBR-1532 (**1d**), (20 μg, 0.040 μmol) in ethanol (20 μL), was added [^123^I]-NaI (150 MBq, 5 μL) and Chloramine T trihydrate (20 μL, 1 mg/mL solution in water). After 15 min at room temperature, Na_2_S_2_O_5_ (1 mg, 6.32 μmol in 20 μL H_2_O), NaHCO_3_ (2 mg, 23.81 μmol in 20 mL H_2_O), and 350 μL of HPLC mobile phase were added. The resulting mixture was purified by semi-preparative HPLC (Method A). The radiolabeled compound (retention time R_t_: 9.89 min) was collected, loaded onto a pre-conditioned C18 Seppak cartridge (pre-eluted with 2 mL ethanol followed with 5 mL H_2_O) washed with 5 ml H_2_O until pH of eluent reached pH 7. [^123^I]-(**1**) was then eluted with 0.5 mL methanol, collected and concentrated to dryness under a stream of nitrogen gas, and resuspended in DMSO (4 μL). The radioiodinated tracer was then formulated in 100 μL PBS or DME medium (final DMSO volume 4%) for *in vitro* evaluation. End of synthesis radiochemical yields, purities and specific activity measurements, represent the mean of at least five independent measurements (±SD). For HPLC determination of the specific activity of radiolabeled compound a calibration curve (linear regression analysis of mass from solutions of known compound concentration versus peak area of UV absorption) was plotted.

#### 4.2.2 I-Flavonoids (3)–(5)

To a solution of the trimethyltin flavonoid analogue (**3g**) (20 μg, 0.049 μmol) in ethanol (20 μL), was added [^123^I]-NaI (150 MBq, 5 μL) and H_2_O_2_:acetic acid (1:3) (20 μL). After 15 min at room temperature, Na_2_S_2_O_5_ (1 mg, 6.32 μmol in 20 μL H_2_O), NaHCO_3_ (2 mg, 23.81 μmol in 20 mL H_2_O), and 3.00 mL of deionised H_2_O were added. The solution was loaded onto a pre-conditioned C18 Seppak cartridge (pre-eluted with 2 mL ethanol followed with 5 mL H_2_O) washed with 5 mL H_2_O, eluted with 2 mL methanol, collected and concentrated to minimal volume under a stream of nitrogen gas. 3 M HCl (50 μL) was added to the radiolabeled species in a sealed glass vial and heated at 80 °C for 30 min. The reaction was cooled to room temperature and subsequently purified by semipreparative HPLC (Method B). The radiolabeled compound (retention time R_t_: 8.01 min) was collected, loaded onto a pre-conditioned C18 Seppak cartridge (pre-eluted with 2 mL ethanol followed with 5 mL H_2_O) washed with 5 ml H_2_O until pH of eluent reached pH 7. The [^123^I]-(**3**) was then eluted with 2 mL methanol, collected and concentrated to dryness under a stream of nitrogen gas, and resuspended in DMSO (5 μL). The radioiodinated tracer was then formulated in 100 μL PBS or DME medium (final DMSO volume 5%) for *in vitro* evaluation. Flavonoids (**4g** and **5g**) were radiolabeled in an identical manner.

#### 4.2.3 I-MST-312: (6f)

I-MST-312 (retention time R_t_: 11.35 min) was radiolabeled in an identical manner to the flavonoid analogues.

### 4.3 Octanol/PBS distribution coefficient (LogD) and octanol/water partition coefficient (LogP)

The distribution coefficients (log D) and partition coefficients (log P) of the radiolabeled compounds were determined by addition of a sample of the HPLC-purified radiolabeled compound (500 kBq) to equal amounts of water or PBS (pH 7.4) and octanol (500 μl each). The vials were vortexed vigorously for 1 min. To achieve phase separation, the vials were centrifuged (2500 rpm) for 6 min. The radioactivity concentration was determined in a defined volume of each layer measured in a γ-counter. The distribution coefficient was expressed as the ratio of counts per minute (cpm) measured in the octanol phase to the cpm measured in either the water or PBS phase. The results represent the mean of three independent measurements (±SD), each performed in quintuplicate.

### 4.4 Telomerase Repeat Amplification Protocol (TRAP) assay

The TRAP assay was set-up as recommended, with the following modifications: the PCR cycle was optimized to 30 °C, 30 min; followed by 36 cycles of 94 °C/30 s; 53.5 °C/30 s; 72 °C/60 s; and a final extension step of 72 °C/3 min. PCR reactions contained two units of Jumpstart-Taq, 2 μL of CHAPS-extracted cell lysate (see ESI for lysate preparation) containing 200 ng of protein, and 5x Reaction buffer; and were made-up to 50 μL with nuclease-free water. TSR8 template positive (0.2 amoles) and heat-inactivated-lysate (80 °C, 10 min) negative controls were included in each experiment. For inhibition experiments, inhibitor or control was added to a total volume of 55.5 μL, to the telomerase extension step (30 °C, 30 min). Samples were analysed by addition of 40 μL of each reaction to 160 μL of read buffer in 96-well format using a fluorescence plate reader. Signals attributed to the addition of telomeric repeats to a substrate primer (TS), and so telomerase activity, are represented by fluorescein emission following excitation (485/535 nm). The assay also includes an internal PCR control template and primer labeled with sulforhodamine (585/620 nm). Data processing involves subtraction of background signals, obtained from extract-free and Taq polymerase-free controls, producing telomerase (ΔFl) and internal control (ΔSulf) signals respectively. Telomerase signals are then normalized to internal PCR control (ΔFl/ΔSulf) to allow direct comparison of relative activities. The determined telomerase activity was normalized to untreated control. Data were fitted using fixed-slope non-linear regression and compared using an exact-sum-of-squares F-test in GraphPad Prism 5.0.

### 4.5 Complex stability

The stability of radiolabeled molecules was determined in PBS, and in serum containing medium. HPLC purified radiolabeled agents (50 MBq, 25 μL) were mixed with either 475 μL of PBS or 475 μL of medium and incubated at 37 °C. Aliquots of the PBS samples were taken for analysis at defined time points after incubation (0, 1, 4, 8 and 24 h) and analysed directly by radio-HPLC. Aliquots (50 μL) of the medium samples were taken for analysis at defined time points after incubation (0, 1, 4, 8 and 24 h), diluted with 450 μL MeOH to precipitate out proteins and the remaining solution analysed by radio-HPLC.

### 4.6 Internalization assay

Cells were harvested into an eppendorf at a density of 10^5^ cells in 250 μL of medium. Cells were treated for the indicated time in a total volume of 500 μL of medium, with 0.2 MBq of compound. Cell treatment was staggered to allow time-points to be processed together. Following incubation, cells were pelleted by centrifugation (500 × *g*, 5 min) and the medium was aspirated and retained. Cells were then washed twice with 500 μL and 250 μL PBS, and the washes combined with the medium to constitute the free-fraction. Free-fractions were diluted 10-fold to facilitate counting in a γ-counter. Cells were re-pelleted and cell membranes washed using 500 μL glycine (pH 2.5) and incubated at 4 °C for 6 min before washing with 500 μL PBS. Following re-pelleting, internalized and nuclear fractions were isolated using a Thermo Scientific Subcellular Protein Fractionation Kit for Cultured Cells. Fractions were counted in a Wizard Automatic gamma-counter (Perkin-Elmer 2480). Where appropriate data were fitted with a two-phase association model and compared using an exact-sum-of-squares F-test in GraphPad Prism 5.0.

### 4.7 Clonogenic assay

Cells were harvested and added to an eppendorf at a density of 10^5^ cells in 250 μL of medium. Compound was added to the desired concentration or activity to a total volume of 500 μL of medium. Solvent or cold compound (to a concentration equivalent to the highest activity) treated cells were included as a control. Cells were then incubated for 24 h before plating in six-well plates at a density sufficient to give >50 colonies for counting. Untreated cells were typically seeded at 750 cells/well. Colonies were grown for >7 days, washed in PBS and stained with 1% methylene blue (in 50% methanol). Excess stain was washed-off with water. Colonies containing >50 cells were counted. The surviving fraction (SF) was calculated using the plating efficiency (PE) of untreated cells.

## Funding

This work was supported by Cancer Research UK (C5255/A15935) and the Medical Research Council (MC_PC_12004).

## Figures and Tables

**Table 1:** Telomerase inhibition data for compounds **1-6**.

Compound	IC_50_ (μM)
BIBR-1532	11.57
**1**	30.09
**2**	28.08
Flavonoid	0.74
**3**	1.65
**4**	N/D
**5**	1.73
MST-312	0.23
**6**	1.58

N/D: Not determined.

## Appendix A Supplementary data

The Supporting Information contains full experimental analysis for all compounds with accompanying ^1^H NMR, ^13^C NMR and HRMS. TRAP protocol detail and inhibition curves are given along with cell/nuclear uptake curves and clonogenic survival curves. This material is available free of charge via the Internet at http://pubs.acs.org.
