# Supplementary material for: Targeting telomerase with radiolabeled inhibitors
Source: Eur J Med Chem. 2017 Jan 5;125:117–29. doi: 10.1016/j.ejmech.2016.09.028 (PMC5154340; doi:10.1016/j.ejmech.2016.09.028)
Supplement: Supplementary file 1 [file mmc1.docx]

**Supplementary Information**

Targeting Telomerase with Radiolabeled Inhibitors

Philip A. Waghorn,^a^ Mark R. Jackson,^a^ Veronique Gouverneur^b^ and Katherine A.Vallis^a^*

^a^ Gray Institute for Radiation Oncology and Biology, University of Oxford, Oxford, UK.

^b^ Chemistry Research Laboratory, University of Oxford 12 Mansfield Road, Oxford, UK.

**2-5**: Full Experimental detail

**6-61**: ^1^H and ^13^C NMR and HRMS

**27**: TRAP protocol

**28-29**: TRAP inhibitor curves

**30**: Cell/nuclear uptake curves

**31-32**: Clonogenic survival plots

**1. Full Experimental details:**

**1.1. 3-Iodo-4,5-Dimethoxybenzaldehyde**

To a solution of 3-iodo-4-hydroxy-5-methoxybenzaldehyde (1.05 g, 3.78 mmol) in CH_2_Cl_2_ (50.0 mL) was added aqueous NaOH solution (1.93 g in 30.0 mL of water) and *^t^*butylammonium iodide (2.09 g, 5.66 mmol) and stirred until clear. Methyl iodide (2.80 mL, 45.8 mmol) was added to the reaction mixture and stirred for 12 h at room temperature. The reaction was quenched with 6N HCl, and the organic product was extracted with CH_2_Cl_2_, washed with brine, dried, and concentrated to give a solid. The solid was chromatographed (pentane:dichloromethane, 1:1) to give a white solid. (Yield: 957 mg, 3.28 mmol, 87%). **^1^H NMR** (*d_1_*-CDCl_3_, 400 MHz, 20°C): δ = 9.81 (s, 1H, C(O)*H*), 7.83 (d, *J* = 1.8 Hz, 1H, Ar*H*), 7.39 (d, *J* = 1.8 Hz, 1H, Ar*H*), 3.91 (m, 6H, 2x OC*H*_3_). **^13^C NMR** (*d_1_*-CDCl_3_, 100 MHz, 20°C): δ = 189.7, 154.1, 153.0, 134.6, 133.9, 111.1, 92.1, 60.6, 56.1. **ESMS** calcd for C_9_H_10_IO_3_ [M+H]^+^ : 292.9669, found 292.9675.

**1.2. 3-Iodo-4,5-Dimethoxybenzoic acid**

3-Iodo-4,5-dimethoxybenzaldehyde (913 mg, 3.12 mmol) and 2-methyl-2-butene (3.10 mL) were dissolved in *tert*-butanol (10.0 mL), and a solution of 80% sodium chlorite (564 mg, 6.24 mmol) and monobasic sodium phosphate (487 mg 4.06 mmol) in water (7.00 mL) was added dropwise. The mixture was stirred for 4 h at room temperature. The solvent was removed in vacuo and the residue was diluted with water (40.0 mL). The pH of the solution was adjusted to 10 with 1 M aqueous NaOH, the aqueous phase was extracted with ether (2×20.0 mL). The aqueous layers were acidified to pH 2 by dropwise addition of 3 M aqueous HCl and extracted with ethyl acetate (3×25.0 mL). The combined organic layers were dried over anhydrous MgSO_4_ and the solvent was removed in vacuo to give a white solid. (Yield: 915 mg, 2.97 mmol, 95%). **^1^H NMR** (*d_6_*-DMSO, 400 MHz, 20°C): δ = 13.14 (br. s., 1H, COO*H*), 7.88 (d, *J* = 2.0 Hz, 1H,Ar*H*), 7.53 (d, *J* = 1.8 Hz, 1H, Ar*H*), 3.86 (s, 3H, OC*H*_3_), 3.77 (s, 3H, OC*H*_3_). **^13^C NMR** (*d_6_*-DMSO, 100 MHz, 20°C): δ = 166.5, 152.9, 152.8, 132.1, 129.3, 114.6, 93.2, 60.9, 56.9. **ESMS** calcd for C_9_H_8_IO_4_ [M-H]^-^ : 306.9743, found 306.9750

**1.3. (*E*)-Methyl 4-iodo-2-(3-(naphthalen-2-yl)but-2-enamido)benzoate (2b)**

Procedure as for (**1b**) with (*E*)-3-(naphthalen-2-yl)but-2-enoic acid (1.00 g, 4.71 mmol), oxalyl chloride (0.848 g, 0.447 mL, 9.42 mmol), 4-iodo-2-aminobenzoate (1.86 g, 7.07 mmol), pyridine (1.12 g, 1.14 mL, 14.1 mmol) and a catalytic amount of DMAP (0.01 g, 0.082 mmol). Purification by flash chromatography (8% methanol in dichloromethane) afforded the analytically pure product. (Yield: 1.49 g, 3.16 mmol, 67 %).**^1^H NMR** (*d_1_*-CDCl_3_, 500 MHz, 20°C): δ =11.22 (s, 1H, N*H*), 9.36 (d, *J* = 1.9 Hz, 1H, Ar*H*), 7.98 (d, *J* = 1.3 Hz, 1H, Np*H*), 7.90 (m, 1H, Np*H*), 7.85 (m, 2H, Np*H*), 7.72 (d, *J* = 8.5 Hz, 1H, Ar*H*), 7.65 (dd, *J* = 8.5, 1.9 Hz, 1H, Np*H*), 7.52 (m, 2H, Np*H*), 7.46 (dd, *J* = 8.4, 1.7 Hz, 1H, Ar*H*), 6.37 (q, *J* = 0.9 Hz, 1H, COC*H*), 3.93 (s, 3H, COOC*H*_3_), 2.78 (d, *J* = 0.9 Hz, 3H, CC*H*_3_). **^13^C NMR** (*d_1_*-CDCl_3_, 125 MHz, 20°C): δ = 168.6, 165.2, 154.2, 142.4, 139.6, 133.4, 133.1, 131.7, 131.6, 129.0, 128.5, 128.2, 127.6, 126.6, 126.5, 125.9, 124.0, 120.6, 113.8, 102.6, 52.5, 18.0. **ESMS** calcd for C_22_H_18_INNaO_3_ [M+Na]^+^ : 494.0224, found 494.0218. **HPLC** (method A): R_t_ =14.18 min.

**1.4. (*E*)-4-iodo-2-(3-(naphthalen-2-yl)but-2-enamido)benzoic acid (2)**

Procedure as for (**1**) with LiOH (27.8 mg, 0.663 mmol, 1 M aq. Solution) and **(***E)*-methyl 4-iodo-2-(3-(naphthalen-2-yl)but-2-enamido)benzoate (0.250 g, 0.530 mmol). (Yield: 0.216 g, 0.472 mmol, 89 %). **^1^H NMR** (*d_6_*-DMSO, 500 MHz, 20°C): δ = 11.97 (br. s, 2H, *H*1+9), 9.05 (d, *J* = 2.1 Hz, 1H, *H*7), 8.16 (d, *J* = 1.5 Hz, 1H, *H*15), 8.00 (m, 1H, *H*17), 7.96 (m, 2H, *H*20+22), 7.76 (dd, *J* = 8.3, 1.9 Hz, 1H, *H*23), 7.74 (d, *J* = 8.4 Hz, 1H, *H*4), 7.56 (m, 2H, *H*18+19), 7.51 (dd, *J* = 8.5, 1.9 Hz, 1H, *H*5), 6.50 (q, *J* = 1.1 Hz, 1H, *H*11), 2.68 (d, *J* = 0.9 Hz, 3H, *H*13). **^13^C NMR** (*d_6_*-DMSO, 125 MHz, 20°C): δ = 169.5 (*C*2), 165.1 (*C*10), 152.0 (*C*12), 142.1 (*C*6), 139.3 (*C*8), 133.5 (*C*4), 133.3 (*C*14), 133.1 (*C*21), 131.0 (*C*16), 129.0 (*C*17), 128.6 (*C*20/22), 128.0 (*C*20/22), 127.6 (*C*19), 126.7 (*C*18), 126.5 (*C*15), 126.2 (*C*23), 124.4 (*C*7), 121.3 (*C*11), 116.8 (*C*3), 102.6 (*C*5), 17.6 (*C*13). **ESMS** calcd for C_21_H_15_INO_3_ [M-H]^-^ : 456.0102, found 456.0093. **HPLC** (method A): R_t_ = 9.97 min. **Elemental analysis** for C_21_H_16_INO_3_, calc: C 55.2 %, H 3.5 %, N 3.1 %, found: C 54.8 %, H 3.7 %, N 3.3 %.

**1.5. 2-(3,4-Dimethoxyphenyl)-7,8-dimethoxy-6-(trimethylstannyl)-4*H*-chromen-4-one (3e)**

To a solution of 3-iodo-2-(3,4-dimethoxyphenyl)-7,8-dimethoxy-4*H*-chromen-4-one (0.200 g, 0.427 mmol) in degassed anhydrous 1,4-dioxane (5 mL) was added hexamethyldistannane (0.336 g, 0.214 mL, 1.03 mmol) and bis-(triphenylphosphine)-palladium(II)-dichloride (3.00 mg, 4.27 µmol), and the solution stirred at 60 °C for 90 min. The solution was cooled to room temperature and filtered through a plug of celite with dichloromethane washing. The solvent was removed in vacuo and purification by flash chromatography (0-4% diethyl ether in dichloromethane) afforded the analytically pure product as a white solid. (Yield: 0.161 g, 0.317 mmol, 74 %). **^1^H NMR** (CD_2_Cl_2_, 500 MHz, 20°C): δ = 7.87 (s (84%), with Sn satellites (*J*^2^_Sn-H_ = 47 and 49 Hz (16%)), 1H, Ar*H-*5), 7.61 (dd, *J* = 8.5, 2.2 Hz, 1H, Ar*H-*2’), 7.45 (d, *J* = 2.2 Hz, 1H, Ar*H-*6’), 7.01 (d, *J* = 8.7 Hz, 1H, Ar*H-*3’), 6.68 (s, 1H, Ar*H-*3), 4.06 (s, 3H, OC*H*_3_), 4.05 (s, 3H, OC*H*_3_), 3.94 (s, 3H, OC*H*_3_), 3.92 (s, 3H, OC*H*_3_), 0.34 (s (84%), with Sn satellites (*J*^2^_Sn-H_ = 54 and 57 Hz (16%)), 9H, Sn(C*H*_3_)_3_).**^13^C NMR** (CD_2_Cl_2_, 125 MHz, 20°C): δ = 178.1, 163.1, 161.0, 152.8, 152.5, 150.0, 139.8, 134.0, 127.6, 124.8, 121.3, 120.2, 111.9, 109.4, 106.6, 62.0, 61.4, 56.5, 56.4, -8.6 (with Sn satellites; -7.3, -7.4, -10.1, -10.2). **ESMS** calcd for C_22_H_26_NaO_6_Sn [M+Na]^+^ : 529.0648, found 529.0641. **IR:** ν = 2945, 1634, 1518, 1359, 1332, 1265, 1223, 1141, 1076, 1038, 1017, 878, 736 cm^-1^. **HPLC** (method B): R_t_ = 15.80 min. **Elemental analysis** for C_22_H_26_O_6_Sn, calc: C 52.3 %, H 5.2 %, found: C 51.8 %, H 5.4 %.

**1.6. 6-Acetyl-2,3-dimethoxyphenyl 3-iodo-4,5-dimethoxybenzoate (5b)**

To a solution of 3-iodo-4,5-dimethoxybenzoic acid (0.588 g, 1.91 mmol) in anhydrous dichloromethane (25.0 mL) under argon, is added 25.0 µL anhydrous dimethylformamide, and the solution cooled to 0 ºC and oxalyl chloride (0.606 g, 410 µL, 4.78 mmol) added dropwise with stirring. The solution was allowed to warm to room temperature before being stirred for a further 24h. The solvent was removed under reduced pressure and the residue washed three times with fresh anhydrous dichloromethane, followed by drying under high vacuum for 1h to yield the crude 3-iodo-4,5-dimethoxybenzoyl chloride which is used in the next step without further work up. To a solution of 1-(2-hydroxy-3,4-dimethoxyphenyl)ethanone (0.250 g, 1.27 mmol) in anhydrous pyridine (5.00 mL) under argon, was added 3-iodo-4,5-dimethoxybenzoyl chloride in dichloromethane (5.00 mL) over a period of 15 min. The mixture was stirred for 2 h at room temperature and then acidified with 2 N HCl, extracted with ethyl acetate and washed with water. The combined organic layers were dried over anhydrous magnesium sulphate and concentrated under vacuum to give the crude product. Purification by flash chromatography (30% ethyl acetate in hexane) afforded the analytically pure product. (Yield: 0.543 g, 1.12 mmol, 88 %). **^1^H NMR** (*d_6_*-DMSO, 500 MHz, 20°C): δ = 8.09 (d, *J* = 1.8 Hz, 1H, Ar*H*), 7.78 (d, *J* = 8.8 Hz, 1H, Ar*H*), 7.69 (d, *J* = 1.8 Hz, 1H, Ar*H*), 7.14 (d, *J* = 8.8 Hz, 1H, Ar*H*), 3.94 (s, 3H, OC*H*_3_), 3.91 (s, 3H, OC*H*_3_), 3.84 (s, 3H, OC*H*_3_), 3.70 (s, 3H, OC*H*_3_), 2.46 ppm (s, 3H, COC*H*_3_). **^13^C NMR** (*d_6_*-DMSO, 125 MHz, 20°C): δ = 195.4, 162.5, 156.8, 153.0, 152.3, 143.2, 140.9, 131.9, 126.5, 126.3, 123.6, 114.2, 109.9, 92.8, 60.6, 60.2, 56.4, 56.2, 29.2. **ESMS** calcd for C_19_H_19_INaO_7_ [M+Na]^+^ : 509.0068, found 509.0060. **IR**: ν = 2939, 1744, 1677, 1594, 1506, 1454, 1367, 1264, 1203, 1171, 1092, 1021, 758 cm^-1^.

**1.7. 1-(2-Hydroxy-3,4-dimethoxyphenyl)-3-(3-iodo-4,5-dimethoxyphenyl)propane-1,3-dione (5c):**

Procedure as for (**3c**) with 6-acetyl-2,3-dimethoxyphenyl 3-iodo-4,5-dimethoxybenzoate (0.300 g, 0.617 mmol) in anhydrous pyridine (3.00 mL). Purification by flash chromatography (30 % ethyl acetate in hexane) afforded the analytically pure product as a yellow solid in a 4:1 enol:keto ratio. (Yield: 0.246 g, 0.506 mmol, 82%). **^1^H NMR** (CD_2_Cl_2_, 500 MHz, 20°C): δ = 15.46 (s, 0.8H, COCHCO*H*’), 12.10 (s, 0.8H, O*H*’), 12.06 (s, 0.2H, O*H*), 7.98 (d, *J* = 1.9 Hz, 0.2H, Ar*H*), 7.92 (d, *J* = 1.9 Hz, 0.8H, Ar*H*’), 7.59 (d, *J* = 9.1 Hz, 0.8H, Ar*H’*), 7.55 (d, *J* = 1.9 Hz, 0.2H, Ar*H*), 7.52 (d, *J* = 9.1 Hz, 0.2H, Ar*H*), 7.46 (d, *J* = 2.1 Hz, 0.8H, Ar*H*’), 6.69 (s, 0.8H, COC*H’*COH), 6.56 (d, *J* = 9.1 Hz, 0.8H, Ar*H*’), 6.54 (d, *J* = 9.1 Hz, 0.2H, Ar*H*), 4.53 (s, 0.4H, COC*H*_2_CO), 3.98 (s, 3H, OC*H’*_3_), 3.97 (s, 3H, OC*H’*_3_), 3.96 (s, 0.6H, OC*H*_3_), 3.94 (s, 0.6H, OC*H*_3_), 3.933 (s, 0.6H, OC*H*_3_), 3.925 (s, 3H, OC*H’*_3_), 3.87 (s, 3H, OC*H’*_3_), 3.85 (s, 0.6H, OC*H*_3_). **^13^C NMR** (CD_2_Cl_2_, 125 MHz, 20°C): δ = 199.3, 195.0, 192.0, 175.1, 159.8, 159.1, 157.9, 157.6, 154.2, 153.1, 153.0, 152.7, 137.4, 137.1, 134.2, 132.6, 131.9, 129.7, 128.0, 125.5, 115.5, 114.5, 113.0, 111.6, 104.0, 103.9, 92.8, 92.7, 92.4, 61.03, 61.01, 60.84, 60.82, 56.7-56.6 (4 peaks), 50.2. **ESMS** calcd for C_19_H_19_INaO_7_ [M+Na]^+^ : 509.0068, found 509.0060. **IR:** ν = 2936, 1600, 1554, 1449, 1422, 1263, 1145, 1088, 1016, 787 cm^-1^.

**1.8. 2-(3-Iodo-4,5-dimethoxyphenyl)-7,8-dimethoxy-4*H*-chromen-4-one (5d):**

Procedure as for (**3d**) with 1-(2-hydroxy-3,4-dimethoxyphenyl)-3-(3-iodo-4,5-dimethoxyphenyl)propane-1,3-dione (0.450 g, 0.925 mmol), sodium acetate (0.900 g, 10.97 mmol) and glacial acetic acid (15.0 mL). (Yield: 308 mg, 0.657 mmol, 71%). **^1^H NMR** (*d_1_*-CDCl_3_, 500 MHz, 20°C): δ = 8.16 (d, *J* = 1.9 Hz, 1H, Ar*H-*2’), 8.13 (d, *J* = 8.9 Hz, 1H, Ar*H-*5), 7.58 (d, *J* = 2.1 Hz, 1H, Ar*H-*6’), 7.48 (s, 1H, Ar*H-3*), 7.39 (d, *J* = 9.1 Hz, 1H, Ar*H-*6), 4.17 (s, 3H, OC*H*_3_), 4.15 (s, 3H, OC*H*_3_), 4.02 (s, 3H, OC*H*_3_), 4.00 (s, 3H, OC*H*_3_). **^13^C NMR** (*d_1_*-CDCl_3_, 125 MHz, 20°C): δ = 178.2, 168.1, 159.9, 153.8, 153.0, 151.4, 136.3, 130.9, 127.3, 122.1, 113.5, 113.4, 111.5, 102.6, 93.0, 62.2, 61.2, 57.1, 56.2. **ESMS** calcd for C_19_H_17_INaO_6_ [M+Na]^+^ : 490.9962, found 490.9949. **IR:** ν = 1651, 1600, 1484, 1416, 1369, 1289, 1208, 1101, 1040, 996, 734 cm^-1^. **HPLC** (method B): R_t_ = 13.49 min.

**1.9. 2-(3,4-Dihydroxy-5-iodophenyl)-7,8-dihydroxy-4*H*-chromen-4-one (5)**

Procedure as for (**3**) with 2-(3-iodo-4,5-dimethoxyphenyl)-7,8-dimethoxy-4H-chromen-4-one (0.150 g, 0.320 mmol), and 1 M solution of BBr_3_ in dichloromethane (3.20 mL, 3.20 mmol). (Yield: 71.3 mg, 0.173 mmol, 54%). **^1^H NMR** (*d_6_*-DMSO, 500 MHz, 20°C): δ = 10.35 (br. s., 2H, 2xO*H*), 10.04 (br. s., 1H, O*H*), 9.45 (br. s., 1H, O*H*), 7.95 (d, *J* = 2.2 Hz, 1H, Ar*H-*6’), 7.46 (d, *J* = 2.2 Hz, 1H, Ar*H-*2’), 7.38 (d, *J* = 8.5 Hz, 1H, Ar*H-*5), 6.94 (d, *J* = 8.5 Hz, 1H, Ar*H-*6), 6.60 (s, 1H, Ar*H-*3). **^13^C NMR** (*d_6_*-DMSO, 125 MHz, 20°C): δ = 176.7 (*C*4), 160.9 (*C*2), 150.3 (*C*7), 149.0 (*C*4’), 146.5 (*C*9), 144.6 (*C*5’), 133.2 (*C*8), 127.3 (*C*6’), 124.2 (*C*1’), 117.0 (*C*10), 115.0 (*C*5), 113.8 (*C*6), 112.9 (*C*2’), 104.6 (*C*3), 85.3 (*C*3’). **ESMS** calcd for C_15_H_19_INaO_6_ [M+Na]^+^ : 434.9336, found 434.9331. **IR:** ν = 1623, 1572, 1547, 1429, 1405, 1308, 1213, 1190, 1137, 1032, 1011 cm^-1^. **HPLC** (method B): R_t_ = 7.75 min. **Elemental analysis** for C_15_H_9_IO_6_, calc: C 43.7 %, H 2.2 %, found: C 43.6 %, H 2.1 %.

**1.10. 2-(3,4-Dimethoxy-5-(trimethylstannyl)phenyl)-7,8-dimethoxy-4*H*-chromen-4-one (5e)**

Procedure as for (**3e**) with 2-(3-iodo-4,5-dimethoxyphenyl)-7,8-dimethoxy-4H-chromen-4-one (0.150 g, 0.320 mmol), hexamethyldistannane (0.262 g, 0.167 mL, 0.801 mmol) and bis-(triphenylphosphine)-palladium(II)-dichloride (2.248 mg, 3.203 µmol). Purification by flash chromatography (0-4% diethyl ether in dichloromethane) afforded the analytically pure product. (Yield: 0.110 g, 0.218 mmol, 68.1 %). **^1^H NMR** (CD_2_Cl_2_, 500 MHz, 20°C): δ = 7.87 (d, *J* = 8.8 Hz, 1H, Ar*H-*5), 7.63 (d, *J* = 2.2 Hz (84%), with Sn satellites (*J*^2^_Sn-H_ = 45 and 47 Hz (16%)), 1H, Ar*H-*2’), 7.48 (d, *J* = 2.2 Hz, 1H, Ar*H-*6’), 7.06 (d, *J* = 8.9 Hz, 1H, Ar*H-*6), 6.68 (s, 1H, Ar*H-*3), 4.03 (s, 3H, OC*H*_3_), 3.99 (s, 3H, OC*H*_3_), 3.95 (s, 3H, OC*H*_3_), 3.91 (s, 3H, OC*H*_3_), 0.20 - 0.48 (s (84%), with Sn satellites (*J*^2^_Sn-H_ = 54 and 57 Hz (16%)), 9H, Sn(C*H*_3_)_3_). **^13^C NMR** (CD_2_Cl_2_, 125 MHz, 20°C): δ = 178.1, 163.5, 157.3, 156.7, 152.2, 151.2, 137.5, 137.2, 128.3, 126.5, 121.1, 119.2, 111.6, 110.4, 106.5, 61.9, 61.3, 57.0, 55.9, -8.7 (with Sn satellites; -7.4, -7.5, -10.2, -10.3). **ESMS** calcd for C_22_H_26_NaO_6_Sn [M+Na]^+^ : 529.0648, found 529.0640. **IR:** ν = 1638, 1597, 1509, 1461, 1406, 1361, 1286, 1206, 1150, 1100, 1062, 1041, 1004, 767 cm^-1^. **HPLC** (method B): R_t_ = 16.34 min.

**1.11. 5-(7,8-Diacetoxy-4-oxo-4H-chromen-2-yl)-3-iodo-1,2-phenylene diacetate (5f):**

Procedure as for (**3f**) with 2-(3,4-dihydroxy-5-iodophenyl)-7,8-dihydroxy-4H-chromen-4-one (0.250 g, 0.607 mmol). Flash chromatography (10% ethyl acetate in dichloromethane) afforded the analytically pure product as a white solid. (Yield: 0.278 g, 0.480 mmol, 79 %). **^1^H NMR** (CD_2_Cl_2_, 500 MHz, 20°C): δ = 8.16 (d, *J* = 2.2 Hz, 1H, Ar*H-*6’), 8.06 (d, *J* = 8.5 Hz, 1H, Ar*H-*5), 7.69 (d, *J* = 2.2 Hz, 1H, Ar*H-*2’), 7.27 (d, *J* = 8.7 Hz, 1H, Ar*H-*6), 6.70 (s, 1H, Ar*H-*3), 2.46 (s, 3H, COC*H*_3_), 2.39 (s, 3H, COC*H*_3_), 2.36 (s, 3H, COC*H*_3_), 2.31 (s, 3H, COC*H*_3_). **^13^C NMR** (CD_2_Cl_2_, 125 MHz, 20°C): δ = 176.9, 168.2, 167.8, 167.5, 160.5, 149.7, 147.5, 146.7, 143.6, 134.5, 132.3, 132.0, 123.5, 123.1, 122.4, 120.8, 109.2, 92.8, 21.1, 21.0, 20.6. **ESMS** calcd for C_23_H_17_INaO_10_ [M+Na]^+^ : 602.9759, found 602.9769. **IR:** ν = 1774, 1639, 1409, 1363, 1288, 1205, 1164, 1033 cm^-1^. **HPLC** (method B): R_t_ = 12.38 min

**1.12. 5-(7,8-Diacetoxy-4-oxo-4H-chromen-2-yl)-3-(trimethylstannyl)-1,2-phenylene diacetate (5g):**

Procedure as for (**3g**) with 5-(7,8-diacetoxy-4-oxo-4*H*-chromen-2-yl)-3-iodo-1,2-phenylene diacetate (0.100 g, 0. 172 mmol hexamethyldistannane (0.141 g, 89.3 µL, 0. 431 mmol) and bis-(triphenylphosphine)-palladium(II)-dichloride (6.05 mg, 8.617 µmol). Flash chromatography on neutral silica (1-7.5% diethyl ether in dichloromethane) afforded the analytically pure product as a white solid. (Yield: 0.037 g, 0.060 mmol, 34.9 %). **^1^H NMR** (CD_2_Cl_2_, 500 MHz, 20°C): δ = 8.07 (d, *J* = 8.8 Hz, 1H, Ar*H-*5), 7.77 (d, *J* = 2.2 Hz (84%), with Sn satellites (*J*^2^_Sn-H_ = 39 and 44 Hz (16%), 1H, Ar*H-*2’), 7.67 (d, *J* = 2.2 Hz, 1H, Ar*H-*6’), 7.27 (d, *J* = 8.6 Hz, 1H, Ar*H-*6), 6.74 (s, 1H, Ar*H-*3), 2.43 (s, 3H, COC*H*_3_), 2.35 (s, 3H, COC*H*_3_), 2.32 (s, 3H, COC*H*_3_), 2.29 (s, 3H, COC*H*_3_), 0.38 (s (84%), with Sn satellites (*J*^2^_Sn-H_ = 55 and 57 Hz (16%)), 9H, Sn(C*H*_3_)_3_). **^13^C NMR** (CD_2_Cl_2_, 125 MHz, 20°C): δ = 177.1, 168.7, 168.5, 168.3, 167.8, 162.8, 150.0, 149.9, 147.3, 142.7, 139.1, 132.4, 131.8, 130.7, 123.4, 123.2, 122.8, 120.6, 108.9, 21.2, 21.0, 20.9, 20.6, -8.6 (with Sn satellites; -7.1, -7.2, -10.0, -10.1). **ESMS** calcd for C_26_H_26_NaO_10_Sn [M+Na]^+^ : 641.0440, found 641.0449. **IR:** ν =1774, 1652, 1367, 1260, 1202, 1136, 1074, 1034, 1015, 796 cm^-1^. **HPLC** (method B): R_t_ = 14.18 min.

**1.13. 6-Acetyl-2,3-dimethoxyphenyl 2-iodo-4,5-dimethoxybenzoate (4b):**

Procedure as for (**3b**) with 2-iodo-4,5-dimethoxybenzoic acid (1.00 g, 3.25 mmol), oxalyl chloride (696 µL, 1.03 g, 8.11 mmol) and 1-(2-hydroxy-3,4-dimethoxyphenyl)ethanone (0.531 g, 2.70 mmol) in anhydrous pyridine (5 mL). Purification by flash chromatography (30 % ethyl acetate in hexane) afforded the analytically pure product. (Yield: 1.406 g, 2.892 mmol, 89.1 %). **^1^H NMR** (CD_2_Cl_2_, 400 MHz, 20°C): δ = 7.80 (s, 1H, Ar*H*), 7.67 (d, *J* = 8.8 Hz, 1H, Ar*H*), 7.48 (s, 1H, Ar*H*), 6.93 (d, *J* = 8.8 Hz, 1H, Ar*H*), 3.96 (s, 3H, OC*H*_3_), 3.91 (s, 3H, OC*H*_3_), 3.90 (s, 3H, OC*H*_3_), 3.84 (s, 3H, OC*H*_3_), 2.49 (s, 3H, COC*H*_3_). **^13^C NMR** (CD_2_Cl_2_, 100 MHz, 20°C): δ = 196.1, 163.9, 157.8, 153.3, 149.5, 144.5, 142.1, 126.5, 125.4, 125.0, 124.6, 115.3, 109.7, 85.9, 61.5, 56.8, 56.7, 56.5, 29.9. **ESMS** calcd for C_19_H_19_INaO_7_ [M+Na]^+^ : 509.0068, found 509.0060. **IR:** ν = 2940, 1738, 1678, 1596, 1500, 1453, 1402, 1357, 1279, 1213, 1182, 1156, 1097, 1034, 749 cm^-1^.

**1.14. 1-(2-Hydroxy-3,4-dimethoxyphenyl)-3-(2-iodo-4,5-dimethoxyphenyl)propane-1,3-dione (4c):**

Procedure as for (**3c**) with 6-acetyl-2,3-dimethoxyphenyl 2-iodo-4,5-dimethoxybenzoate (0.300 g, 0.617 mmol) and powdered potassium hydroxide (52.0, 3.00 g, 0.927 mmol). Purification by flash chromatography (5 % diethyl ether in dichloromethane) afforded the analytically pure product as a yellow solid in a 17:3 enol:keto ratio. (Yield: 0.273 g, 0.561 mmol, 90.9 %). **^1^H NMR** (CD_2_Cl_2_, 500 MHz, 20°C): δ = 15.07 (s, 1H, COCHCO*H*’), 12.14 (s, 1H, O*H*’), 12.10 (s, 0.15H, O*H*), 7.56 (d, *J* = 9.0 Hz, 0.15H, Ar*H*), 7.53 (d, *J* = 9.1 Hz, 1H, Ar*H*’), 7.38 (s, 0.15H, Ar*H*), 7.36 (s, 1H, Ar*H*’), 7.27 (s, 0.15H, Ar*H*), 7.10 (s, 1H, Ar*H*’), 6.58 (s, 1H, COC*H*’COH), 6.54 (m, Ar*H*’+Ar*H*), 4.57 (s, 0.3H, COC*H*_2_CO), 3.915 (s, 0.45H, OC*H*_3_), 3.912 (s, 3H, OC*H’*_3_), 3.869 (s, 3H, OC*H’*_3_), 3.866 (s, 0.45H, OC*H*_3_), 3.859 (s, 3H, OC*H’*_3_), 3.853 (s, 0.45H, OC*H*_3_), 3.833 (s, 3H, OC*H’*_3_), 3.805 (s, 0.45H, OC*H*_3_). **^13^C NMR** (CD_2_Cl_2_, 125 MHz, 20°C): δ = 199.0, 195.0, 194.6, 178.9, 159.8, 159.2, 157.9, 157.7, 152.7, 151.7, 149.9, 149.5, 137.3, 137.0, 133.6, 132.4, 128.2, 125.7, 124.5, 123.6, 115.5, 114.6, 114.0, 113.1, 104.1, 104.0, 98.1, 83.5, 82.8, 60.8, 56.8, 56.71, 56.7, 56.62, 56.61, 56.58, 52.56. **ESMS** calcd for C_19_H_19_INaO_7_ [M+Na]^+^ : 509.0068, found 509.0068. **IR:** ν = 2934, 1606, 1558, 1499, 1445, 1254, 1209, 1145, 1085, 1009, 784 cm^-1^

**1.15. 2-(2-Iodo-4,5-dimethoxyphenyl)-7,8-dimethoxy-4*H*-chromen-4-one (4d):**

Procedure as for (**3d**) with 1-(2-hydroxy-3,4-dimethoxyphenyl)-3-(2-iodo-4,5-dimethoxyphenyl)propane-1,3-dione (0.250 g, 0.514 mmol), and sodium acetate (0.500 g, 0.610 mmol) in glacial acetic acid (5 mL). (Yield: 0.175 g, 0.374 mmol, 73 %). **^1^H NMR** (*d_6_*-DMSO, 500 MHz, 20°C): δ = 7.82 (d, *J* = 8.8 Hz, 1H, Ar*H-*5), 7.48 (s, 1H, Ar*H-*3’), 7.32 (d, *J* = 9.0 Hz, 1H, Ar*H-*6), 7.27 (s, 1H, Ar*H-*6’), 6.41 (s, 1H, ArH-*3*), 3.97 (s, 3H, OC*H*_3_), 3.90 (s, 3H, OC*H*_3_), 3.85 (s, 3H, OC*H*_3_), 3.80 (s, 3H, OC*H*_3_). **^13^C NMR** (*d_6_*-DMSO, 125 MHz, 20°C): δ = 176.4, 165.5, 156.6, 150.8, 150.2, 148.7, 136.4, 129.9, 122.0, 120.2, 117.8, 114.1, 111.2, 111.0, 85.2, 61.5, 56.5, 56.1, 55.8. **ESMS** calcd for C_19_H_17_INaO_6_ [M+Na]^+^ : 490.9962, found 490.9959. **IR:** ν = 1645, 1603, 1510, 1366, 1291, 1258, 1213, 1175, 1103, 1024, 734 cm^-1^. **HPLC** (method B): R_t_ = 11.82 min.

**1.16. 2-(3,4-Dihydroxy-6-iodophenyl)-7,8-dihydroxy-4*H*-chromen-4-one (4)**

Procedure as for (**3**) with 2-(2-iodo-4,5-dimethoxyphenyl)-7,8-dimethoxy-4H-chromen-4-one (0.150 g, 0.320 mmol) and a 1 M solution of BBr_3_ in dichloromethane (3.20 mL, 3.20 mmol). (Yield: 0.061 g, 0.147 mmol, 46 %). **^1^H NMR** (*d_6_*-DMSO, 500 MHz, 20°C): δ = 10.36 (s, 1H, O*H*), 9.92 (s, 1H, O*H*), 9.64 (s, 1H, O*H*), 9.32 (s, 1H, O*H*), 7.41 (d, *J* = 8.7 Hz, 1H, Ar*H-*5), 7.32 (s, 1H, Ar*H-*3’), 7.04 (s, 1H, Ar*H-*6’), 6.96 (d, *J* = 8.5 Hz, 1H, Ar*H-*6), 6.23 (s, 1H, Ar*H-*3). **^13^C NMR** (*d_6_*-DMSO, 125 MHz, 20°C): δ = 176.6, 165.1, 150.3, 148.6, 146.9, 145.7, 133.3, 128.5, 126.0, 117.6, 116.9, 114.9, 114.0, 110.7, 83.1. **ESMS** calcd for C_15_H_9_INaO_6_ [M+Na]^+^ : 434.9336, found 434.9329. **IR:** ν = 1630, 1581, 1551, 1429, 1403, 1367, 1269, 1222, 1185, 1026, 1007 cm^-1^. **HPLC** (method A): R_t_ = 7.25 min. **Elemental analysis** for C_15_H_9_IO_6_, calc: C 43.7 %, H 2.2 %, found: C 43.5 %, H 2.4 %.

**1.17. 2-(4,5-Diacetoxy-2-iodophenyl)-4-oxo-4*H*-chromene-7,8-diyl diacetate (4f)**

Procedure as for (**3f**) with 2-(3,4-dihydroxy-6-iodophenyl)-7,8-dihydroxy-4H-chromen-4-one (0.200 g, 0.485 mmol). Purification by flash chromatography (10% ethyl acetate in dichloromethane) afforded the analytically pure product as a white solid. (Yield: 0.206 g, 0.355 mmol, 73 %). **^1^H NMR** (CD_2_Cl_2_, 500 MHz, 20°C): δ = 8.09 (d, *J* = 8.8 Hz, 1H, Ar*H-*5), 7.85 (s, 1H, Ar*H-*3’), 7.39 (s, 1H, Ar*H-*6’), 7.30 (d, *J* = 8.8 Hz, 1H, Ar*H-*6), 6.53 (s, 1H, Ar*H-*3), 2.37 (s, 3H, COC*H*_3_), 2.34 (s, 3H, COC*H*_3_), 2.31 (s, 3H, COC*H*_3_), 2.30 (s, 3H, COC*H*_3_).**^13^C NMR** (CD_2_Cl_2_, 125 MHz, 20°C): δ = 176.8, 168.20, 168.18, 168.1, 164.4, 150.1, 147.6, 144.6, 143.1, 136.1), 136.0, 132.4, 126.2, 123.4, 123.2, 120.9, 113.7, 90.7, 21.02, 20.95, 20.91, 20.8. **ESMS** calcd for C_23_H_17_INaO_10_ [M+Na]^+^ : 602.9759, found 602.9769. **IR:** ν = 1778, 1656, 1478, 1370, 1260, 1196, 1141, 1014 cm^-1^. **HPLC** (method B): R_t_ = 12.06 min.

**1.18. 2-(4,5-Diacetoxy-2-(trimethylstannyl)phenyl)-4-oxo-4H-chromene-7,8-diyl diacetate (4g)**

Procedure as for (**3g**) with 2-(4,5-diacetoxy-2-iodophenyl)-4-oxo-4H-chromene-7,8-diyl diacetate (0.075 g, 0. 129 mmol), hexamethyldistannane (0.106 g, 67.0 µL, 0. 323 mmol) and bis-(triphenylphosphine)-palladium(II)-dichloride (4.54 mg, 6.46 µmol). Purification by flash chromatography on neutral silica (1-7.5% diethyl ether in dichloromethane) afforded the analytically pure product as a white solid. (Yield: 46.1 mg, 0.075 mmol, 58 %). **^1^H NMR** (CD_2_Cl_2_, 500 MHz, 20°C): δ = 8.09 (d, *J* = 8.8 Hz, 1H, Ar*H-*5), 7.44 (s, (84%), with Sn satellites (*J*^2^_Sn-H_ = 39 and 44 Hz (16%)),1H, Ar*H-*6’), 7.42 (s, 1H, Ar*H-*3’), 7.30 (d, *J* = 8.8 Hz, 1H, Ar*H-*6), 6.39 (s, 1H, Ar*H-*3), 2.34 (s, 6H, COC*H*_3_), 2.32 (s, 3H, COC*H*_3_), 2.30 (s, 3H, COC*H*_3_), 0.33 ppm (s (84%), with Sn satellites (*J*^2^_Sn-H_ = 54 and 56 Hz (16%)), 9H, Sn(C*H*_3_)_3_). **^13^C NMR** (CD_2_Cl_2_, 125 MHz, 20°C): δ = 176.6, 168.7, 168.6, 168.2, 167.9, 166.4, 150.2, 147.4, 144.1, 143.0, 142.7, 139.0, 132.32, 132.29, 124.5, 123.5, 123.0, 120.8, 112.3, 21.05, 21.01, 20.7, -7.3. **ESMS** calcd for C_26_H_26_NaO_10_Sn [M+Na]^+^ : 641.0445, found 641.0436. **IR:** ν = 1775, 1655, 1368, 1260, 1192, 1162, 1137, 1074, 1036, 1013, 775 cm^-1^. **HPLC** (method B): R_t_ = 13.80 min

2. ^1^H and ^13^C NMR and HRMS

**2.1. (E)-5-iodo-2-(3-(naphthalen-2-yl)but-2-enamido)benzoic acid (1)**

**^1^H NMR** (*d_6_*-DMSO, 500 MHz, 20°C): δ = 13.87 (br s, 1H, *H*1), 11.16 (s, 1H, *H*9), 8.38 (d, *J* = 8.8 Hz, 1H, *H*7), 8.23 (d, *J* = 2.2 Hz, 1H, *H*4), 8.14 (d, *J* = 1.3 Hz, 1H, *H*15), 7.99 (m, 1H, *H*17), 7.92 (m, 3H, *H*6+20+22), 7.75 (dd, *J* = 8.8, 1.9 Hz, 1H, *H*23), 7.54 (m, 2H, *H*18+19), 6.51 (q, *J* = 0.9 Hz, 1H, *H*11), 2.66 (d, *J* = 1.1 Hz, 3H, *H*13). **^13^C NMR** (*d_6_*-DMSO, 125 MHz, 20°C): δ = 168.0 (*C*2), 164.5 (*C*10), 151.7 (*C*12), 142.0 (*C*6), 140.4 (*C*8), 139.0 (*C*4), 138.8 (*C*14), 133.0 (*C*21), 132.8 (*C*16), 128.5 (*C*17), 128.1 (*C*20/22), 127.4 (*C*20/22), 126.8 (*C*19), 126.6 (*C*18), 125.7 (*C*15), 123.9 (*C*23), 122.5 (*C*7), 120.8 (*C*11), 119.2 (*C*3), 85.9 (*C*5), 17.2 (*C*13). **ESMS** calcd for C_21_H_15_INO_3_ [M+Na]^+^ : 480.0067, found 480.0071. **HPLC** (method B): R_t_ = 9.89 min. **Elemental analysis** for C_21_H_16_INO_3_, calc: C 55.2 %, H 3.5 %, N 3.1 %, found: C 55.3 %, H 3.6 %, N 2.9 %.


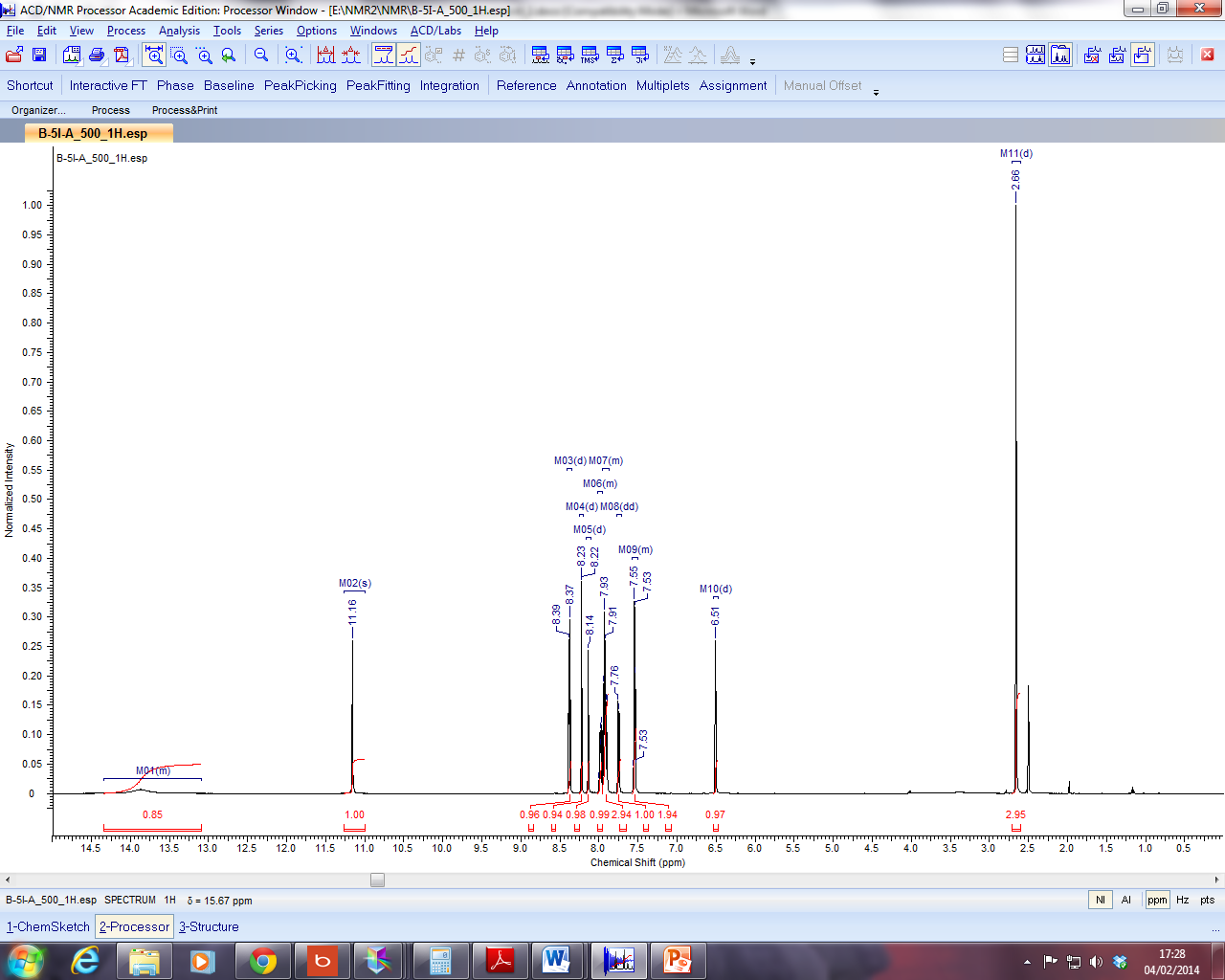

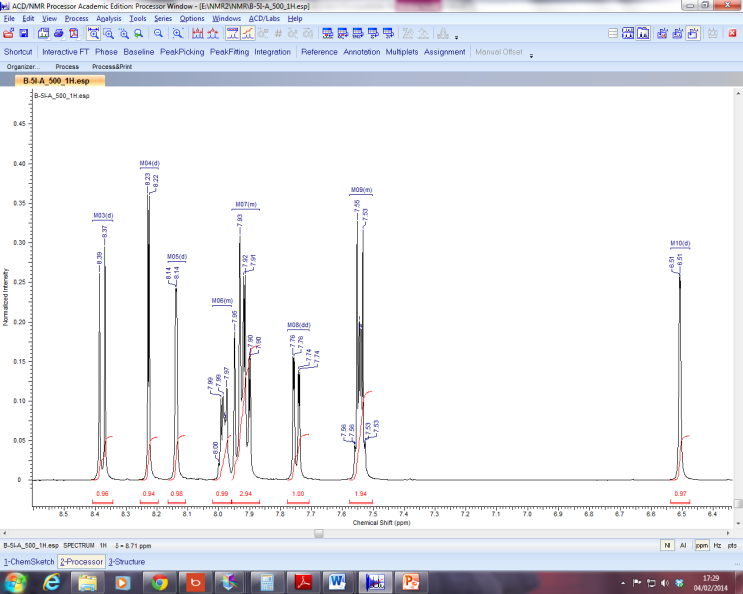


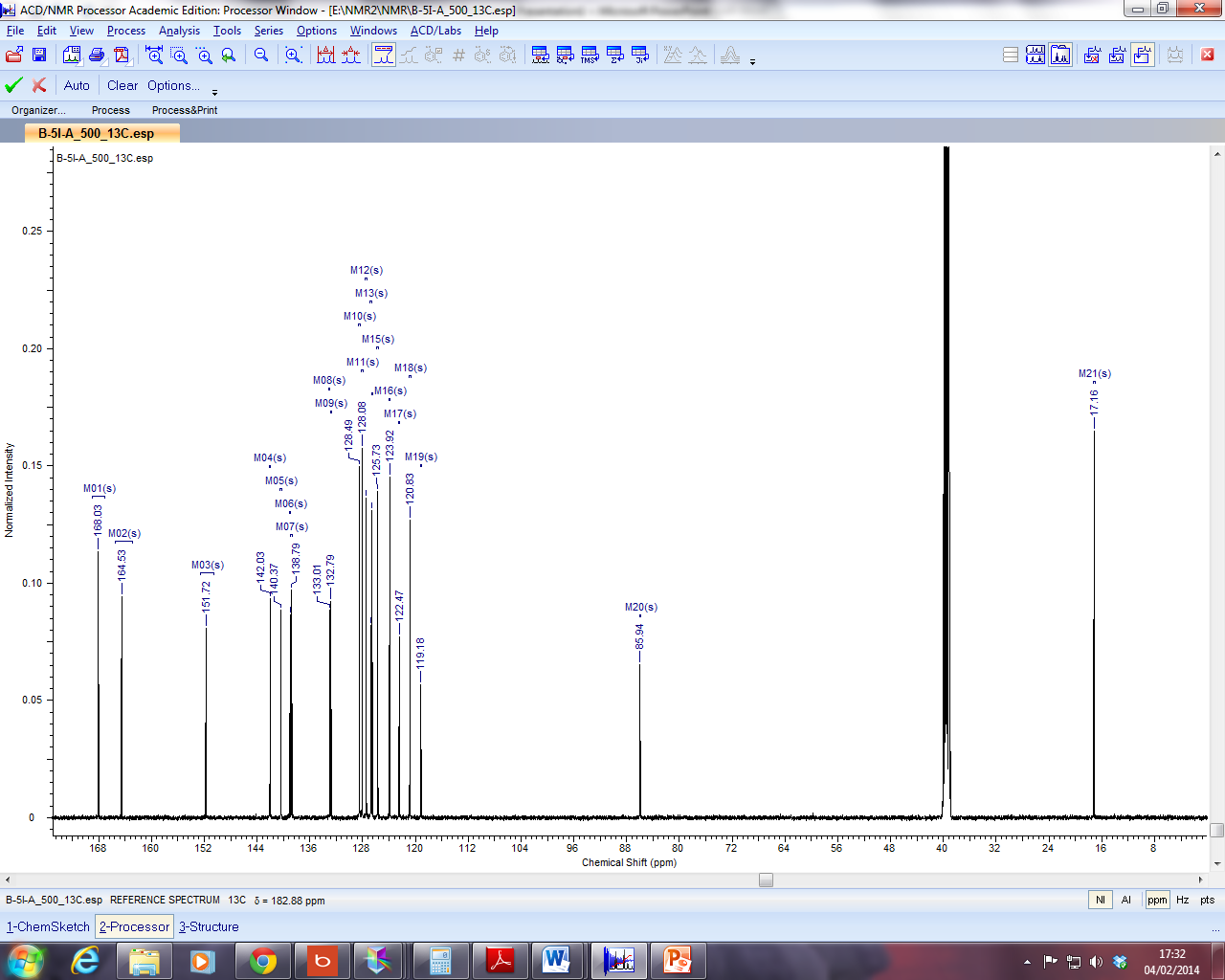

**2.2. (E)-2-(3-(naphthalen-2-yl)but-2-enamido)-5-(trimethylstannyl)benzoic acid (1d)**

**^1^H NMR** (*d_6_*-DMSO, 500 MHz, 20°C): δ = 13.52 (br. s, 1H, *H*1), 11.21 (s, 1H, *H*9), 8.51 (d, *J* = 8.2 Hz, 1H, *H*7), 8.17 (d, *J* = 1.4 Hz, 1H, *H*15), 8.08 (d, *J* = 1.2 Hz (84%), with Sn satellites (*J*^2^_Sn-H_ = 46 and 43 Hz (16%)), 1H, *H*4), 7.92 – 8.02 (m, 3H, *H*17+20+22), 7.79 (dd, *J*=8.7, 1.6 Hz, 1H, *H*23), 7.72 (dd, *J* = 8.2, 1.3 Hz (84%), with Sn satellites (*J*^2^_Sn-H_ = 47 and 44 Hz (16%)), 1H, *H*6), 7.56 (m, 2H, *H*18+19), 6.53 (d, *J* = 1.2 Hz, 1H, *H*11), 2.67 (d, *J* = 0.7 Hz, 3H, *H*13), 0.31 (s (84%), with Sn satellites (*J*^2^_Sn-H_ = 53 and 56 Hz (16%)), 9H, Sn(C*H*_3_)_3_). **^13^C NMR** (*d_6_*-DMSO, 125 MHz, 20°C): δ = 170.2 (*C*2), 164.4 (*C*10), 150.7 (*C*12), 140.8 (*C*6), 140.7 (*C*8), 138.9 (*C*14+*C*4), 133.0 (*C*21), 132.8 (*C*16), 131.4 (*C*5), 128.5 (*C*17), 128.1 (*C*20), 127.5 (*C*22), 126.7 (*C*18/19), 126.5 (*C*18/19), 125.6 (*C*15), 124.0 (*C*23), 122.4 (*C*7), 121.4 (*C*11), 119.5 (*C*3), 17.1 (*C*13), -9.2 (with Sn satellites; -7.6, -7.7, -10.4, -10.5), Sn(*C*H_3_)_3._ **ESMS** calcd for C_24_H_25_NNaO_3_Sn [M+Na]^+^ : 518.0753, found 518.0744. **HPLC** (method B): R_t_ = 11.97 min. **Elemental analysis** for C_24_H_25_NO_3_Sn, calc: C 58.3 %, H 5.1 %, N 2.8 %, found: C 57.9 %, H 5.2 %, N 2.9 %.


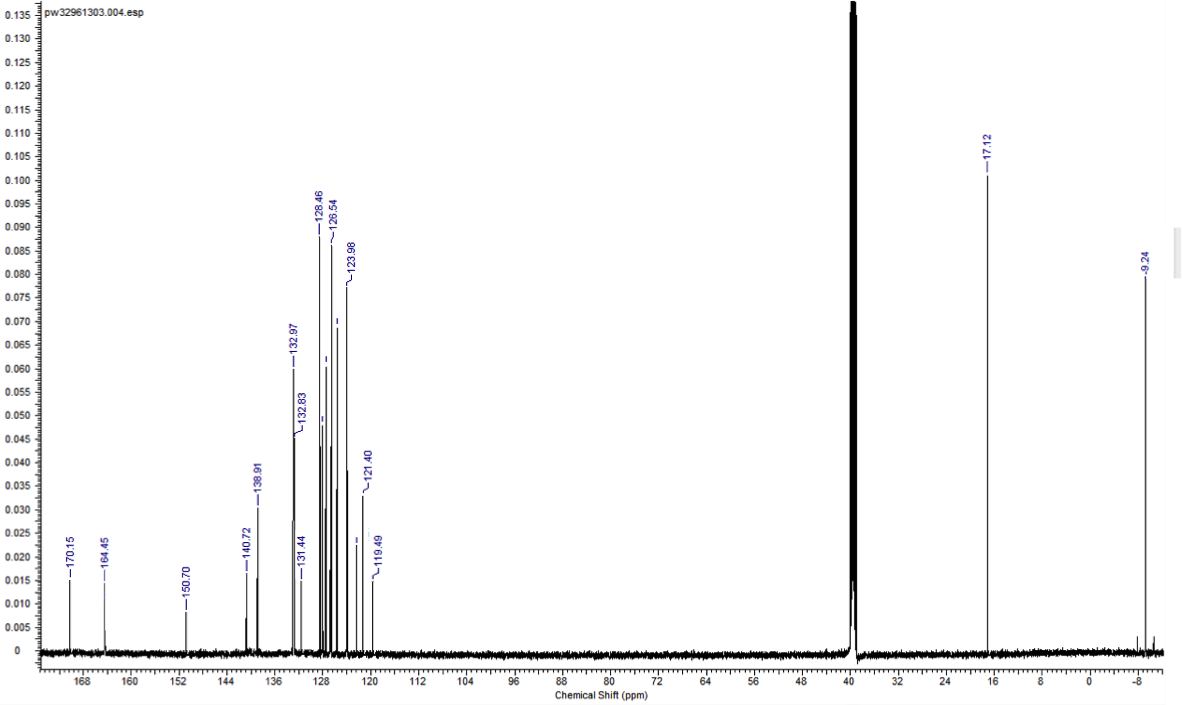


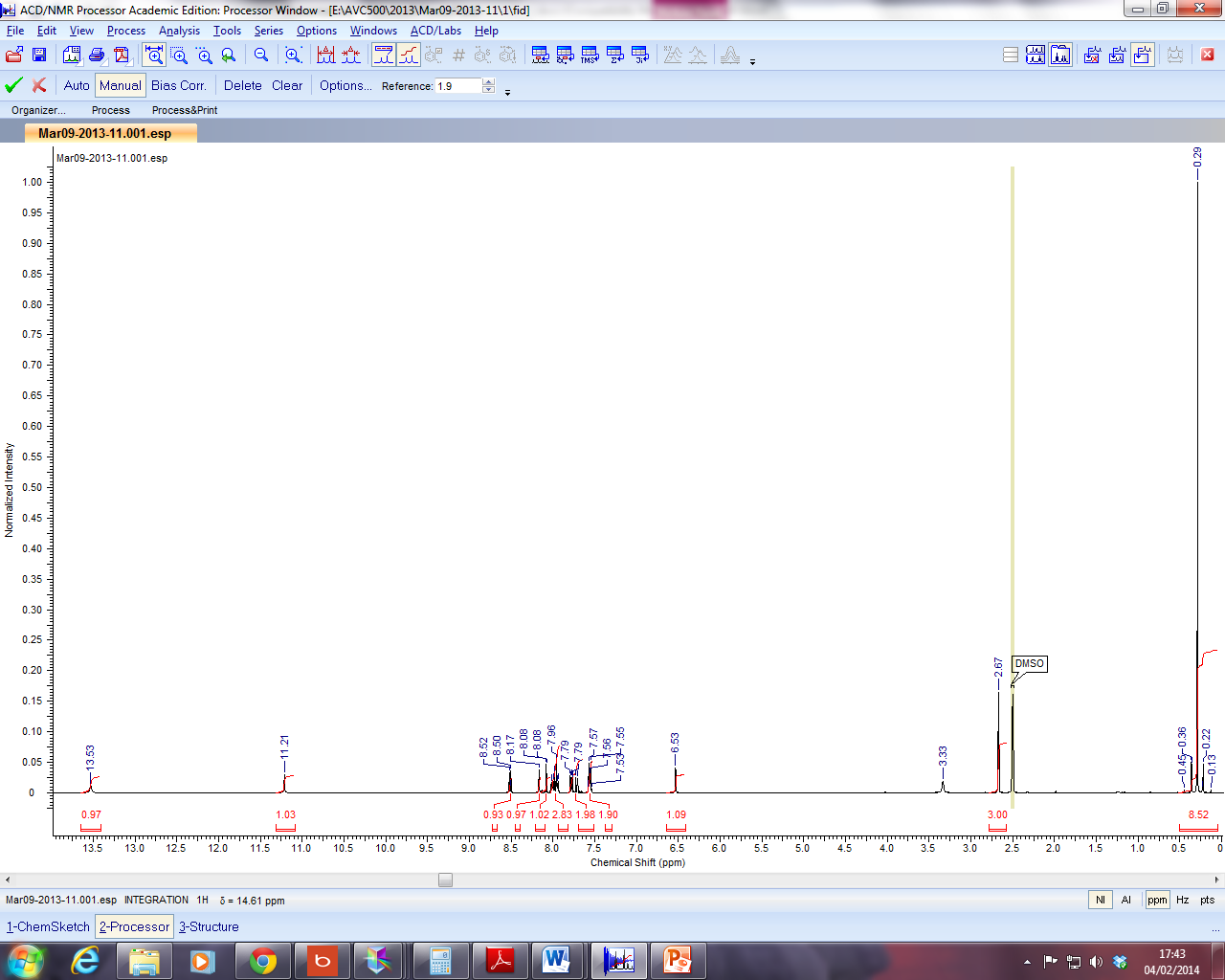

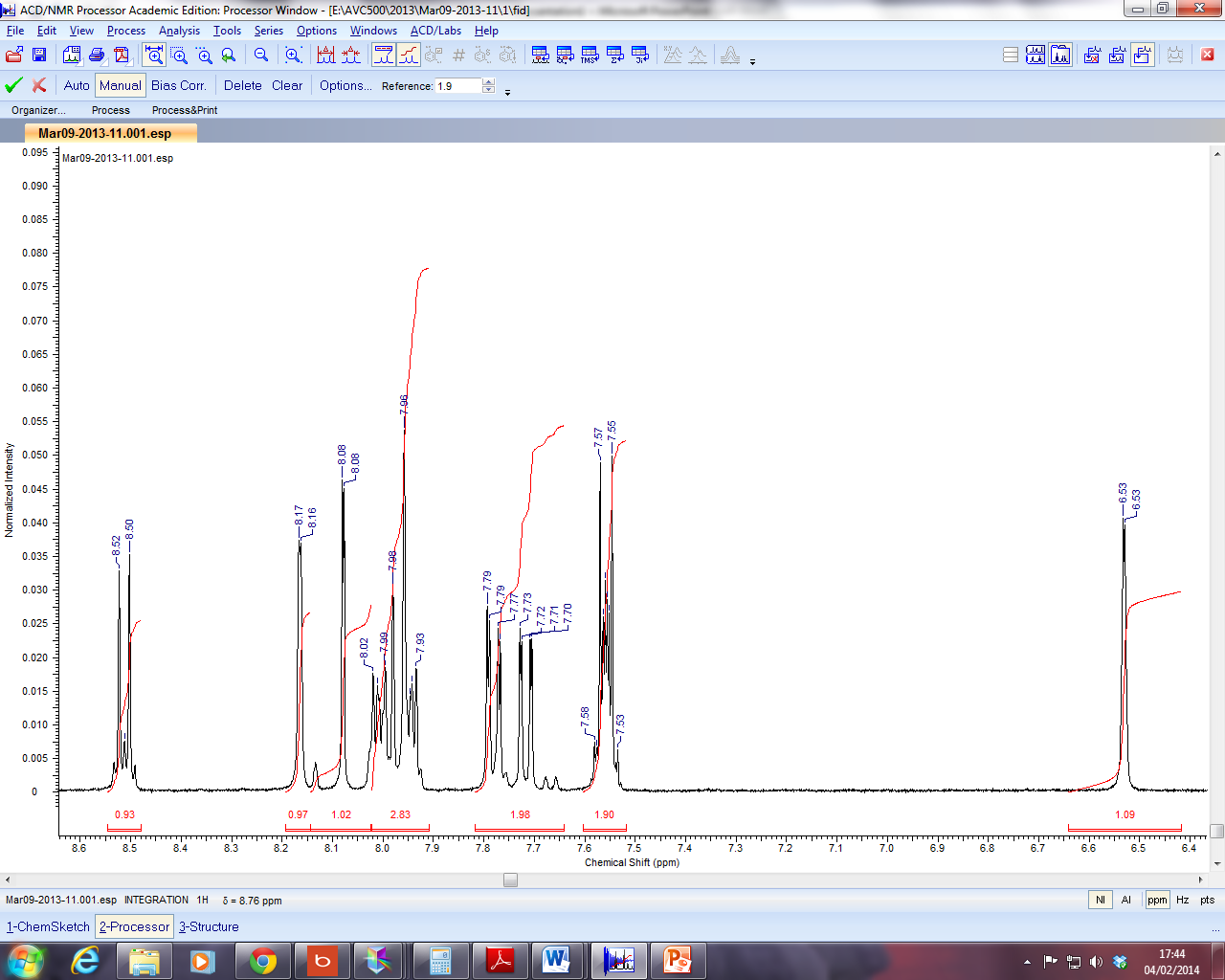

**2.3. (E)-4-iodo-2-(3-(naphthalen-2-yl)but-2-enamido)benzoic acid (2)**

**^1^H NMR** (*d_6_*-DMSO, 500 MHz, 20°C): δ = 11.97 (br. s, 2H, *H*1+9), 9.05 (d, *J* = 2.1 Hz, 1H, *H*7), 8.16 (d, *J* = 1.5 Hz, 1H, *H*15), 8.00 (m, 1H, *H*17), 7.96 (m, 2H, *H*20+22), 7.76 (dd, *J* = 8.3, 1.9 Hz, 1H, *H*23), 7.74 (d, *J* = 8.4 Hz, 1H, *H*4), 7.56 (m, 2H, *H*18+19), 7.51 (dd, *J* = 8.5, 1.9 Hz, 1H, *H*5), 6.50 (q, *J* = 1.1 Hz, 1H, *H*11), 2.68 (d, *J* = 0.9 Hz, 3H, *H*13). **^13^C NMR** (*d_6_*-DMSO, 125 MHz, 20°C): δ = 169.5 (*C*2), 165.1 (*C*10), 152.0 (*C*12), 142.1 (*C*6), 139.3 (*C*8), 133.5 (*C*4), 133.3 (*C*14), 133.1 (*C*21), 131.0 (*C*16), 129.0 (*C*17), 128.6 (*C*20/22), 128.0 (*C*20/22), 127.6 (*C*19), 126.7 (*C*18), 126.5 (*C*15), 126.2 (*C*23), 124.4 (*C*7), 121.3 (*C*11), 116.8 (*C*3), 102.6 (*C*5), 17.6 (*C*13). **ESMS** calcd for C_21_H_15_INO_3_ [M-H]^-^ : 456.0102, found 456.0093. **HPLC** (method B): R_t_ = 9.97 min. **Elemental analysis** for C_21_H_16_INO_3_, calc: C 55.2 %, H 3.5 %, N 3.1 %, found: C 54.8 %, H 3.7 %, N 3.3 %.


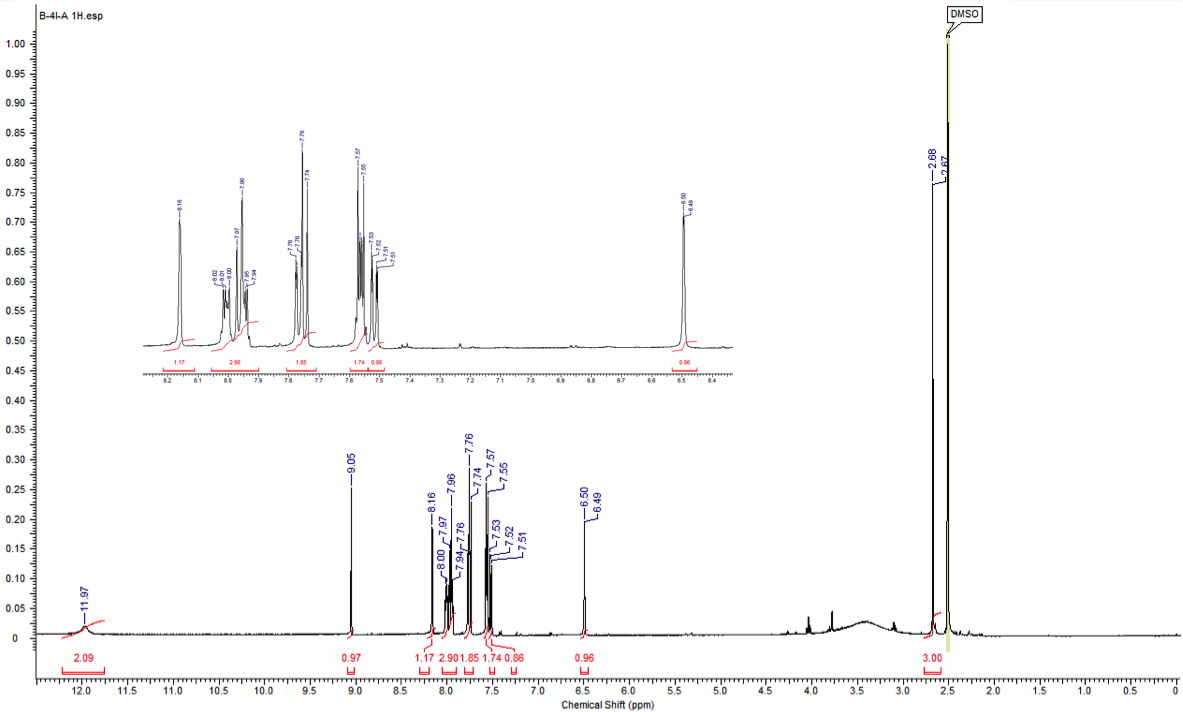


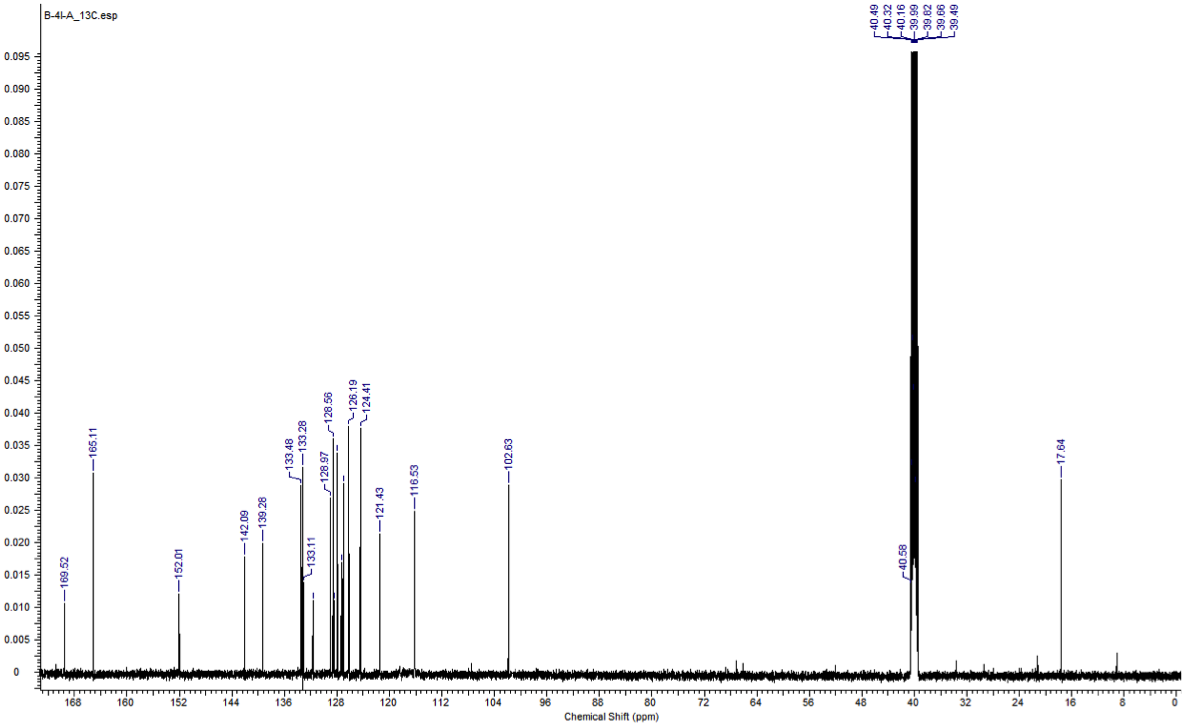

**2.4. 3-Iodo-2-(3,4-dihydroxyphenyl)-7,8-dihydroxy-4*H*-chromen-4-one (3)**

**^1^H NMR** (*d_6_*-DMSO, 500 MHz, 20°C): δ = 10.43 (br. s, 4H, CO*H*), 7.84 (s, 1H, *H*5), 7.55 (m, 2H, *H*2’+6’), 6.90 (d, 1H, *J* = 8.8 Hz, *H*3’), 6.61 (s, 1H, *H*3). **^13^C NMR** (*d_6_*-DMSO, 125 MHz, 20°C): δ = 175.4 (*C*4), 163.0 (*C*2), 150.4 (*C*7), 149.3 (*C*4’), 146.9 (*C*9), 145.6 (*C*5’), 132.6 (*C*8), 124.3 (*C*5), 122.1 (*C*1’), 119.2 (*C*10), 118.3 (*C*2’), 115.7 (*C*3’), 113.9 (*C*6’), 104.1 (*C*3), 83.1 (*C*6). **ESMS** calcd for C_15_H_16_IO_6_ [M-H]^-^ : 410.9371, found 410.9373. **IR:** ν = 1615, 1593, 1534, 1463, 1403, 1286, 1196, 1171, 1126, 1032 cm^-1^. **HPLC** (method A): R_t_ = 8.01 min. **Elemental analysis** for C_15_H_9_IO_6_, calc: C 43.7 %, H 2.2 %, found: C 43.8 %, H 2.4 %.


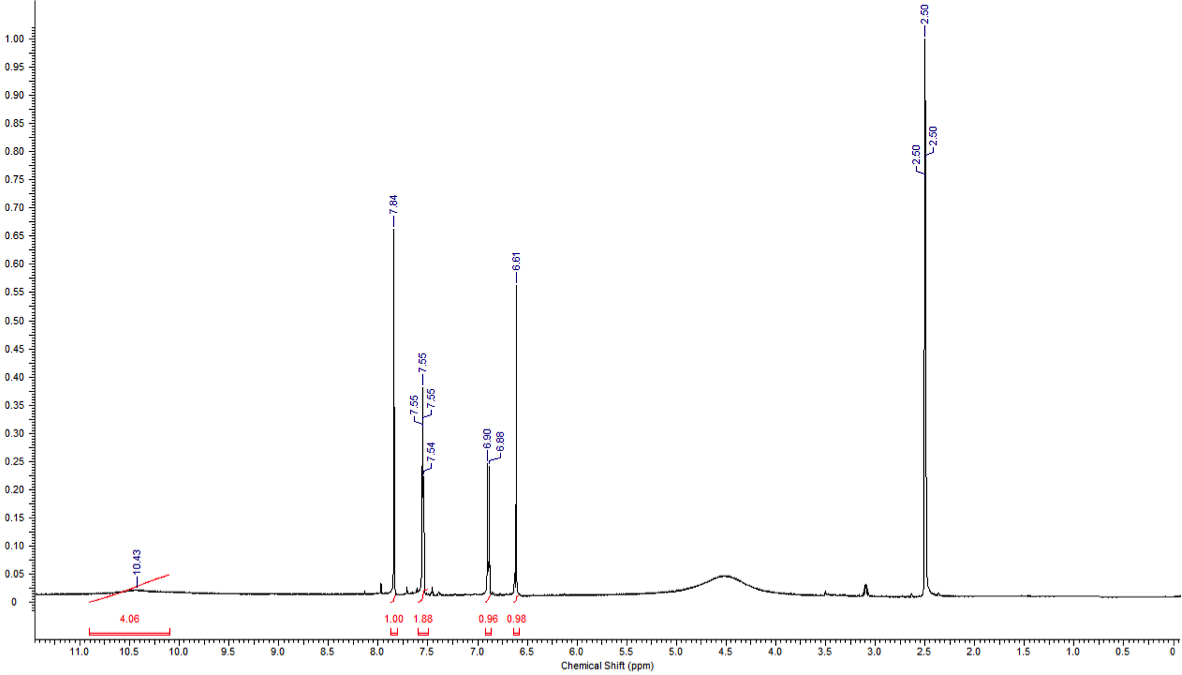


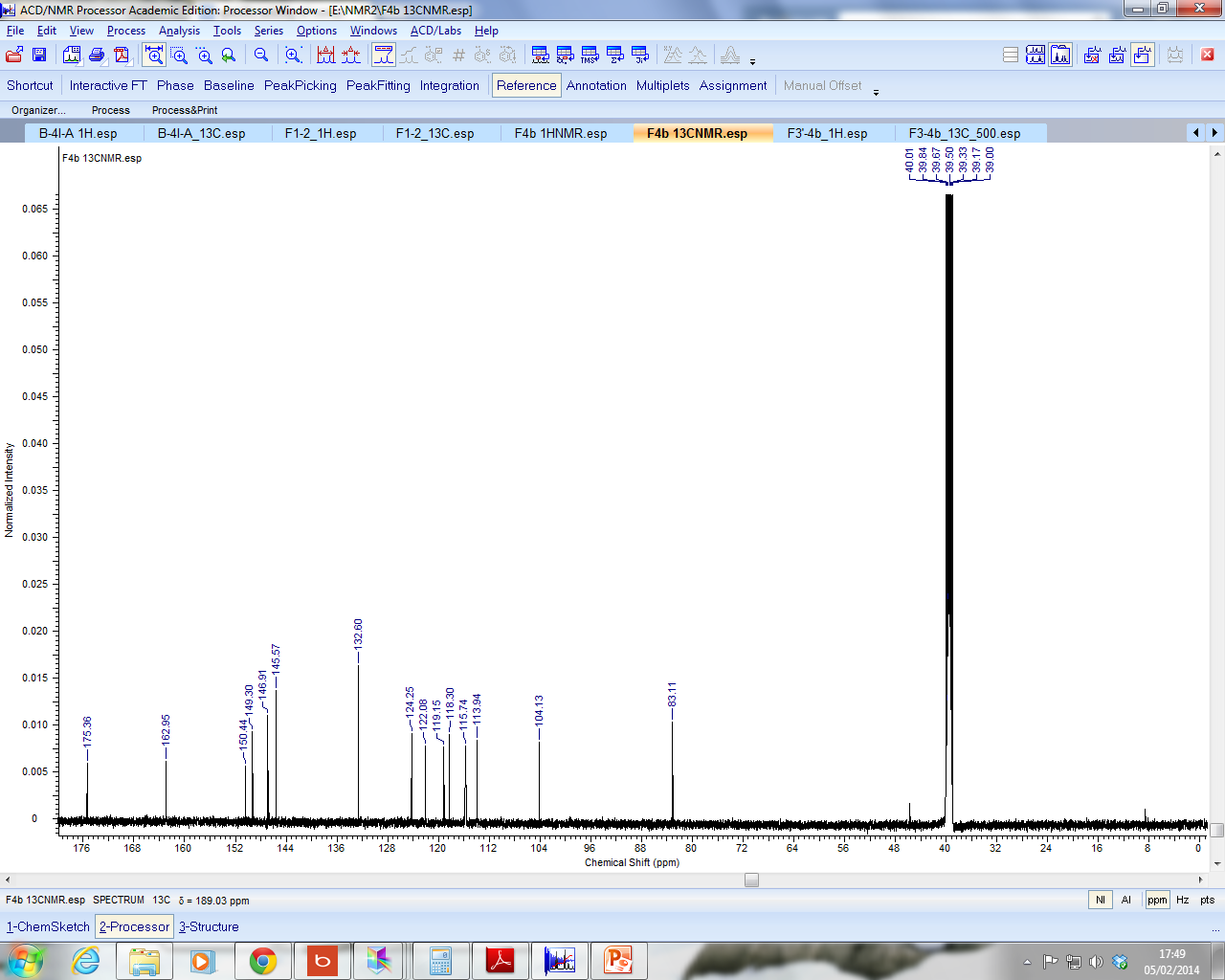

**2-(3,4- Diacetoxyphenyl)-7,8-diacetoxy-6-(trimethylstannyl)-4H-chromen-4-one (3g)**

**^1^H NMR** (CD_2_Cl_2_, 500 MHz, 20°C): δ = 8.14 (s (84%), with Sn satellites (*J*^2^_Sn-H_ = 42 and 44 Hz (16%)), 1H, *H*5), 7.70 (dd, *J* = 8.5, 2.2 Hz, 1H, *H*2’), 7.67 (d, *J* = 2.2 Hz, 1H, *H*6’), 7.36 (d, *J* = 8.5 Hz, 1H, *H*3’), 6.72 (s, 1H, *H*3), 2.41 (s, 3H, C(8)OC*H*_3_), 2.35 (s, 3H, C(7)OC*H*_3_), 2.32 (s, 3H, C(4’)OC*H*_3_), 2.32 (s, 3H, C(5’)OC*H*_3_), 0.38 (s (84%), with Sn satellites (*J*^2^_Sn-H_ = 55 and 57 Hz (16%)), 9H, Sn(C*H*_3_)_3_). **^13^C NMR** (CD_2_Cl_2_, 125 MHz, 20°C): δ = 177.3 (*C*4), 168.5 (*CO*4’/5’), 168.4 (*CO*4’/5’), 168.3 (*CO*7), 167.6 (*CO*8), 162.0 (*C*2), 151.6 (*C*7), 150.2 (*C*9), 145.3 (*C*5’), 143.3 (*C*4’), 134.3 (*C*6), 131.8 (*C*8), 130.8 (*C*1’), 130.7 (*C*5), 124.94 (*C*2’), 124.86 (*C*3’), 123.1 (*C*10), 122.2 (*C*6’), 109.1 (*C*3), 21.04 (*C*4’/5’), 21.02 (*C*4’/5’), 20.97 (*C*7), 20.7 (*C*8), -8.6 (with Sn satellites; -7.1, -7.2, -10.0, -10.1), Sn(*C*H_3_)_3_. **ESMS** calcd for C_26_H_26_NaO_10_Sn [M+Na]^+^ : 641.0445, found 641.0434. **IR:** ν = 1776, 1650, 1434, 1357, 1195, 1158, 1119, 1072, 1014 cm^-1^. **HPLC** (method A): R_t_ = 14.01 min


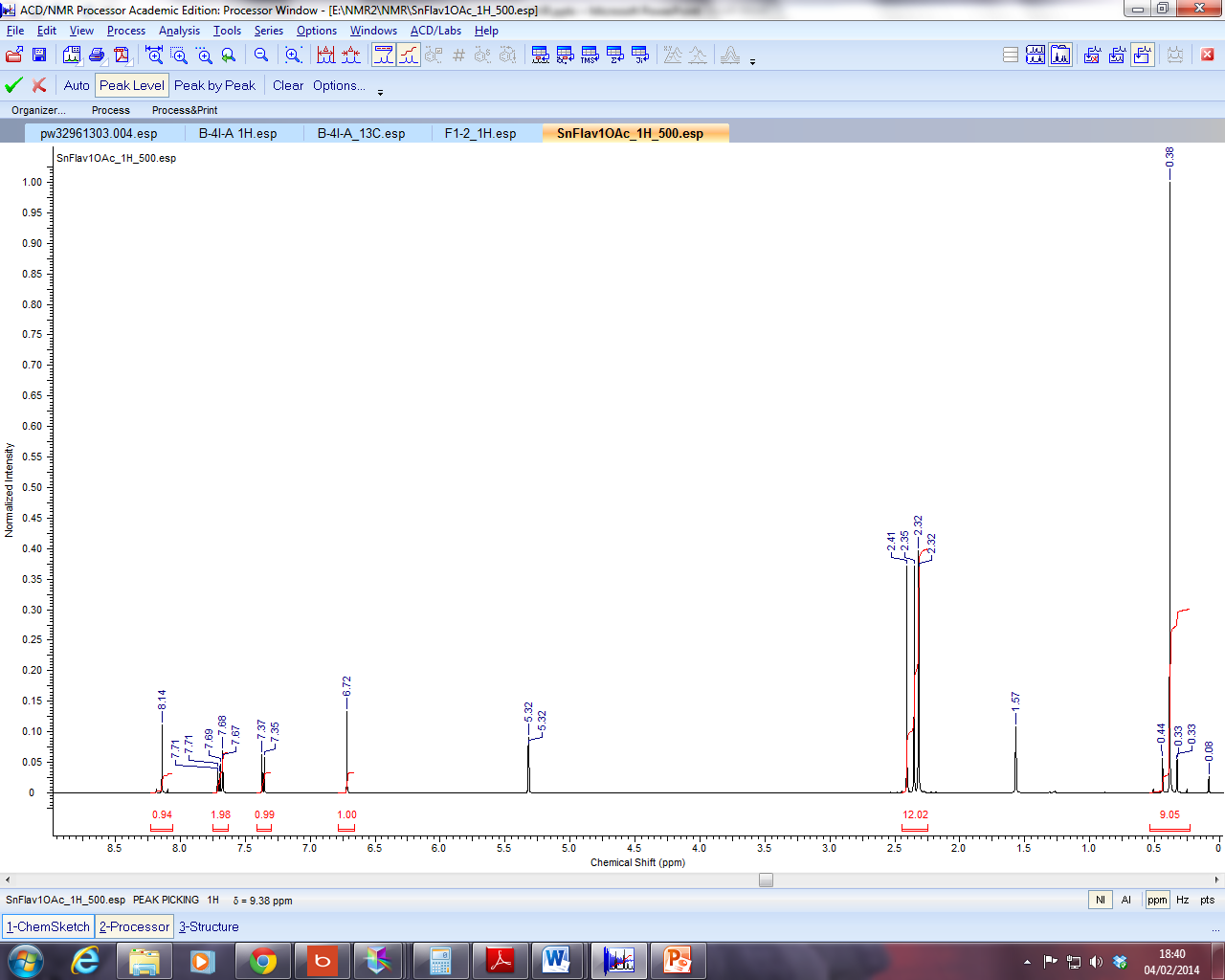

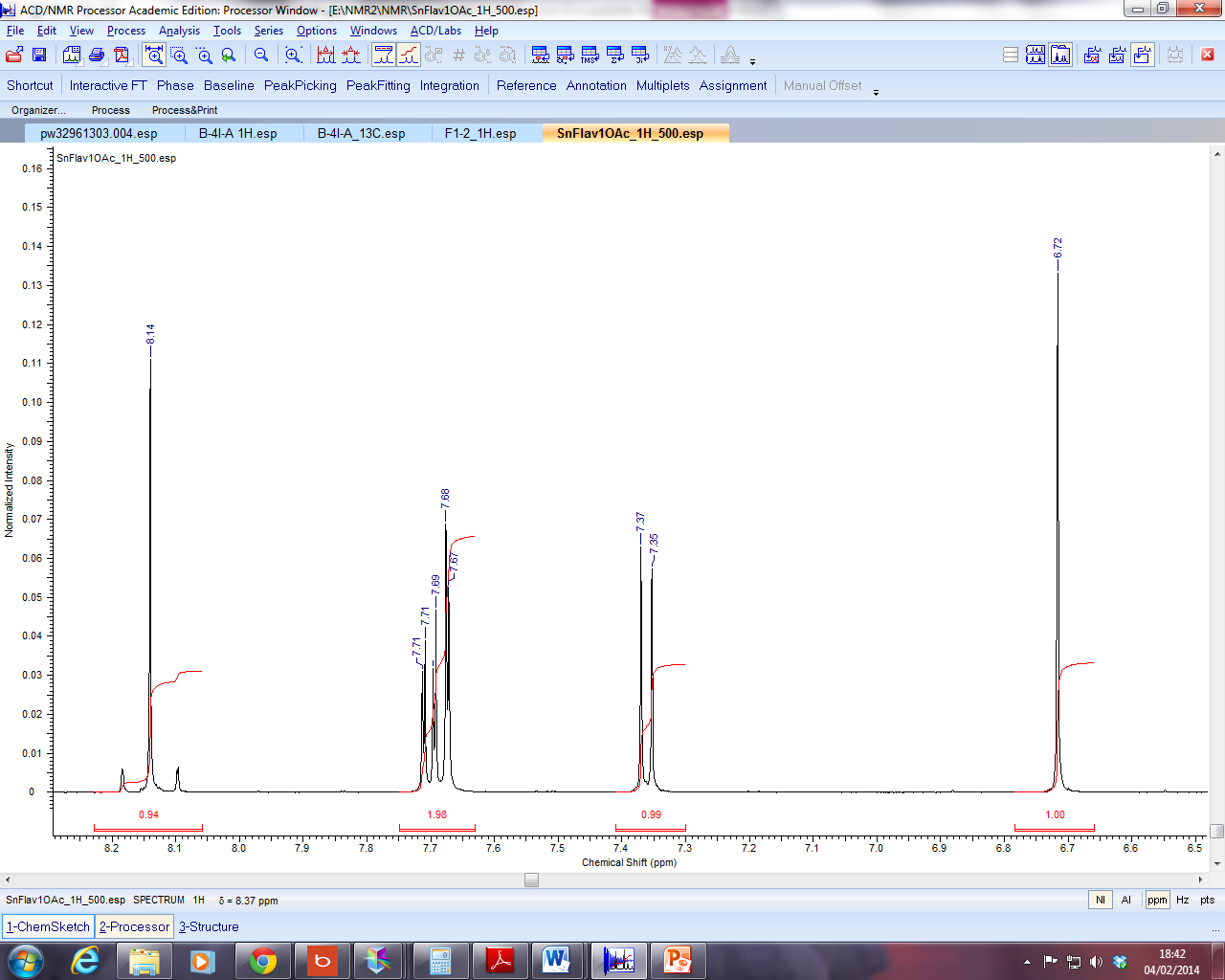


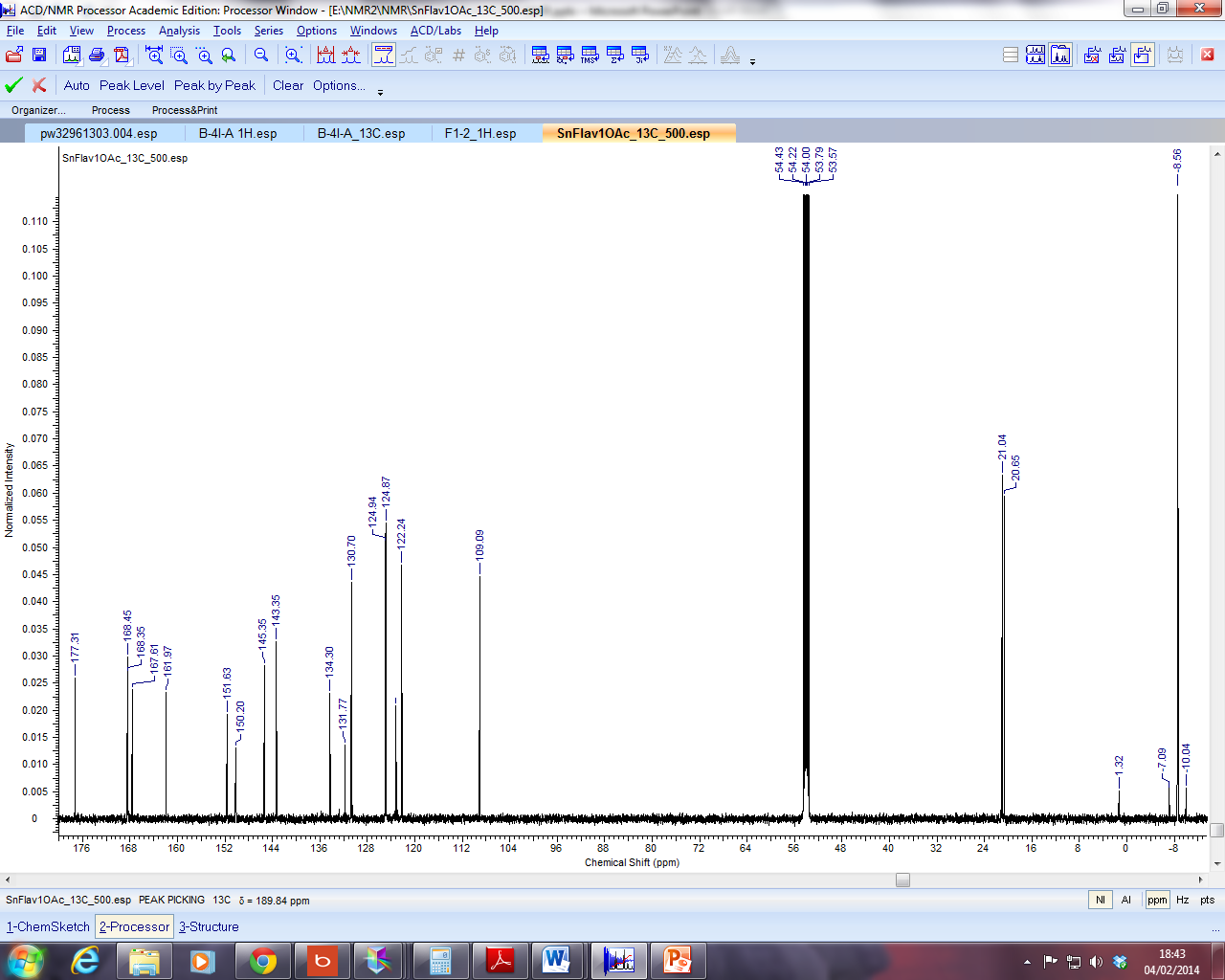

**2.5. 2-(3,4-Dihydroxy-5-iodophenyl)-7,8-dihydroxy-4H-chromen-4-one (5)**

**^1^H NMR** (*d_6_*-DMSO, 500 MHz, 20°C): δ = 10.35 (br. s., 2H, C(7+4’)O*H*), 10.04 (br. s., 1H, C(5’)O*H*), 9.45 (br. s., 1H, C(8)O*H*), 7.95 (d, *J* = 2.2 Hz, 1H, *H*6’), 7.46 (d, *J* = 2.2 Hz, 1H, *H*2’), 7.38 (d, *J* = 8.5 Hz, 1H, *H*5), 6.94 (d, *J* = 8.5 Hz, 1H, *H*6), 6.60 (s, 1H, *H*3). **^13^C NMR** (*d_6_*-DMSO, 125 MHz, 20°C): δ = 176.7 (*C*4), 160.9 (*C*2), 150.3 (*C*7), 149.0 (*C*4’), 146.5 (*C*9), 144.6 (*C*5’), 133.2 (*C*8), 127.3 (*C*6’), 124.2 (*C*1’), 117.0 (*C*10), 115.0 (*C*5), 113.8 (*C*6), 112.9 (*C*2’), 104.6 (*C*3), 85.3 (*C*3’). **ESMS** calcd for C_15_H_19_INaO_6_ [M+Na]^+^ : 434.9336, found 434.9331. **IR:** ν = 1623, 1572, 1547, 1429, 1405, 1308, 1213, 1190, 1137, 1032, 1011 cm^-1^**. HPLC** (method A): R_t_ = 7.75 min. **Elemental analysis** for C_15_H_9_IO_6_, calc: C 43.7 %, H 2.2 %, found: C 43.6 %, H 2.1 %.


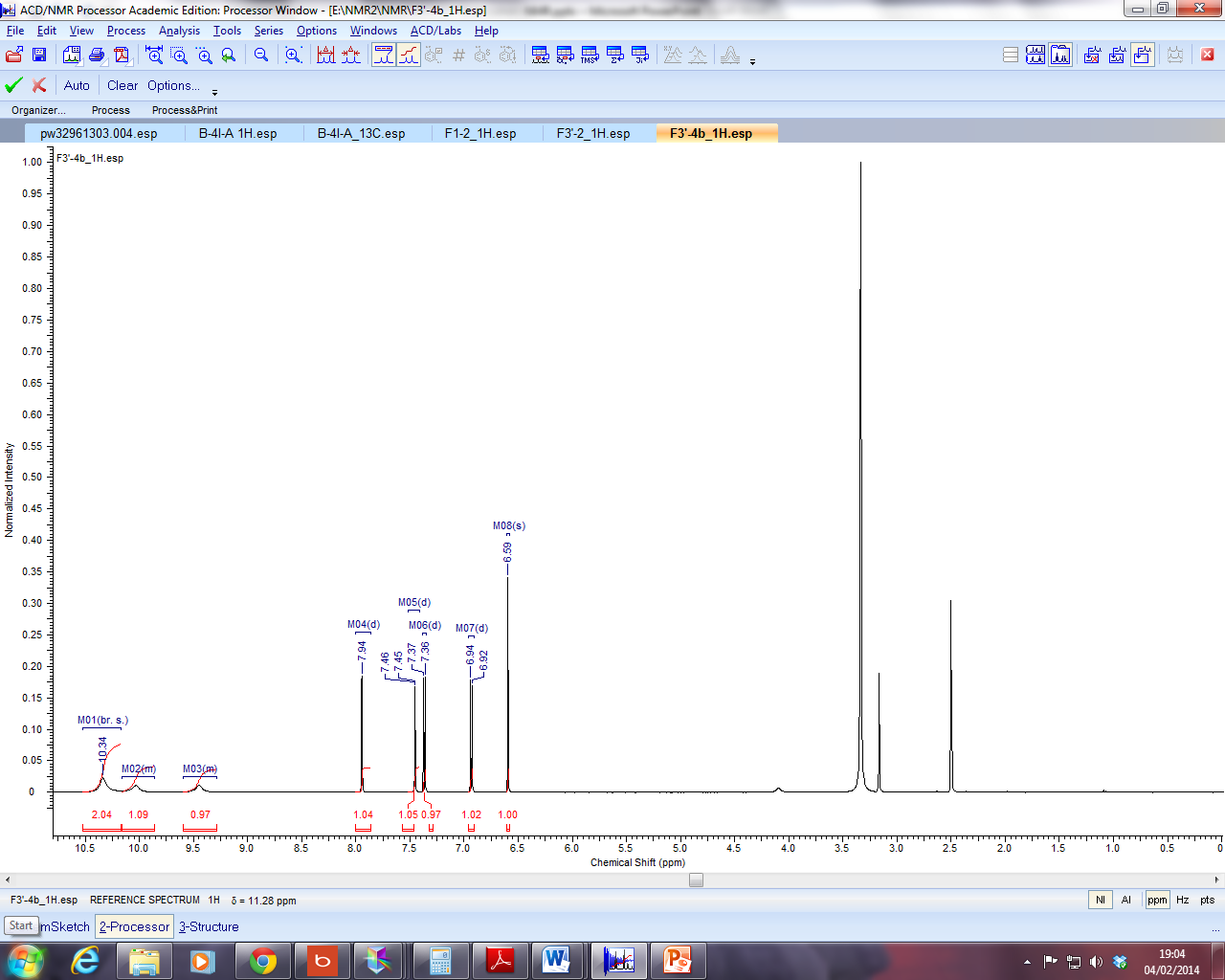

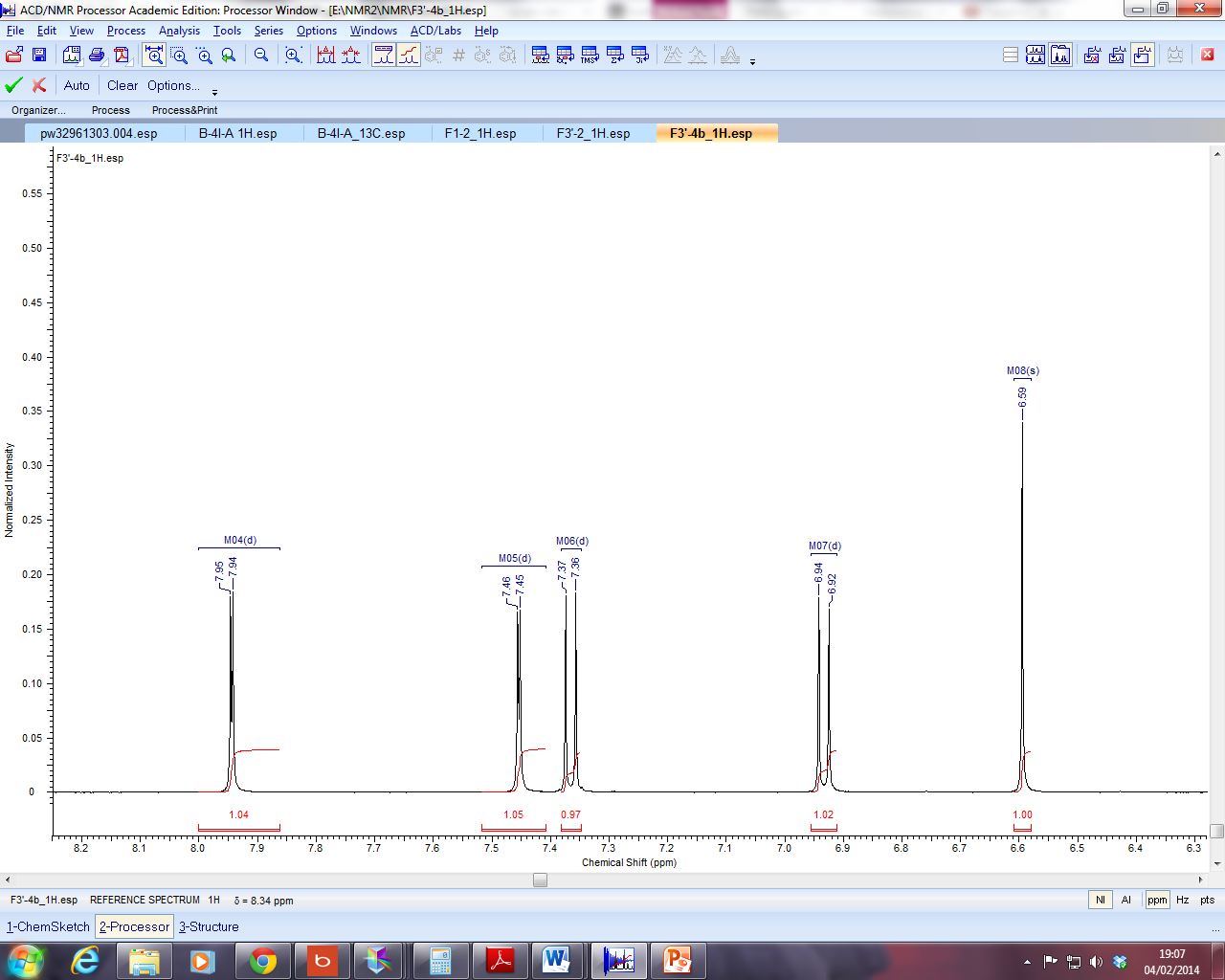


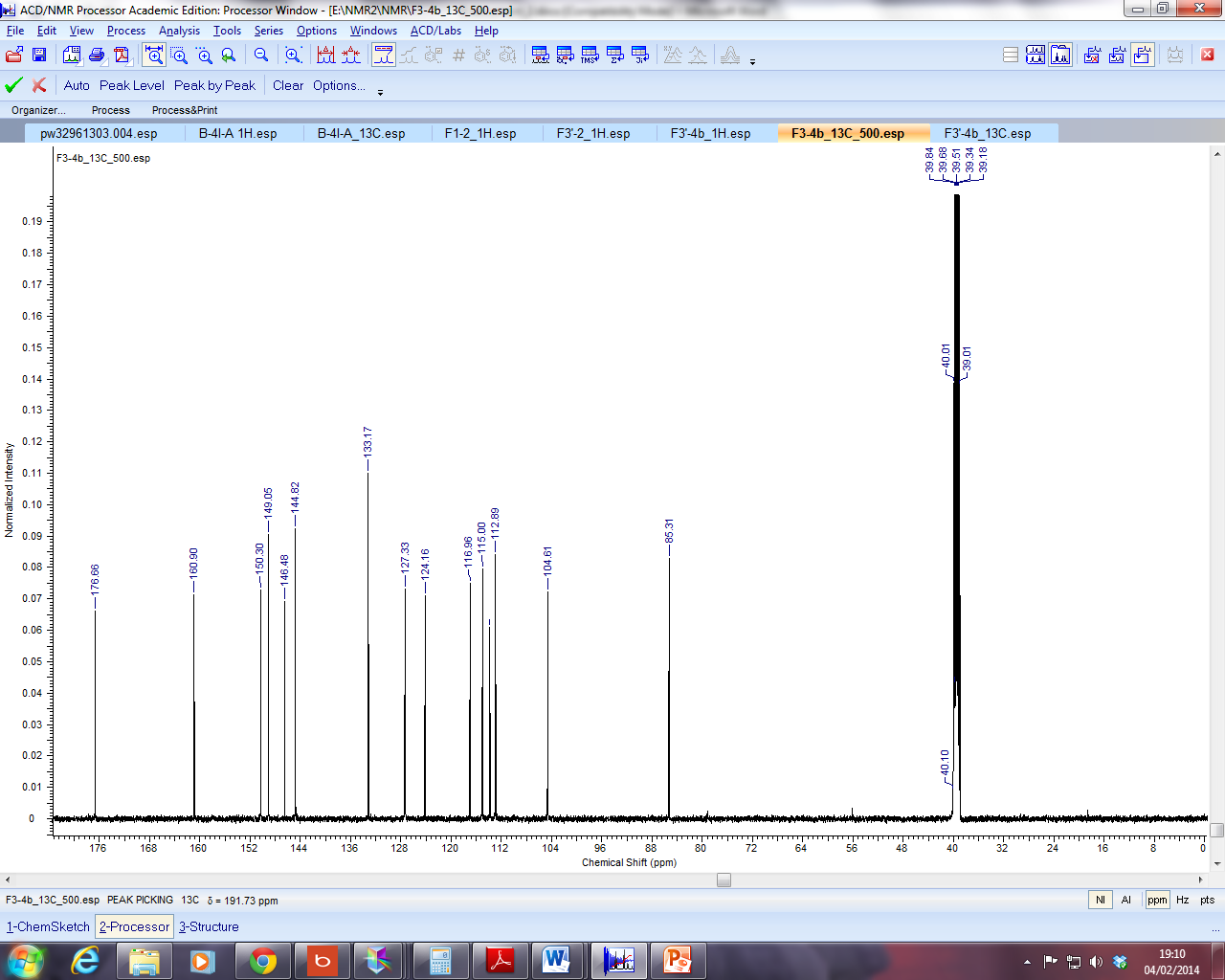

**2.6. 5-(7,8-Diacetoxy-4-oxo-4H-chromen-2-yl)-3-(trimethylstannyl)-1,2-phenylene diacetate (5g)**

**^1^H NMR** (CD_2_Cl_2_, 500 MHz, 20°C): δ = 8.07 (d, *J* = 8.8 Hz, 1H, *H*5), 7.77 (d, *J* = 2.2 Hz (84%), with Sn satellites (*J*^2^_Sn-H_ = 39 and 44 Hz (16%), 1H, *H*2’), 7.67 (d, *J* = 2.2 Hz, 1H, *H*6’), 7.27 (d, *J* = 8.6 Hz, 1H, *H*6), 6.74 (s, 1H, *H*3), 2.43 (s, 3H, C(8)OC*H*_3_), 2.35 (s, 3H, C(7)OC*H*_3_), 2.32 (s, 3H, C(4’)OC*H*_3_), 2.29 (s, 3H, C(5’)OC*H*_3_), 0.38 (s (84%), with Sn satellites (*J*^2^_Sn-H_ = 55 and 57 Hz (16%)), 9H, Sn(C*H*_3_)_3_). **^13^C NMR** (CD_2_Cl_2_, 125 MHz, 20°C): δ = 177.1 (*C*4), 168.7 (C(4’)O*C*OCH_3_), 168.5 (C(5’)O*C*OCH_3_), 168.3 (C(7)O*C*OCH_3_), 167.8 (C(8)O*C*OCH_3_), 162.8 (*C*2), 150.0 (*C*4’), 149.9 (*C*9), 147.3 (*C*7), 142.7 (*C*5’), 139.1 (*C*3’), 132.4 (*C*8), 131.8 (*C*2’), 130.7 (*C*1’), 123.4 (*C*5), 123.2 (*C*10), 122.8 (*C*6’), 120.6 (*C*6), 108.9 (*C*3), 21.2 (C(5’)OCO*C*H_3_), 21.0 (C(7)OCO*C*H_3_), 20.9 (C(4’)OCO*C*H_3_), 20.6 (C(8)OCO*C*H_3_), -8.6 (with Sn satellites; -7.1, -7.2, -10.0, -10.1) (Sn(*C*H_3_)_3_). **ESMS** calcd for C_26_H_26_NaO_10_Sn [M+Na]^+^ : 641.0440, found 641.0449. **IR:** ν =1774, 1652, 1367, 1260, 1202, 1136, 1074, 1034, 1015, 796 cm^-1^**. HPLC** (method A): R_t_ = 14.18 min.


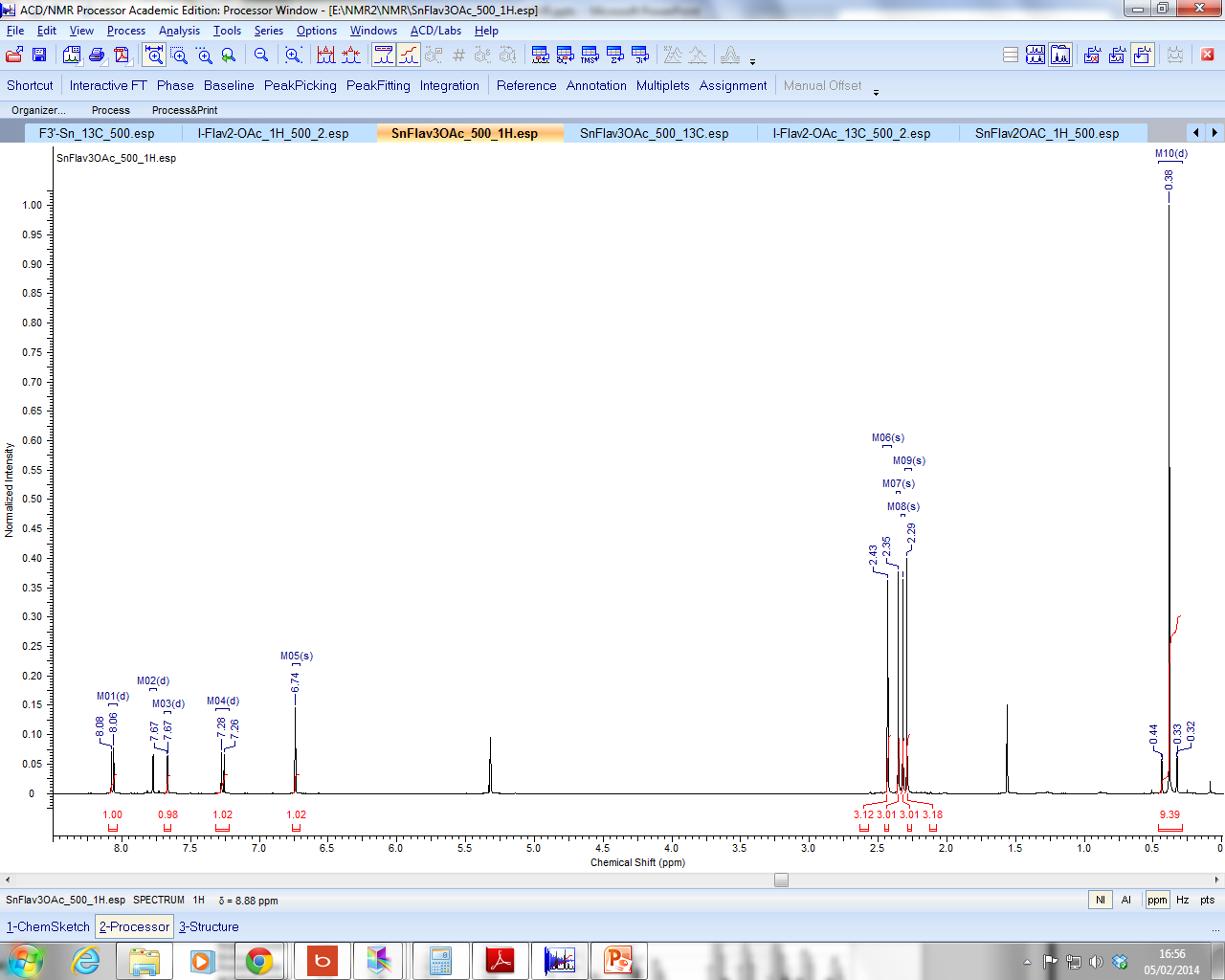

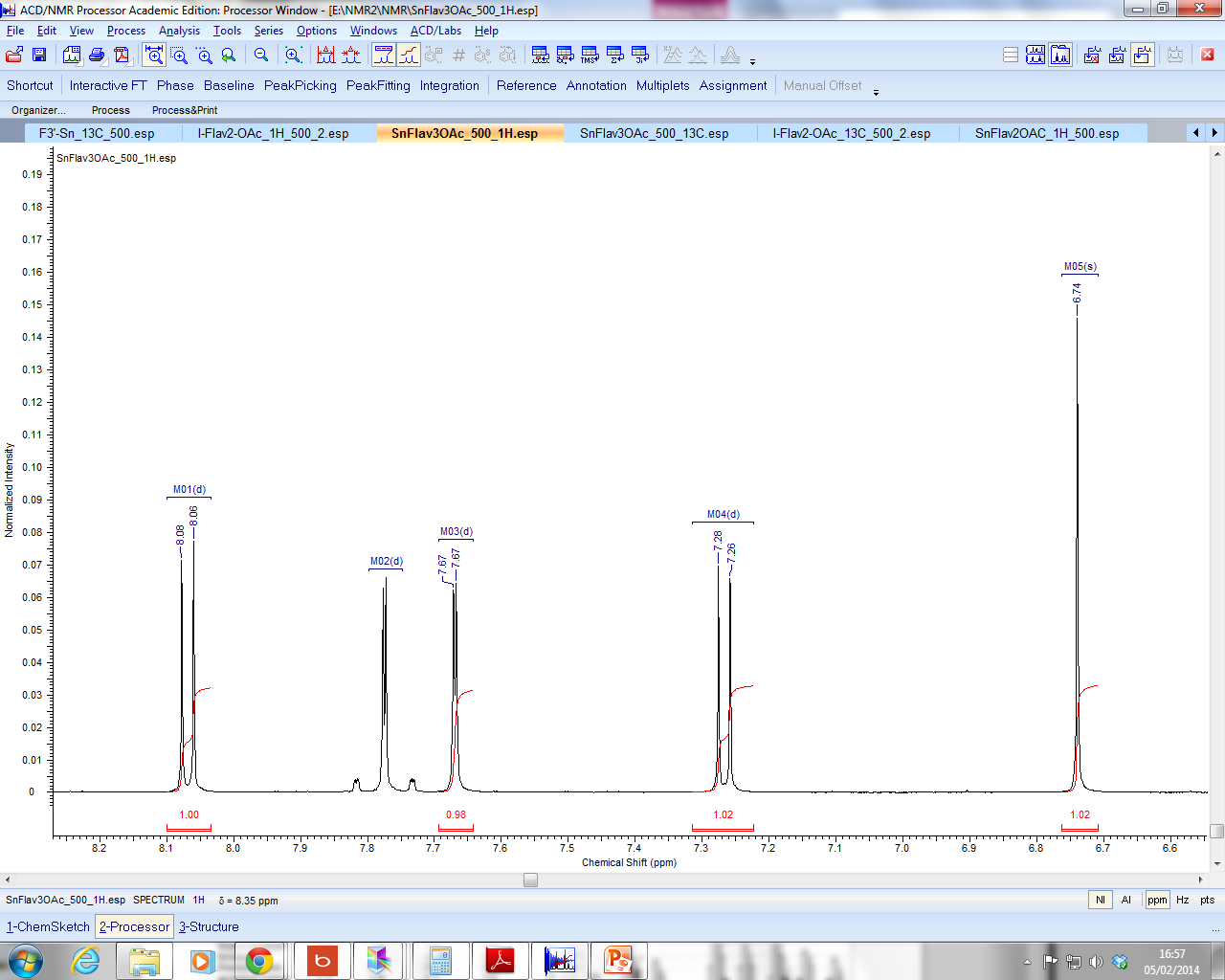


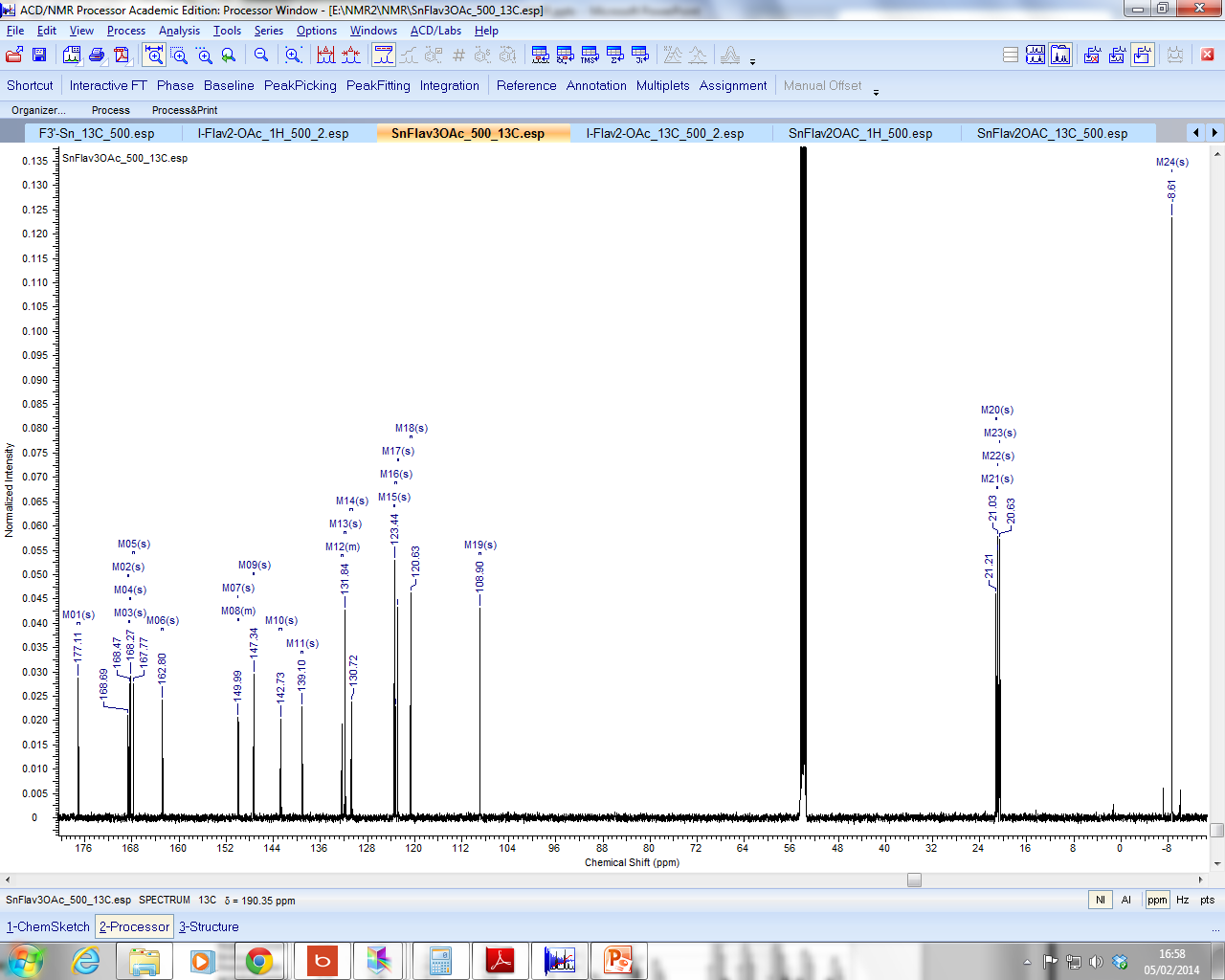


**2.7. 2-(3,4-dihydroxy-6-iodophenyl)-7,8-dihydroxy-4H-chromen-4-one (4)**

**^1^H NMR** (*d_6_*-DMSO, 500 MHz, 20°C): δ = 10.36 (s, 1H, C(7)O*H*), 9.92 (s, 1H, C(4’)O*H*), 9.64 (s, 1H, C(5’)O*H*), 9.32 (s, 1H, C(8)O*H*), 7.41 (d, *J* = 8.7 Hz, 1H, *H*5), 7.32 (s, 1H, *H*3’), 7.04 (s, 1H, *H*6’), 6.96 (d, *J* = 8.5 Hz, 1H, *H*6), 6.23 (s, 1H, *H*3). **^13^C NMR** (*d_6_*-DMSO, 125 MHz, 20°C): δ = 176.6 (*C*4), 165.1 (*C*2), 150.3 (*C*7), 148.6 (*C*4’), 146.9 (*C*9), 145.7 (*C*5’), 133.3 (*C*8), 128.5 (*C*1’), 126.0 (*C*3’), 117.6 (*C*6’), 116.9 (*C*10), 114.9 (*C*5), 114.0 (*C*6), 110.7 (*C*3), 83.1 (*C*2’). **ESMS** calcd for C_15_H_9_INaO_6_ [M+Na]^+^ : 434.9336, found 434.9329. **IR:** ν = 1630, 1581, 1551, 1429, 1403, 1367, 1269, 1222, 1185, 1026, 1007 cm^-1^**. HPLC** (method A): R_t_ = 7.25 min. **Elemental analysis** for C_15_H_9_IO_6_, calc: C 43.7 %, H 2.2 %, found: C 43.5 %, H 2.4 %.


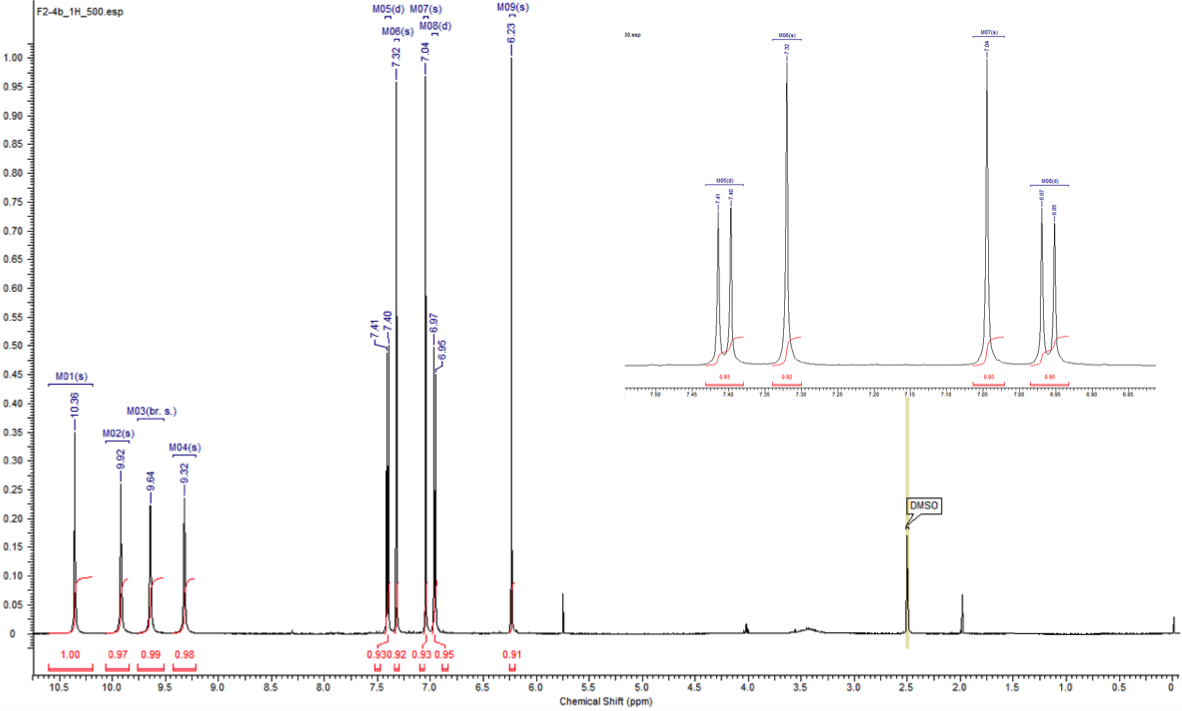


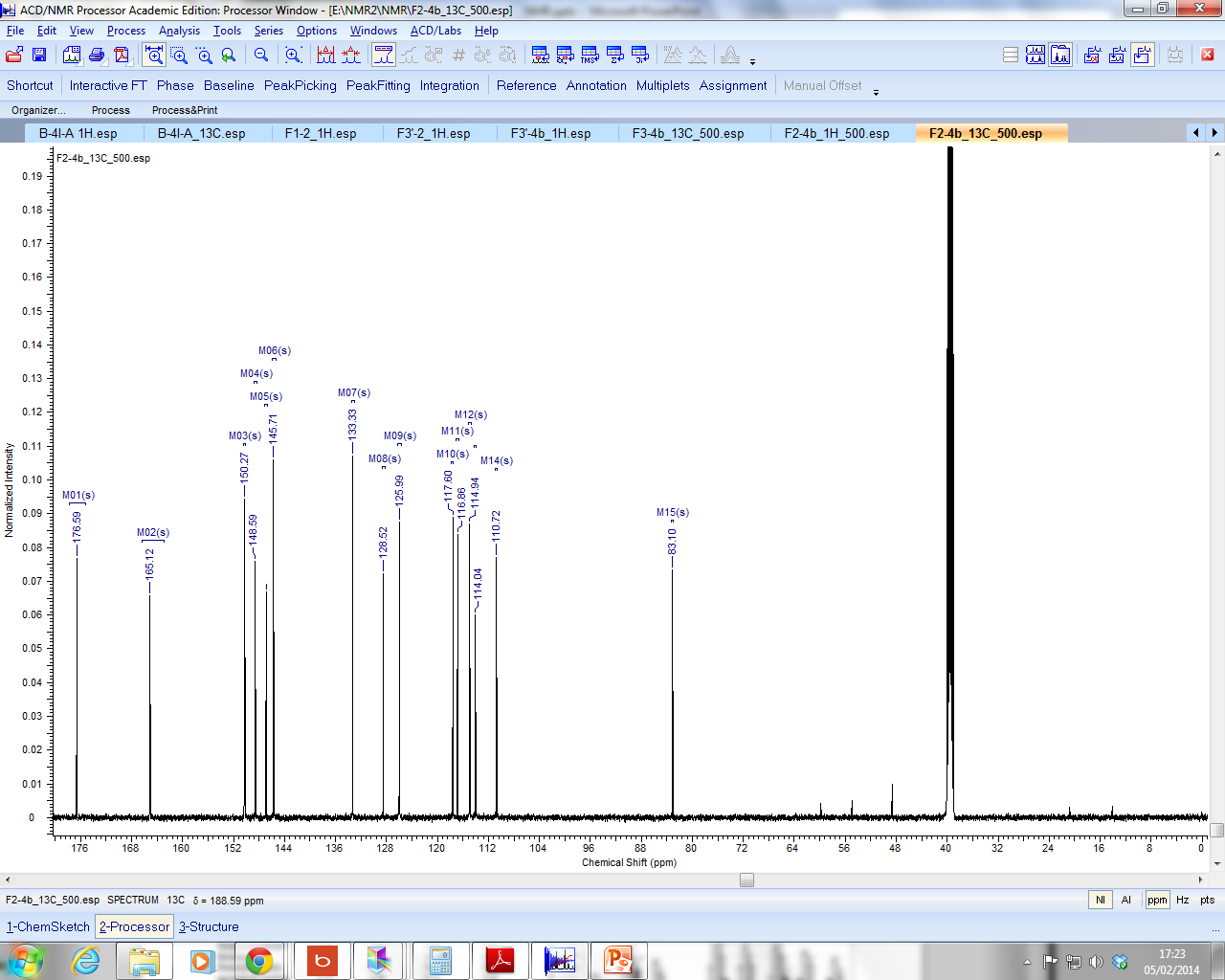

**2.8. 2-(4,5-diacetoxy-2-(trimethylstannyl)phenyl)-4-oxo-4H-chromene-7,8-diyl diacetate (4g)**

**^1^H NMR** (CD_2_Cl_2_, 500 MHz, 20°C): δ = 8.09 (d, *J* = 8.8 Hz, 1H, *H*5), 7.44 (s, (84%), with Sn satellites (*J*^2^_Sn-H_ = 39 and 44 Hz (16%)),1H, *H*6’), 7.42 (s, 1H, *H*3’), 7.30 (d, *J* = 8.8 Hz, 1H, *H*6), 6.39 (s, 1H, *H*3), 2.34 (s, 6H, C(7+8)OCOC*H*_3_), 2.32 (s, 3H, C(4’)OCOC*H*_3_), 2.30 (s, 3H, C(5’)OCOC*H*_3_), 0.33 ppm (s (84%), with Sn satellites (*J*^2^_Sn-H_ = 54 and 56 Hz (16%)), 9H, Sn(C*H*_3_)_3_). **^13^C NMR** (CD_2_Cl_2_, 125 MHz, 20°C): δ = 176.6 (*C*4), 168.7 (C(4’)O*C*OCH_3_), 168.6 (C(5’)O*C*OCH_3_), 168.2 (C(7/8)O*C*OCH_3_), 167.9 (C(7/8)O*C*OCH_3_), 166.4 (*C*2), 150.2 (*C*9), 147.4 (*C*7), 144.1 (*C*4’), 143.0 (*C*2’), 142.7 (*C*5’), 139.0 (*C*1’), 132.32 (*C*8), 132.29 (*C*3’), 124.5 (*C*6’), 123.5 (*C*5), 123.0 (*C*10), 120.8 (*C*6), 112.3 (*C*3), 21.05 (C(7+5’)OCO*C*H_3_), 21.01 (C(4’)OCO*C*H_3_), 20.7 (C(8)OCO*C*H_3_), -7.3 (with Sn satellites; -7.3, -7.4, -10.2, -10.3) (Sn(*C*H_3_)_3_). **ESMS** calcd for C_26_H_26_NaO_10_Sn [M+Na]^+^ : 641.0445, found 641.0436. **IR:** ν = 1775, 1655, 1368, 1260, 1192, 1162, 1137, 1074, 1036, 1013, 775 cm^-1^**. HPLC** (method A): R_t_ = 13.80 min.


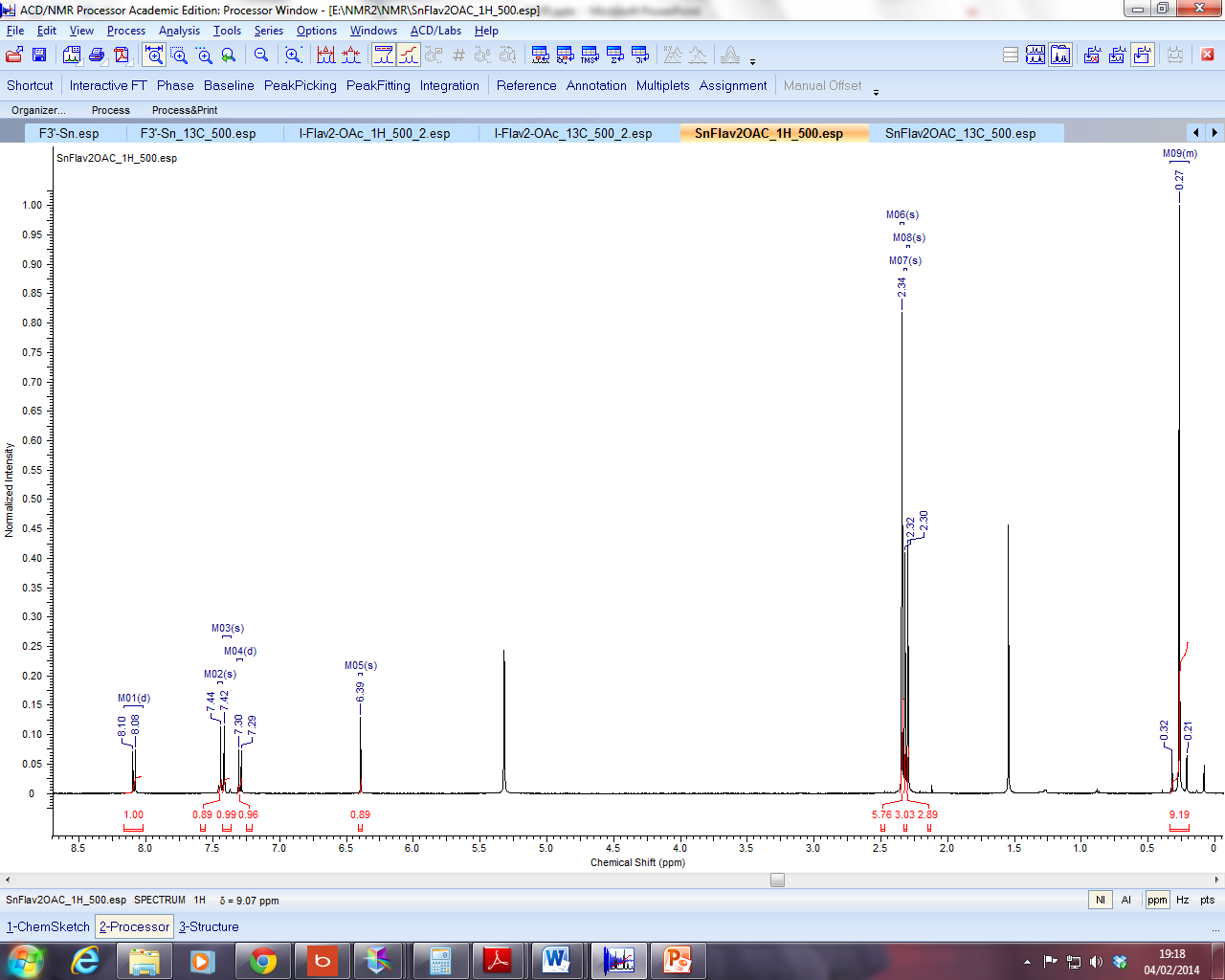

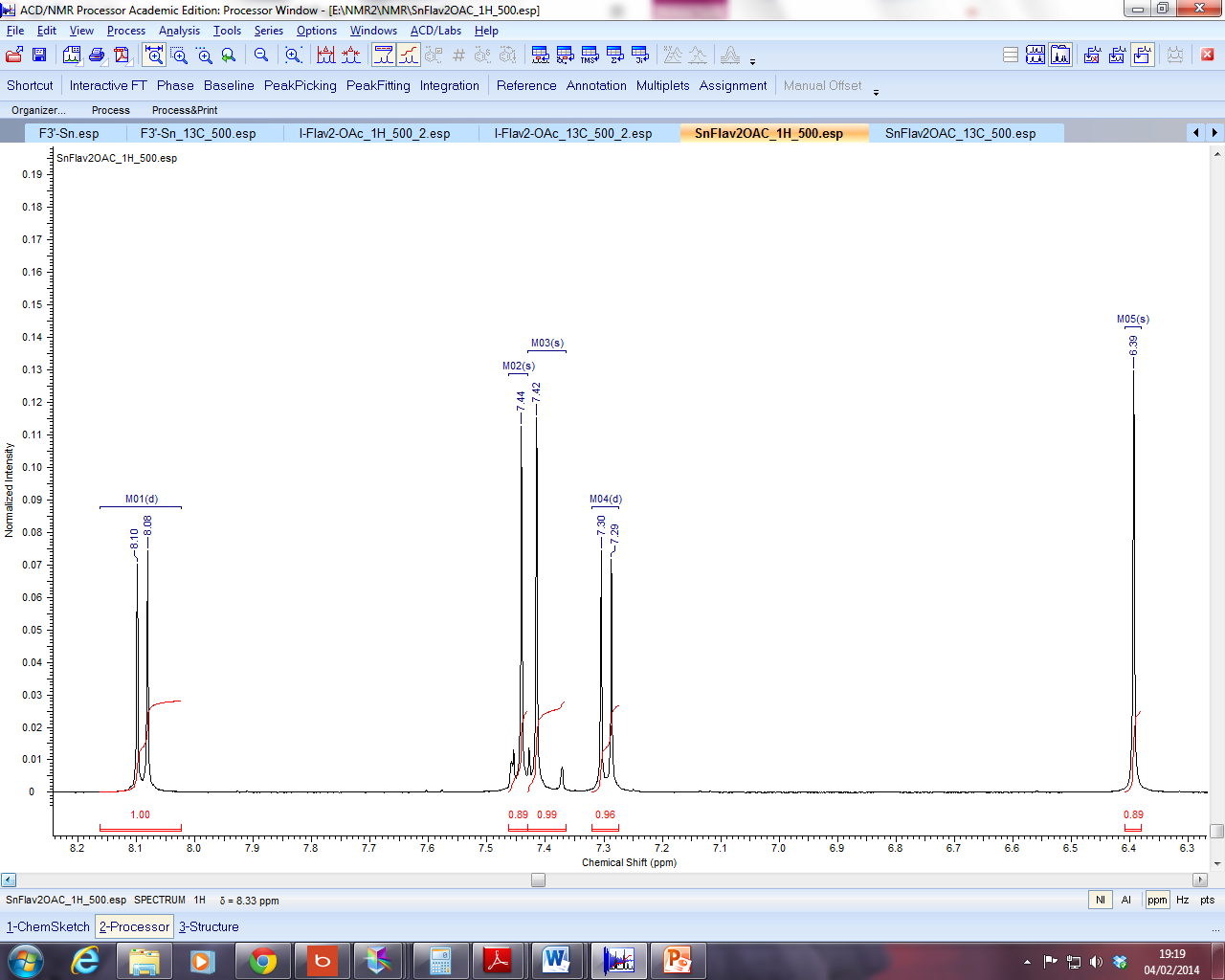


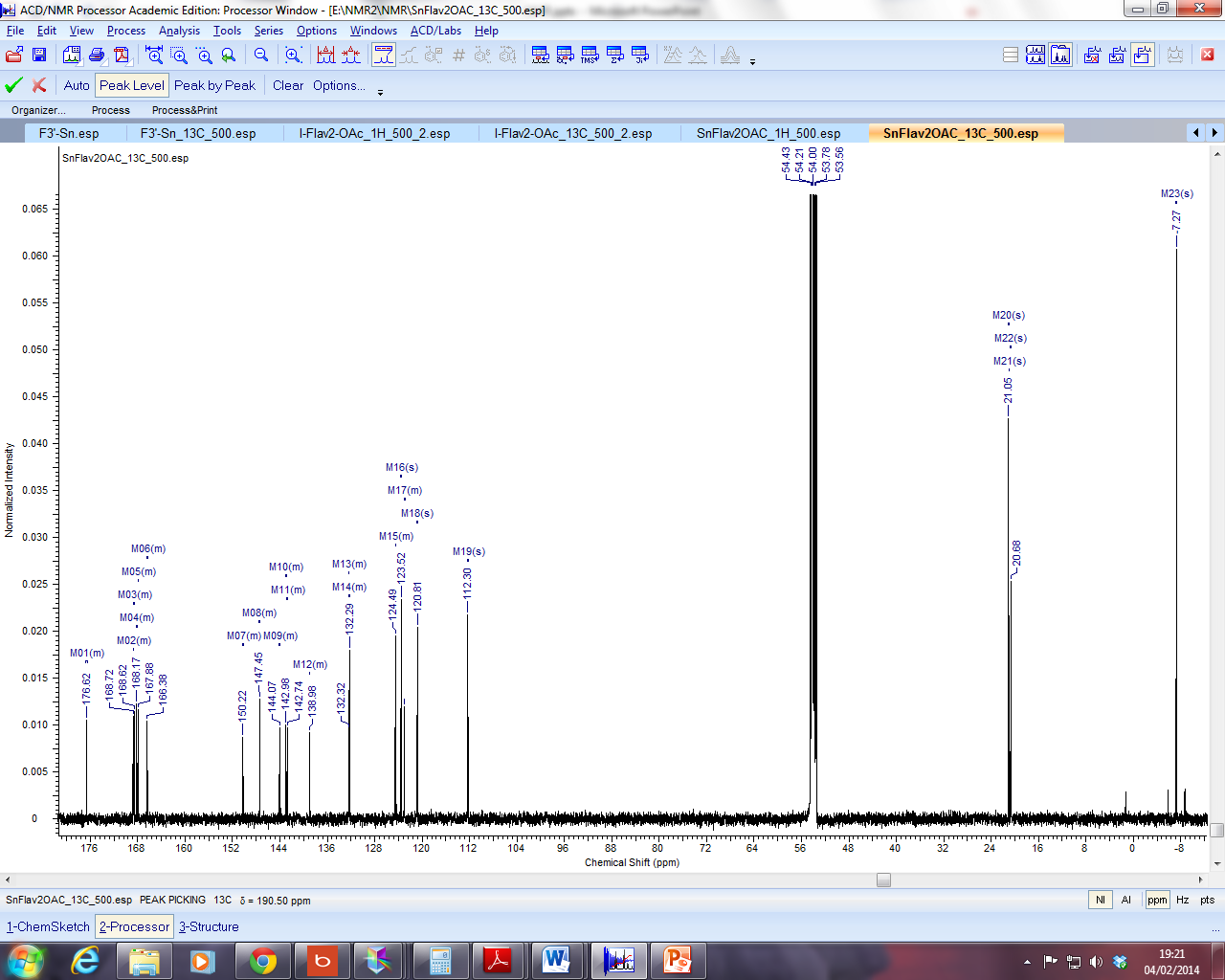

**2.9. *N*,*N*'-(5-Iodo-1,3-phenylene)bis(2,3-dihydroxybenzamide) (6)**

**^1^H NMR** (*d_6_*-DMSO, 400 MHz, 20°C): δ = 11.37 (s, 2H, *H*13), 10.43 (s, 2H, *H*14), 9.50 (br. s., 2H, *H*5), 8.13 (t, *J* = 1.8 Hz, 1H, *H*4), 7.94 (d, *J* = 1.8 Hz, 2H, *H*2), 7.41 (dd, *J* = 8.0, 1.1 Hz, 2H, *H*12), 6.99 (dd, *J* = 7.8, 1.1 Hz, 2H, *H*10), 6.78 (t, *J* = 8.0 Hz, 2H, *H*11). **^13^C NMR** (*d_6_*-DMSO, 100 MHz, 20°C): δ = 168.4 (*C*6), 148.8 (*C*8), 147.1 (*C*9), 140.6 (*C*3), 125.7 (*C*2), 119.9 (*C*10), 119.5 (*C*11+12), 118.3 (*C*7), 113.5 (*C*4), 94.9 (*C*1) **ESMS** calcd for C_20_H_15_IN_2_NaO_6_ [M+Na]^+^ : 528.9867, found 528.9856. **HPLC:** *R_t_* = 11.49 min (Method A). **IR:** ν = 3360, 1648, 1589, 1533, 1462, 1436, 1333, 1256, 1184, 849, 730 cm^-1^

**
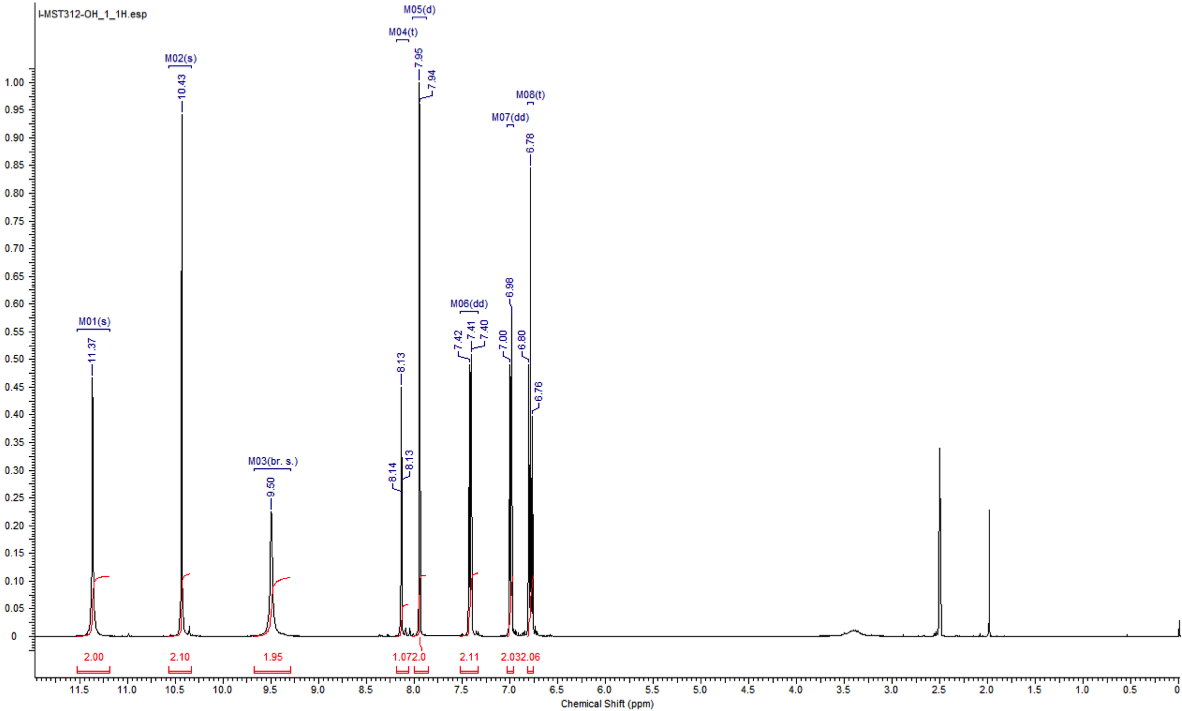
**

**
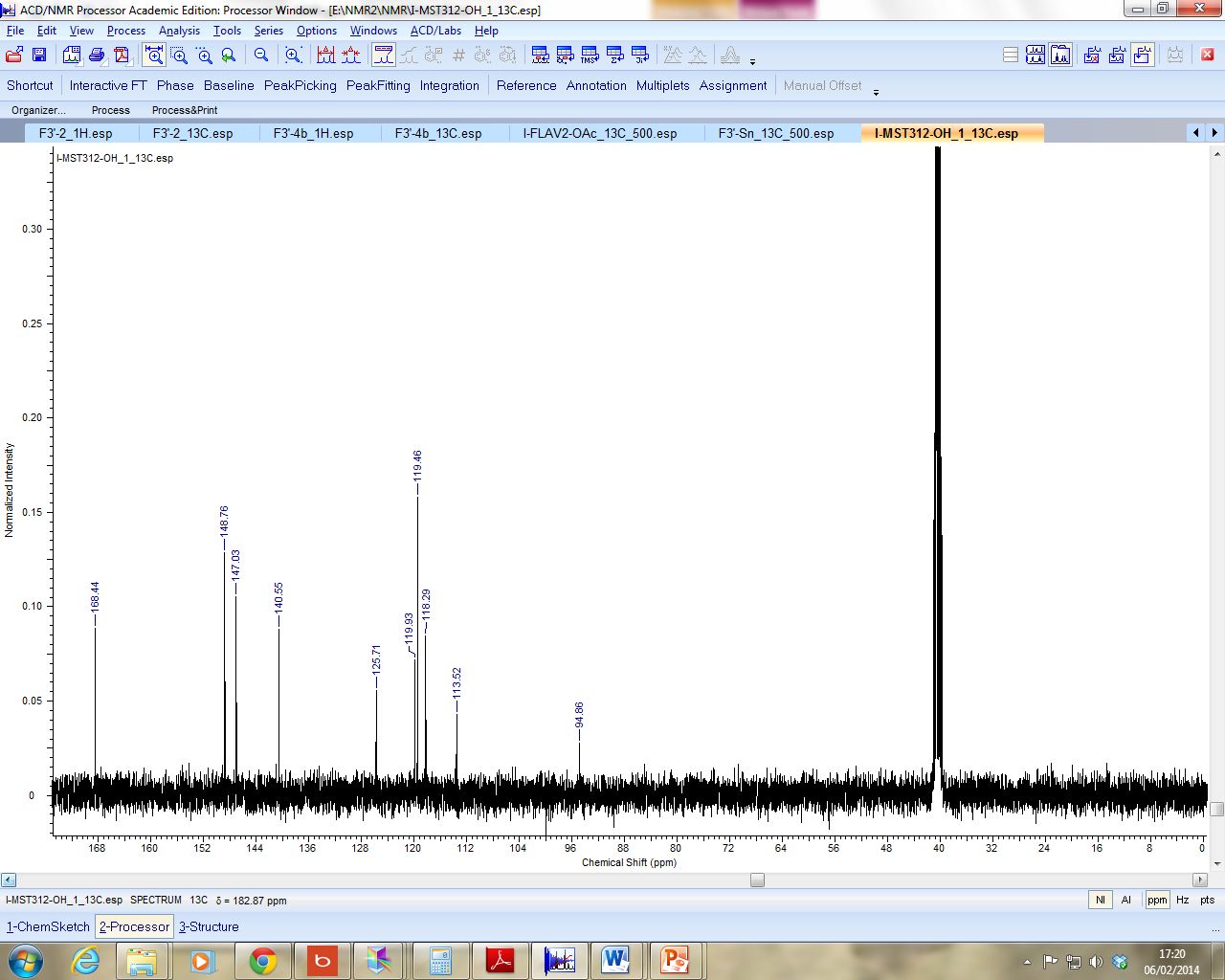
**

**2.10. *N*,*N*'-(5-(Trimethylstannyl)-1,3-phenylene)bis(2,3-diacetoxybenzamide) (6f)**

**^1^H NMR** (CD_2_Cl_2_, 500 MHz, 20°C): δ = 8.03 (s, 3H, *H*4, *H*5), 7.68 (dd, *J* = 7.6, 1.6 Hz, 2H, *H*12), 7.43 (d (84%), *J* = 1.6 Hz with Sn satellites (*J*^2^_Sn-H_ = 54 and 56 Hz (16%), 2H, *H*2), 7.39 (t, *J* = 7.8 Hz, 2H, *H*11), 7.36 (dd, *J* = 8.0, 1.8 Hz, 2H, *H*10), 2.32 (s, 6H, *H*14), 2.31 (s, 6H, *H*16), 0.33 (s (84%), with Sn satellites (*J*^2^_Sn-H_ = 54 and 56 Hz (16%)), 9H, Sn(C*H*_3_)_3_).**^13^C NMR** (CD_2_Cl_2_, 125 MHz, 20°C): δ = 168.8 (C*15*), 168.6 (C*13*), 163.4 (C*6*), 145.4 (C*3*), 143.7 (C*9*), 140.6 (C8), 138.5 (C*1*), 131.3 (C*7*), 127.4 (C*11*), 127.2 (C*12*), 126.8 (C*10*), 123.6 (C*2*), 112.3 (C*4*), 21.0 (C*14/16*), 20.9 (C*14/16*), -9.2 (Sn(*C*H_3_)_3_). **ESMS** calcd for C_31_H_32_N_2_NaO_10_Sn [M+Na]^+^ : 735.0977, found 735.0994. **IR:** ν = 3341, 1773, 1676, 1590, 1535, 1460, 1425, 1371, 1203, 1018 cm^-1^. **HPLC:** *R_t_* = 12.46 min (Method A).


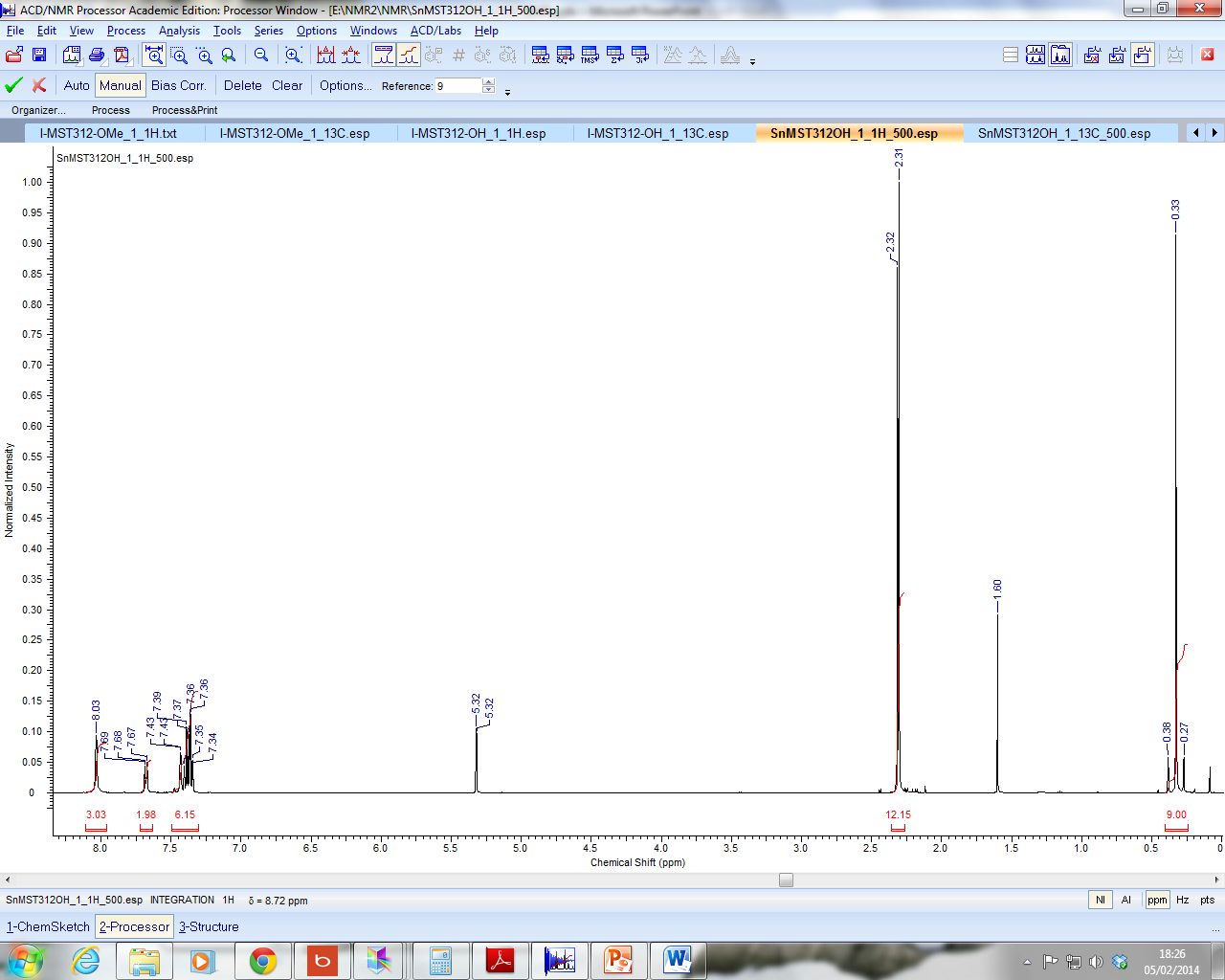

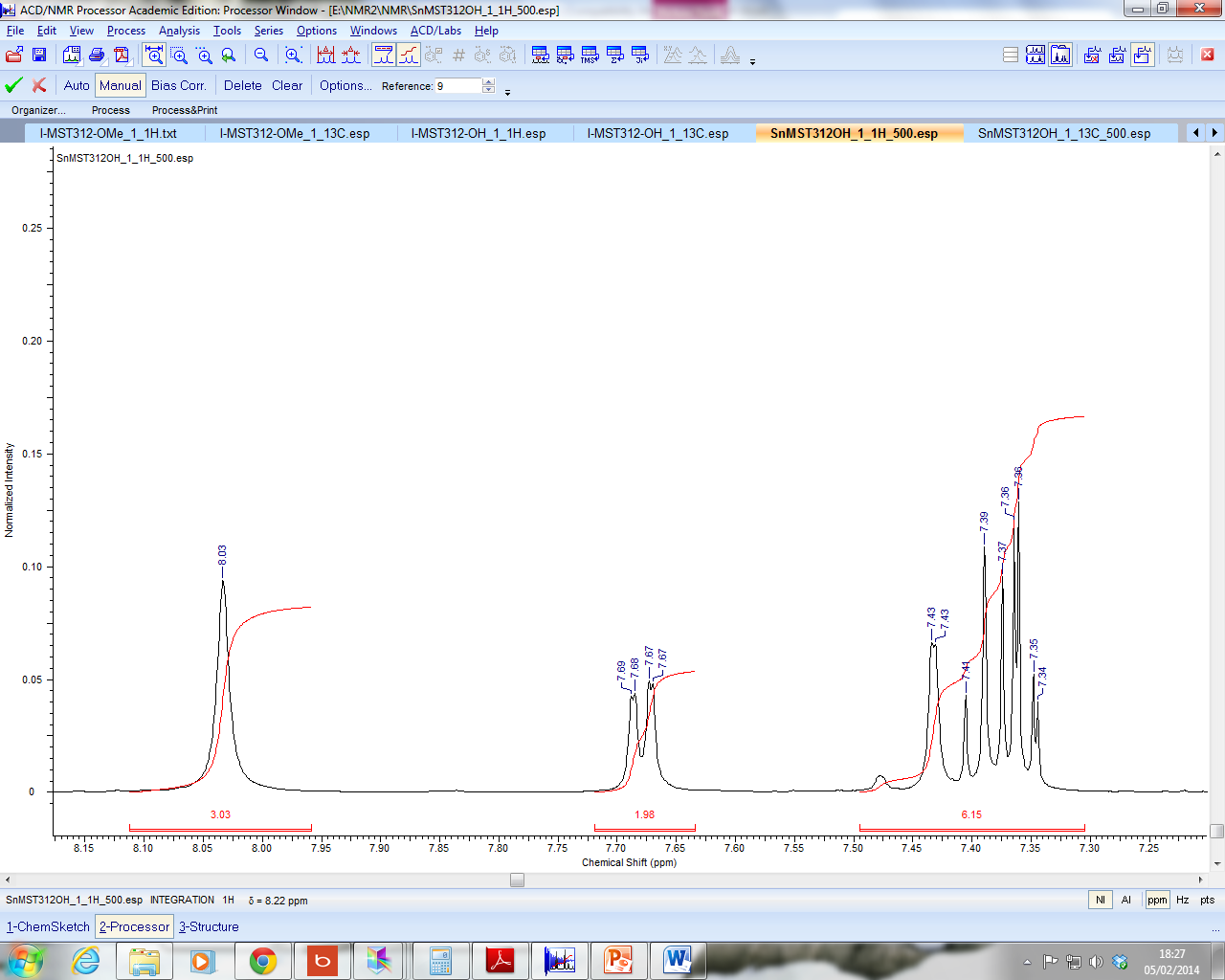


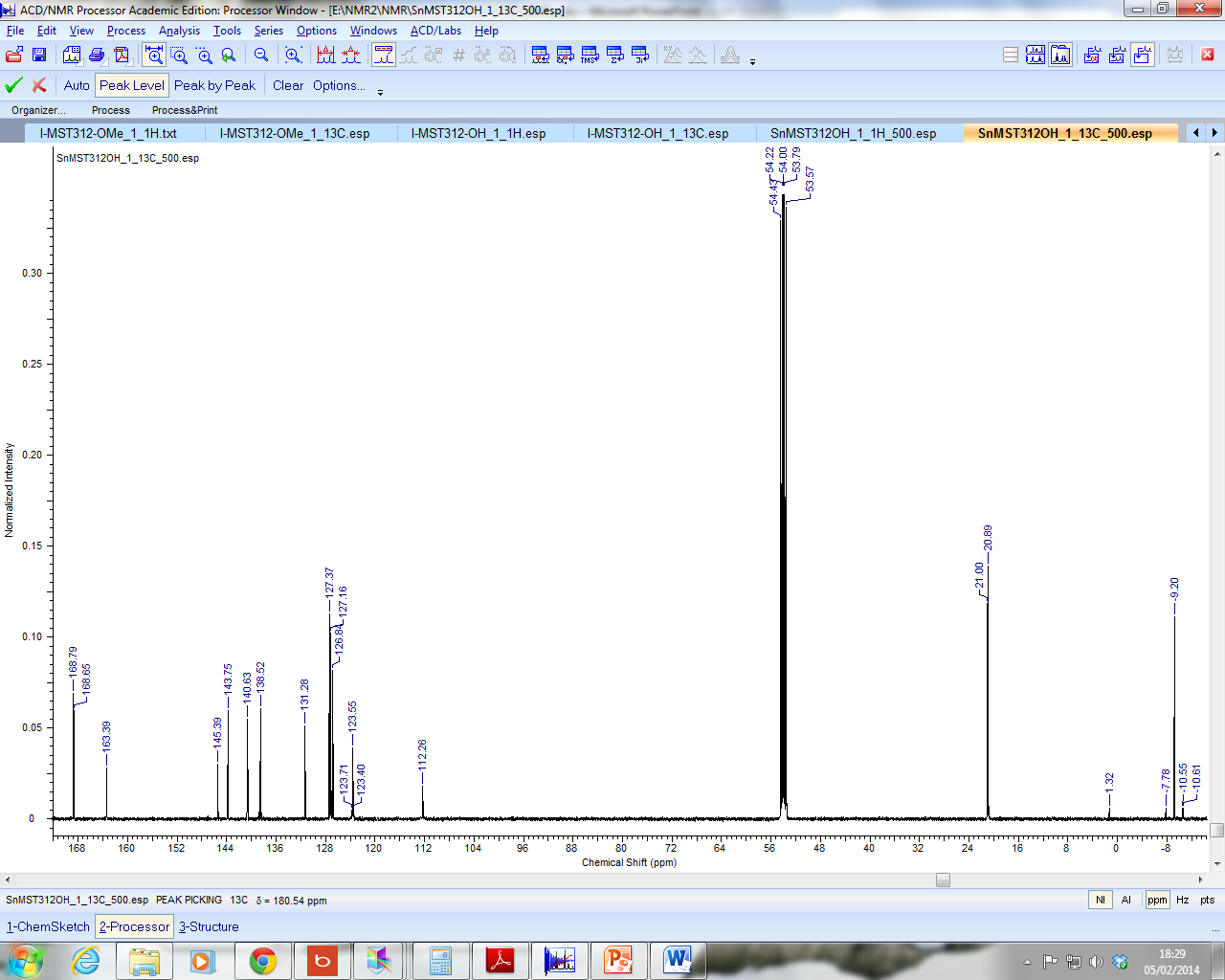

**3. TRAP Protocol**

**3.1. Materials**

TRAPeze XL Telomerase Detection kit (Millipore #S7707); 96-well flat black plate (Costar #3915); Tecan Infinite Fluorescence plate reader (M2000); Read buffer (10mM Tris-HCl pH 7.4, 50mM NaCl; 2mM MgCl_2_); Jumpstart-Taq polymerase (Sigma-Aldrich #D9307-50UN); BIBR1532 (Tocris #2981). 0.1 M Glycine, pH 2.5; Lysis buffer (25 mM KCl; 5 mM MgCl_2_; 10 mM Tris-HCl; 0.5 % NP-40); 0.1 M NaOH; Wizard II Gamma counter. Materials: 1% Methylene blue solution Materials: 1% CHAPS lysis buffer (EMD-Millipore #S7707); BCA Assay kit (Thermoscientific #23225), Bovine serum albumin (Sigma-Aldrich #A7906)

**3.2. Cell culture**

The cells lines employed in this work are the breast MDA-MB-435 carcinoma, and osteosarcoma (U2OS) derived cells. Cells were obtained from the American Type Tissue Culture Collection. Cells were maintained in Dulbecco’s Modified Eagle’s Medium (Sigma-Aldrich #D5796) supplemented with 10 % fetal bovine serum (Gibco #10270) and 1 % penicillin/streptomycin/glutamate (Sigma-Aldrich #G1146). Cells were maintained below confluency by regular passaging using 0.05 % trypsin-EDTA (Gibco #25300-054) and were replaced upon reaching passage 25. Cells were regularly checked for Mycoplasma infection using a MycoAlert testing kit (Lonza #LT07), according to the manufacturer’s instructions.

**3.3. Cell lysate preparation**

Cells were grown to 80 % confluency, harvested with trypsin and counted using a Neubauer haemocytometer. Cells were pelleted (160 x g; 10 minutes; 4 °C), washed with PBS and re-pelleted. CHAPS lysis buffer was added (200 μL/106 cells) and cell pellets incubated on ice for 30 minutes. Lysates were then centrifuged (12000 x g; 20 minutes; 4 °C) to remove insoluble material. Lysate protein concentration was determined using the bicinchoninic acid (BCA) mediated reduction of copper, following the manufacturer’s guidelines. The optical density of lysate titrations and a standard series of bovine serum albumin was analysed at 562 nm following incubation with BCA reagents A (bicinchoninic acid) and B (cupric sulphate). Lysates were aliquoted and snap frozen on dry-ice, before storage at -80 °C.

**4. Telomerase inhibition curves of compounds 1-6**

**5. Cellular/Nuclear uptake plots**

**6. Clonogenic survival plots**

a)

b)

Figure 1: Clonogenic survival data for a) MDA-MB-435 and b) U2OS cell lines after 4h treatment with increasing activity concentrations of [^123^I]-(**6**)

Figure 2: Clonogenic survival curves for a) MDA-MB-435 and b) U2OS cell lines after 24h treatment with increasing concentrations of MST-312 and (**6**)
